# Tin can milling: low-tech mechanochemical synthesis of plant-based prepolymers incorporating perfluoropyridine

**DOI:** 10.1039/d5ra03019f

**Published:** 2025-09-16

**Authors:** Jason Pulfer, Miriam Aldom, Maxime Colpaert, Tim Storr, Chadron M. Friesen

**Affiliations:** a Trinity Western University 22500 University Drive V2Y 1Y1 Langley British Columbia Canada chad.friesen@twu.ca; b Simon Fraser University 8888 University Drive V5A 1S6 Burnaby British Columbia Canada; c ICGM, Université de Montpellier, CNRS, École Nationale Supérieure de Chimie de Montpellier Montpellier Cedex 5 34293 Montpellier France

## Abstract

Perfluoropyridine (PFP) is a fluorinated small molecule heterocycle which can undergo a variety of substitutions in the 2-, 4-, and 6-position to afford rationally designed prepolymers. PFP is known to undergo mechanochemical substitutions, however ball mills have a large start-up cost and are bulky, creating a barrier of entry for researchers. We sought to provide a low tech, affordable, reproducible, and space-saving methodology towards general mechanochemistry while retaining the ability to work on gram scale. Herein we report the successful application of tin can milling (TCM) towards the gram scale synthesis of 4-tetrafluoropyridines (24 examples, up to ≥99% conversion) using a tomato paste can, aluminum beads, a rubber stopper, and agitation with a Burrell Wrist Action™ shaker unit. This approach eliminates problems of scalability with a mortar and pestle and provides a clean method to do benchtop-scale mechanochemistry without additional equipment. We further apply this technique towards making natural product-based prepolymers for polymerization by inverse vulcanization as a proof-of-concept for the use of TCM in monomer synthesis.

## Abstract 2

Perfluoropyridine (PFP) is a fluorinated small molecule which can undergo a variety of substitutions. Facile, mechanochemical substitution of the 4-position of PFP affords natural product monomers which can be subject to inverse vulcanization.

## Graphical abstract

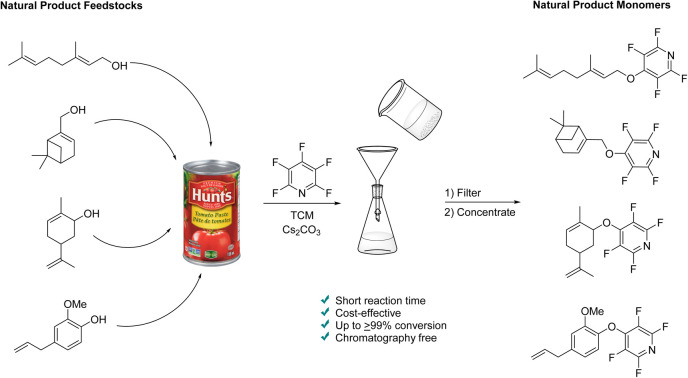

## 1 Introduction

Mechanochemistry is widely recognized as a green, solvent-free methodology that has been applied to C–H bond activation,^1^ metal catalysis,^2^ polymer degradation and morphology tuning,^3^ waste management,^4^ hydrometallurgy of sulfides,^5^ and supramolecular assemblies.^6^ Mechanochemistry is distinct in terms of reactivity and has been observed to produce different products compared to traditional solution state methods, in some cases giving the kinetic product *versus* the thermodynamic product observed in solution.^7^ Grinding with a mortar and pestle is one of the first chemical transformations recorded, making mechanochemistry one of the oldest known types of reactions.^8^ Mechanochemical transformations in the academic laboratory are typically performed in a planetary ball mill or mixer mill in a hardy milling jar.^9^ Twin screw extrusion (TSE) has been applied towards making a “flow”-type reactor, making mechanochemistry viable as a continuous process industrially.^10^ TSE has been used by MOF (metal–organic framework) Technologies to achieve mechanochemical synthesis of MOFs at a rate of up to 15 kg h^−1^,^11^ and solvent free Michael additions and Knoevenagel condensations have been demonstrated up to 0.5 kg h^−1^.^12^

Perfluoropyridine (PFP) is a small molecule fluorinated building block which is readily synthesized from commercially available feedstocks.^13,14^ PFP exhibits diverse reactivity, acting as a protecting group for alcohols,^15^ facilitating amide and ester couplings,^16^ cross-coupling chemistry,^17^ and hydrodefluorination.^18^ PFP is highly regioselective in nucleophilic aromatic substitutions, substituting in the *para* position, followed by further substitution in the *ortho* positions,^19^ making it an excellent candidate for thermoset and step-growth networks.^20–23^ PFP is known to undergo mechanochemical transformations, as shown by Schumacher *et al.* in the C–N bond coupling of PFP and NH-sulfoximines to form *N*-(tetrafluoropyridyl) sulfoximines in solvent free conditions with KOH at 25 Hz over 90 minutes.^24^ Friesen *et al.* discovered that PFP is capable of undergoing nucleophilic aromatic substitution reactions in a ball mill at 80 Hz with a cesium carbonate matrix, attaining substitution in the 2-, 4-, and 6-positions.^25^ This technique was used to synthesize prepolymers consisting of PFP with various glycol spacers, which were then polymerized with bisphenols to attain insoluble polymers.

Another type of polymerization which is found to be of interest and viable is inverse vulcanization. It is a method to induce polymerization of a material with elemental sulfur and an unsaturated organic molecule to make copolymers without solvents or radical initiators.^26^ Kang *et al.* found that sulfur monochloride could also induce similar inverse vulcanization of an unsaturated monomer with two olefin groups to produce linear polychlorosulfides that display optical transparency, low birefringence, and high refractive index (>1.6) in the visible and near-infrared spectrum.^27^ Further, oligomerization of natural products such as limonene have been demonstrated with elemental sulfur in a planetary ball mill, affording soluble species without requiring the high heat (>160 °C) of a typical inverse vulcanization reaction.^28^

In exploring mechanochemical substitution of PFP, we determined that there does not exist a generally affordable laboratory scale device to bridge the gap between a mortar and pestle and benchtop mills. As price point can be a deterrent from exploring mechanochemistry, we sought to develop a methodology that is space-saving, low tech, cost effective, and gram scalable for simple mechanical grinding reactions on the bench top. Perfluoropyridine is a useful model for this transformation, is relatively affordable, and has simple ^19^F NMR patterns to determine substitution. PFP also is useful for polymerization applications due to its 3 active sites, making mechanochemistry a valuable tool for both organic and polymer chemists alike.

## 2 Results and discussion

### 2.1 Reaction scope and synthesis of monomers

**Fig. 1:**
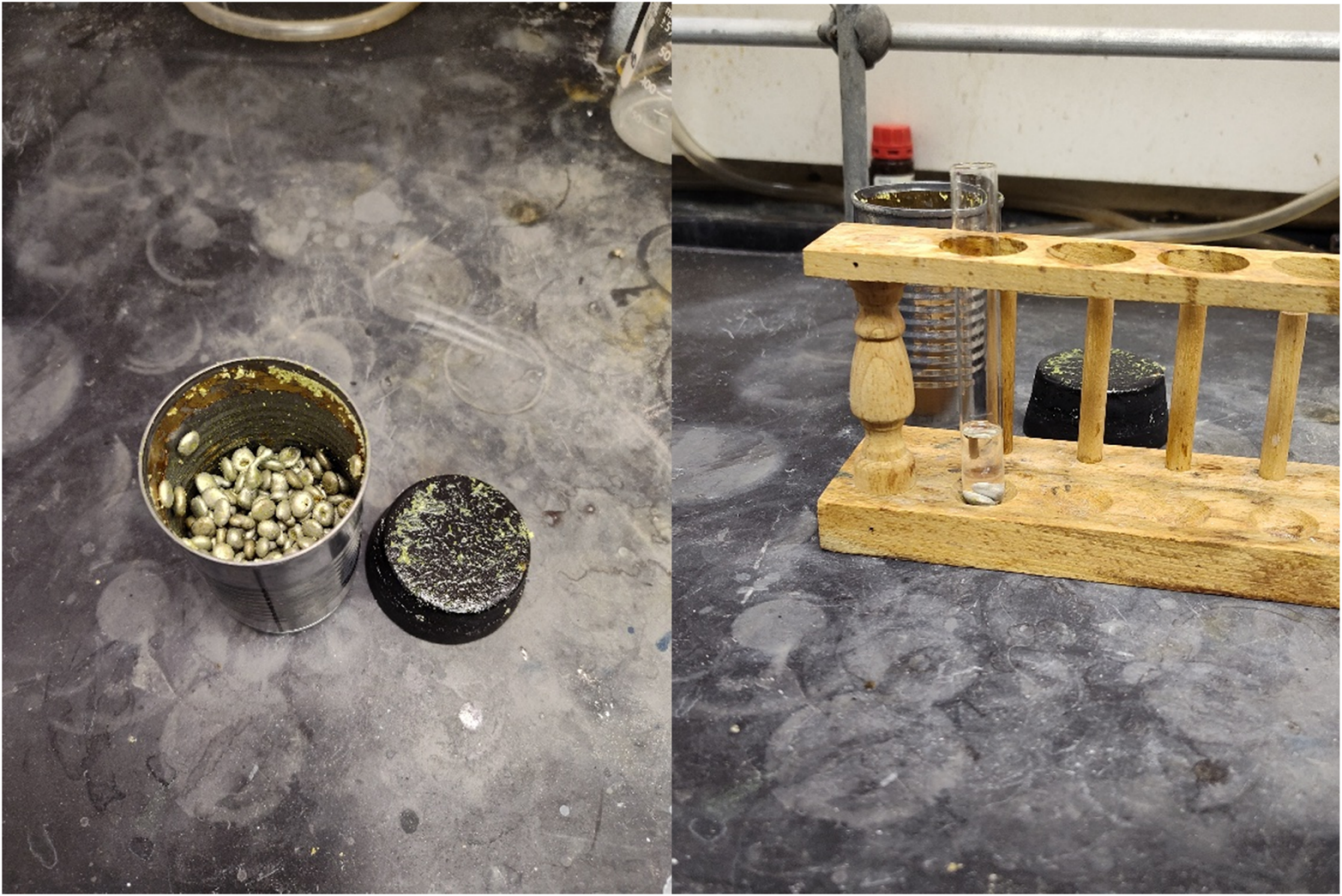
Tomato paste milling jar after grinding a reaction with eugenol as a nucleophile (left) and solvent extraction of beads with chloroform for analysis of the ether product 28 (right).

Mechanochemical reactions typically make use of a ball mill, twin screw extruder (TSE), or a mortar and pestle. In previous work our group discovered that PFP substitutes quite readily using a mortar and pestle,^29^ and Friesen *et al.* successfully demonstrated substitution using a ball mill.^25^ Attempts to scale up our mortar and pestle synthesis to gram scale resulted in drastically lower yields compared to millimolar scale. This led to several questions: (1) can low-tech mechanochemistry be done on gram scale, (2) can mechanochemistry be selective for the 4-position of the PFP ring, and (3) is there intermediate technology between a mortar and pestle and a mill which can initiate smaller scale mechanochemical reactions in an affordable manner? In pursuing these questions, we arrived at the elegant solution of a Hunt's tomato paste (2′′ diameter × 3 7/16′′ height, or 5.1 cm diameter × 8.7 cm height) can as a milling jar fitted with a No. 11 rubber bung (commonly found for home brewing vessels) as a cap (see Fig. 1). We were inspired by the design of a rock crusher apparatus and utilized pea gravel commonly found at home improvement or construction retailers as a grinding medium. Rock dust quickly became an issue, and Lab Armor™ aluminum beads were substituted to mitigate dust formation. Cesium carbonate was chosen as a base due to past literature proving its success in mechanochemical reactions with PFP,^25^ and other common bases, such as K_2_CO_3_ and K_3_PO_4_, have been found to display low yields in reactions of PFP with sulfonamides.^24^

**Scheme 1:**
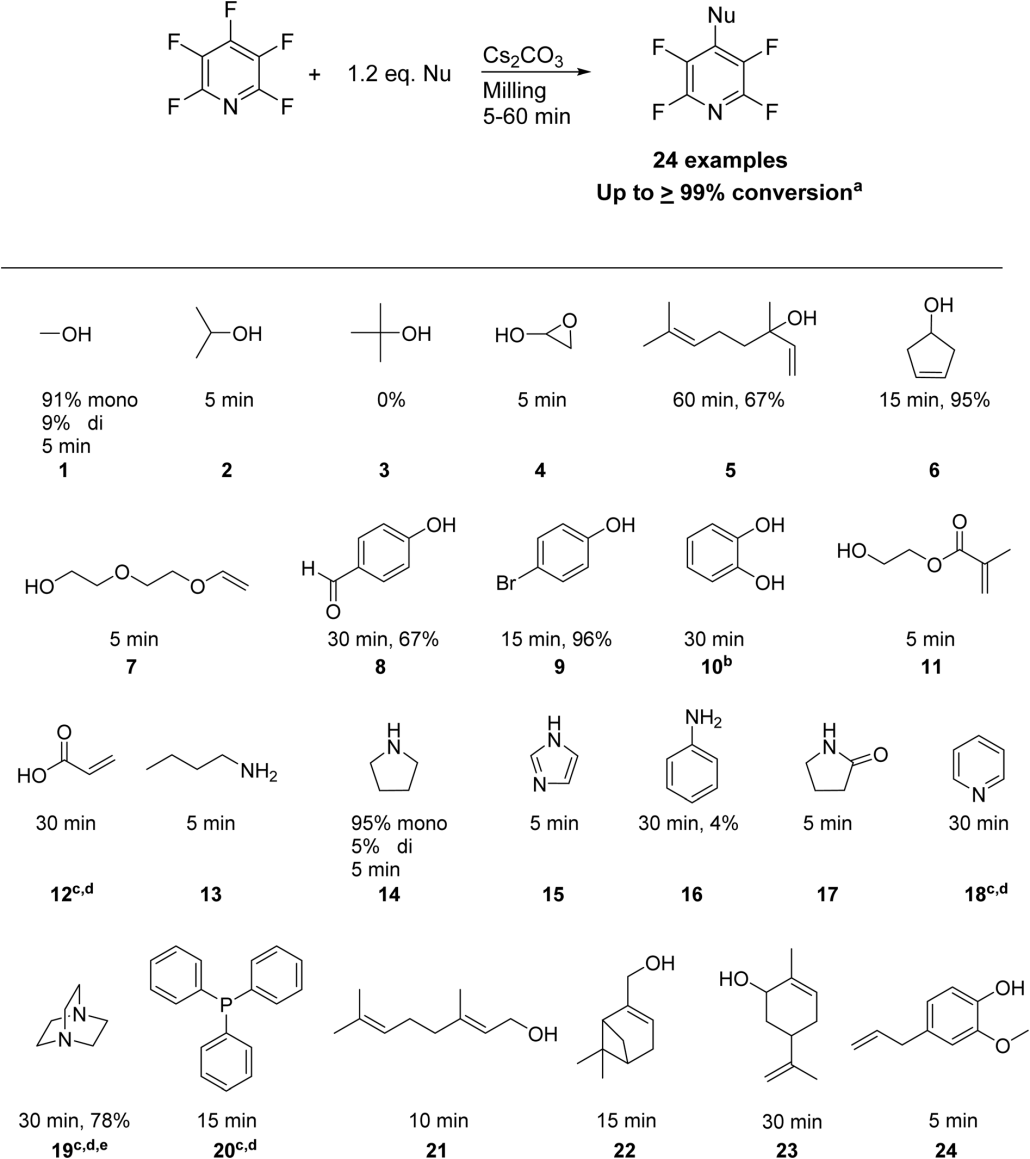
General reaction conditions, scope of nucleophiles and accompanying conversions with reaction time. ^*a*^Yields are quantitative by ^19^F NMR unless otherwise indicated. ^*b*^Yield represents substitution on both positions of the catechol as excess PFP was used. ^*c*^Yield represents conversion to 4-hydroxytetrafluoropyridine based on past literature.^30^^*d*^Water-soluble fraction of the aluminum beads used to determine conversion. ^*e*^Yield represents relative to 2 equivalents of PFP used.

**Fig. 2:**
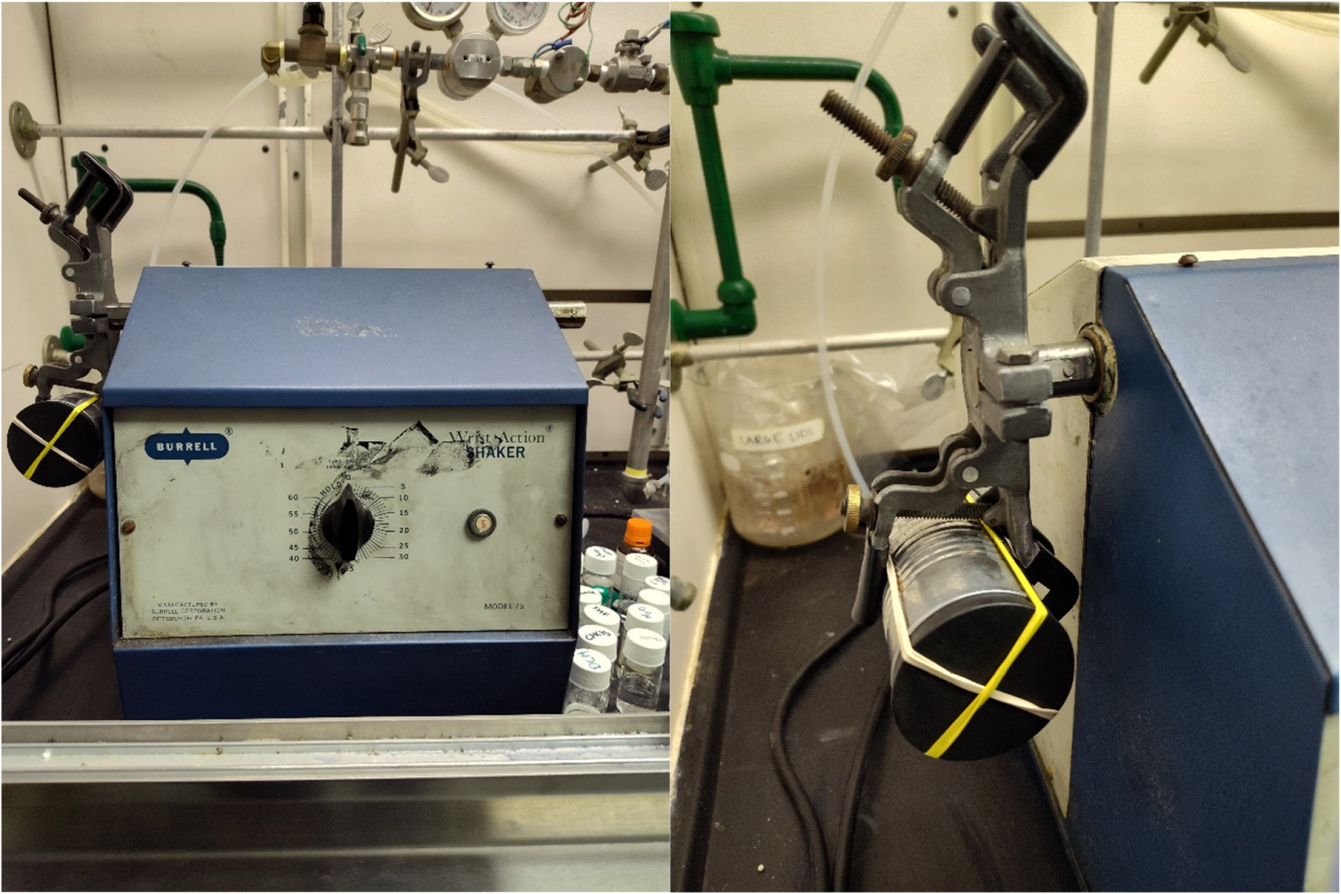
Burrell Wrist Action™ Shaker unit with variable time dial for application in TCM (left) and close-up of the reactor setup involving a tomato paste can fitted with a No. 11 bung secured with elastic bands.

Marbles or ball bearings are equally suitable replacements as milling balls. Manual shaking was successful in the case of 4 (see Scheme 1) but showed variability between trials dependent on the operator. A Burrel Wrist Action™ shaker unit was used to standardize shaking force and frequency, and a scope of nucleophiles was tested to determine the limits of S_N_Ar mechanochemistry in tin can milling (TCM) (see Fig. 2).

**Fig. 3:**
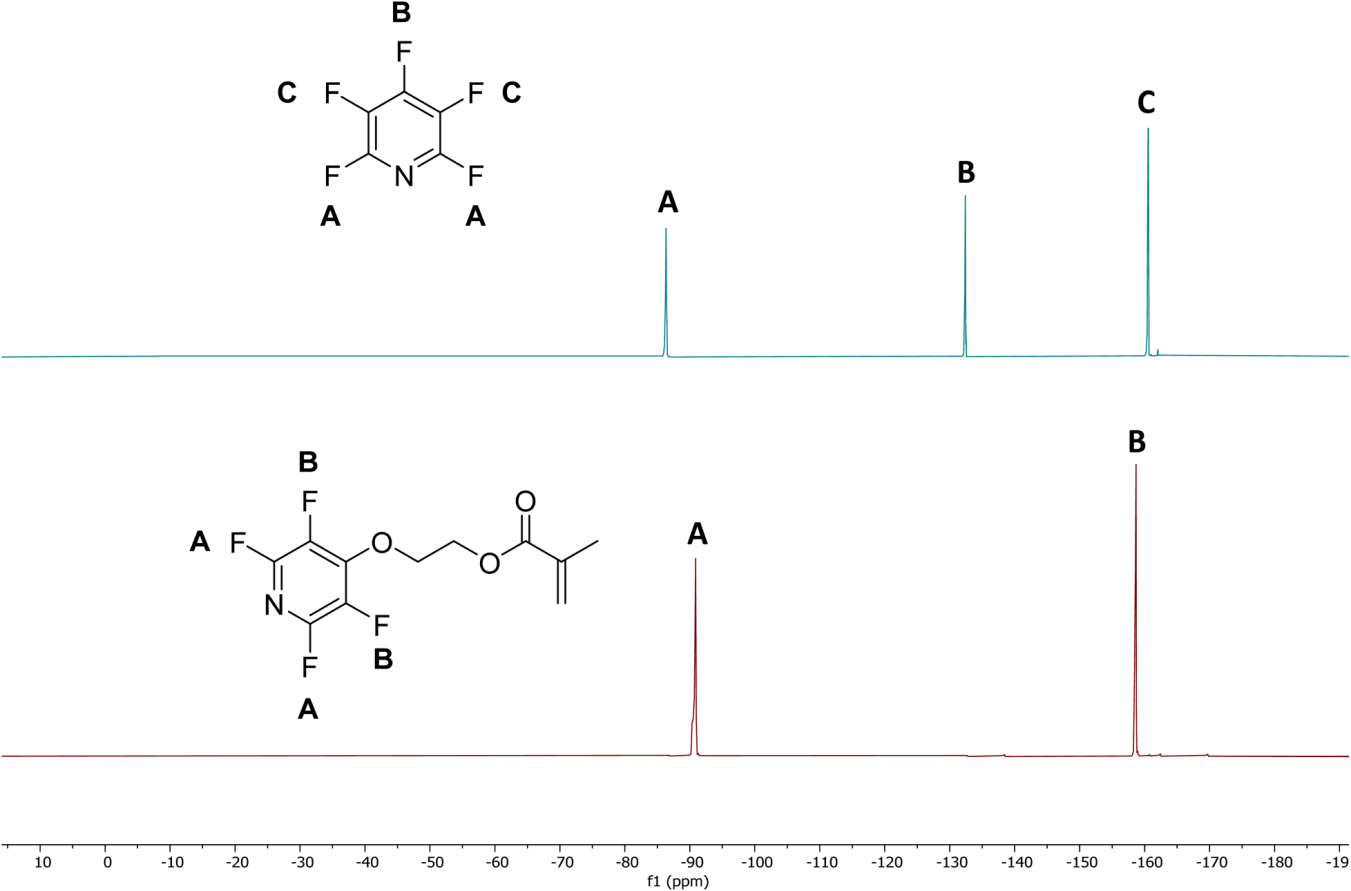
^19^F NMR patterns in CDCl_3_ of the 4-tetrafluoropyridine methacrylate derivative 11 after TCM (bottom) compared to ^19^F NMR of PFP (top).

Samples were milled and tested at the 5, 15, 30, and, in one case 60-minute marks by extracting the deposited reaction mixture off 2–3 of the beads with a suitable deuterated solvent to estimate conversion by ^19^F NMR. 4-Tetrafluoropyridines show diagnostic peak patterns compared to the starting material (see Fig. 3). PFP shows 3 distinct signals at −86.75 ppm, −132.8 ppm, and −160.98 ppm (referenced to CFCl_3_ at 0.00 ppm) with a diagnostic 2 : 1 : 2 ratio of integrations. The 4-tetrafluoropyridines show only 2 signals at ∼−90 ppm and ∼−158 ppm with a 1 : 1 integration, and these signals can be used to determine conversion to the desired product.

Most nucleophiles underwent complete conversion to the desired product within 60 minutes, apart from *tert*-butanol which gave 0% conversion (see Scheme 1). Past literature has found that mechanochemical activation of PFP is controllable by stoichiometry to attain mono-, di-, and tri-substituted derivatives,^25^ however in our hands we have found that lower frequency milling *via* TCM controls reaction regioselectivity to effectively the mono-substitution only. Methanol and pyrrolidine are the only nucleophiles tested which provide further substitution in the 2-position (9% and 5% conversion to di-substituted product, respectively). Further, all reactions were found to be scalable up to 2 grams, providing a facile methodology to create large amounts of substituted PFP with short reaction time. The main advantage to TCM over traditional mechanochemical methods is that it provides similar results to a mortar and pestle, but allows for gram scalability, reproducibility, cost effectiveness, and can be readily employed for reactions which do not require high energy input.

Generally, cyclic structures tend to exhibit lower reactivity than their analogous linear structures. 21 and 22 are primary allylic alcohols, however 22 requires 5 minutes more reaction time. Likewise, 23 requires 30 minutes to reach completion, but 6, which is less sterically hindered, requires just over 15 minutes. 2 and 4 are also secondary alcohols but can reach completion within 5 minutes, indicating that steric bulk is a significant factor in reducing reactivity. Highly activated aromatic nucleophiles, such as 24, require only 5 minutes to reach completion, however less activated aromatic nucleophiles require longer reaction time, with 9 reaching completion just after 15 minutes. Deactivated aromatic nucleophiles react very slowly by comparison, with 8 reaching 67% conversion after 30 minutes of shaking.

**Scheme 2:**
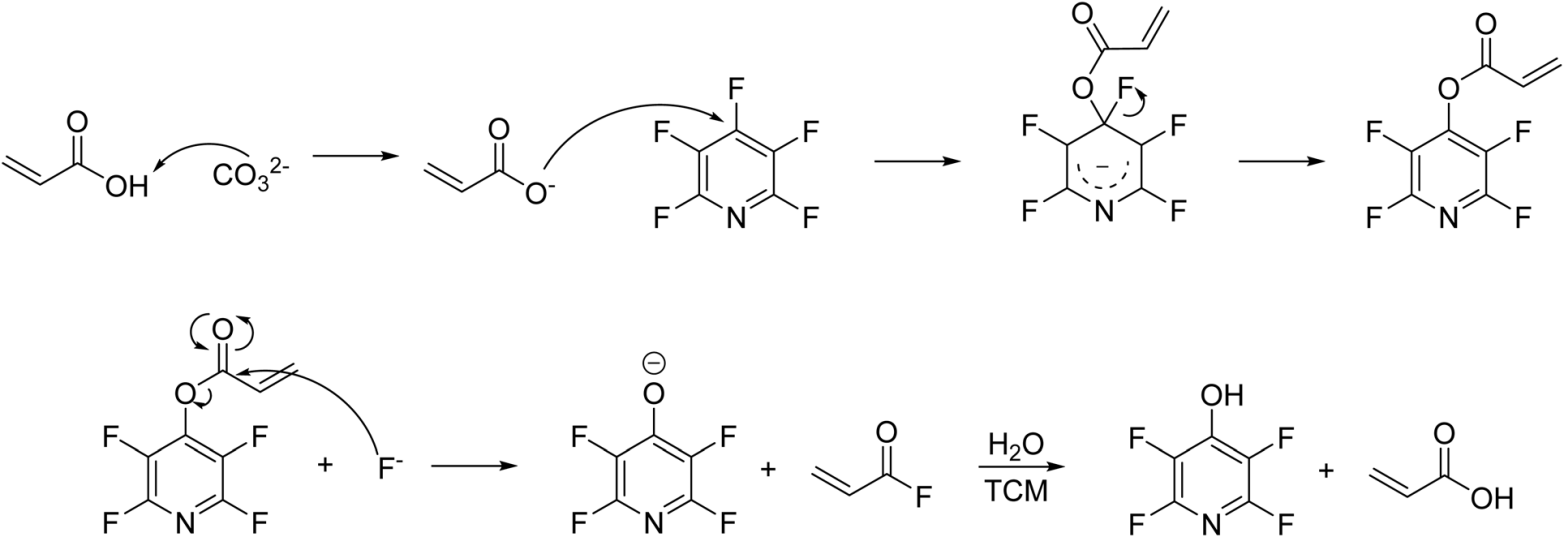
Postulated mechanism of the formation of acyl fluoride and 4-hydroxytetrafluoropyridine.

12 displays substitution through ^19^F NMR, however the extract was only soluble in water. PFP is known to form acyl fluorides in the presence of organic acids and base *via* ester formation, followed by nucleophilic attack of the fluoride on the ester to release 4-hydroxytetrafluoropyridine and the corresponding acyl fluoride (see Scheme 2).^16^ The acyl fluoride is not observed in our hands, likely due to hydrolysis *via* atmospheric water occurring during milling.

**Fig. 4:**
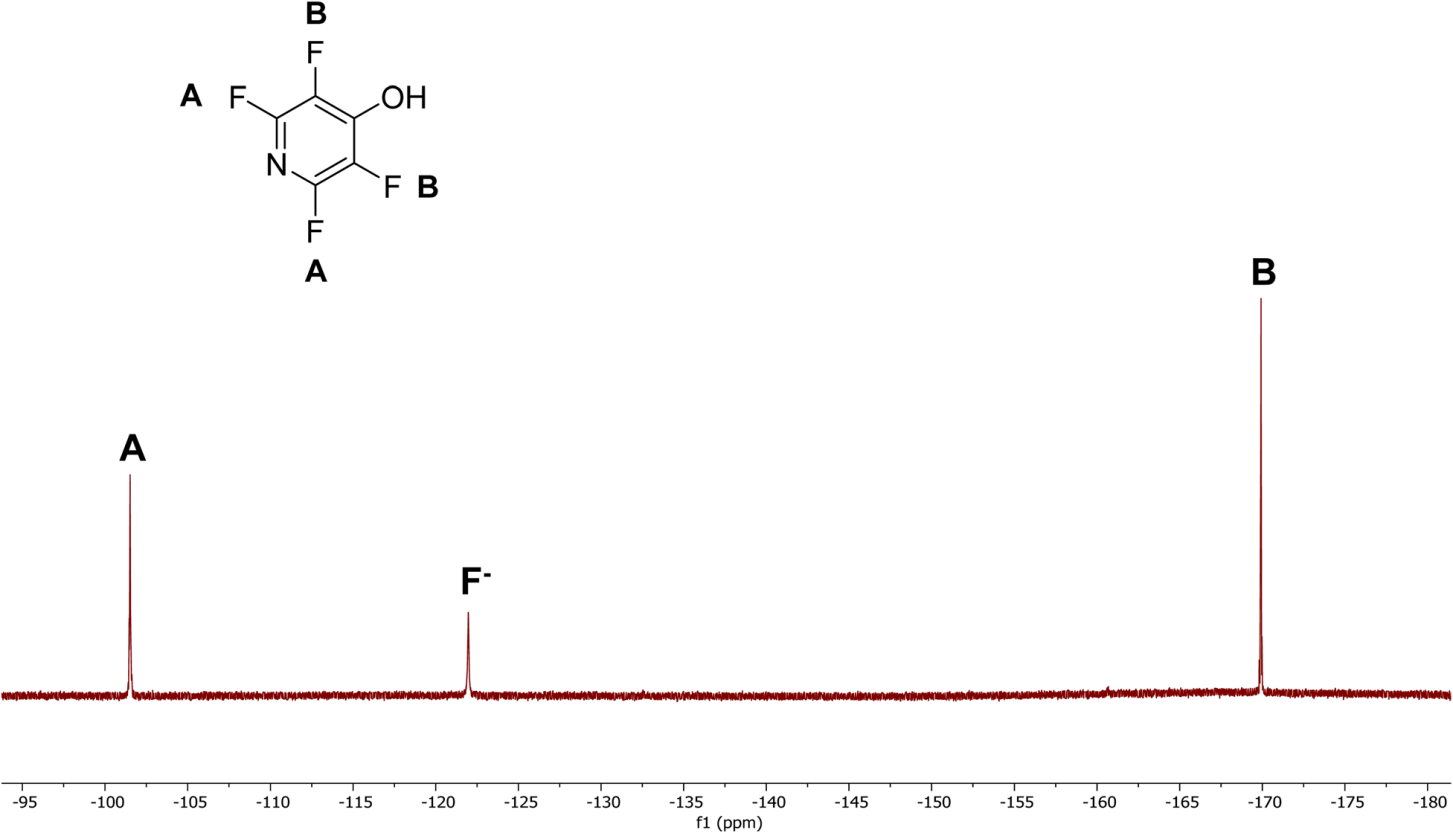
^19^F NMR of 4-hydroxytetrafluoropyridine in D_2_O produced from the reactions of 12, 18, 19, and 20.

Tertiary pnictogens such as 18, 19, and 20 all substitute to form water soluble species. 19 displays 78% conversion with 2 equivalents of PFP. 20 shows a distinct pattern of mono-substitution in the ^19^F NMR in D_2_O at −101.5 ppm and −169.9 ppm with a 1 : 1 integration. An additional peak at −122.0 ppm indicates the presence of free fluoride, indicating substitution has occurred (see Fig. 4). Further review shows that 16, 18, 19, and 20 display the same ^19^F NMR shifts, matching that of 12, indicating that these species form 4-hydroxytetrafluoropyridine based on ^19^F NMR in past literature (ref. 15).

**Fig. 5:**
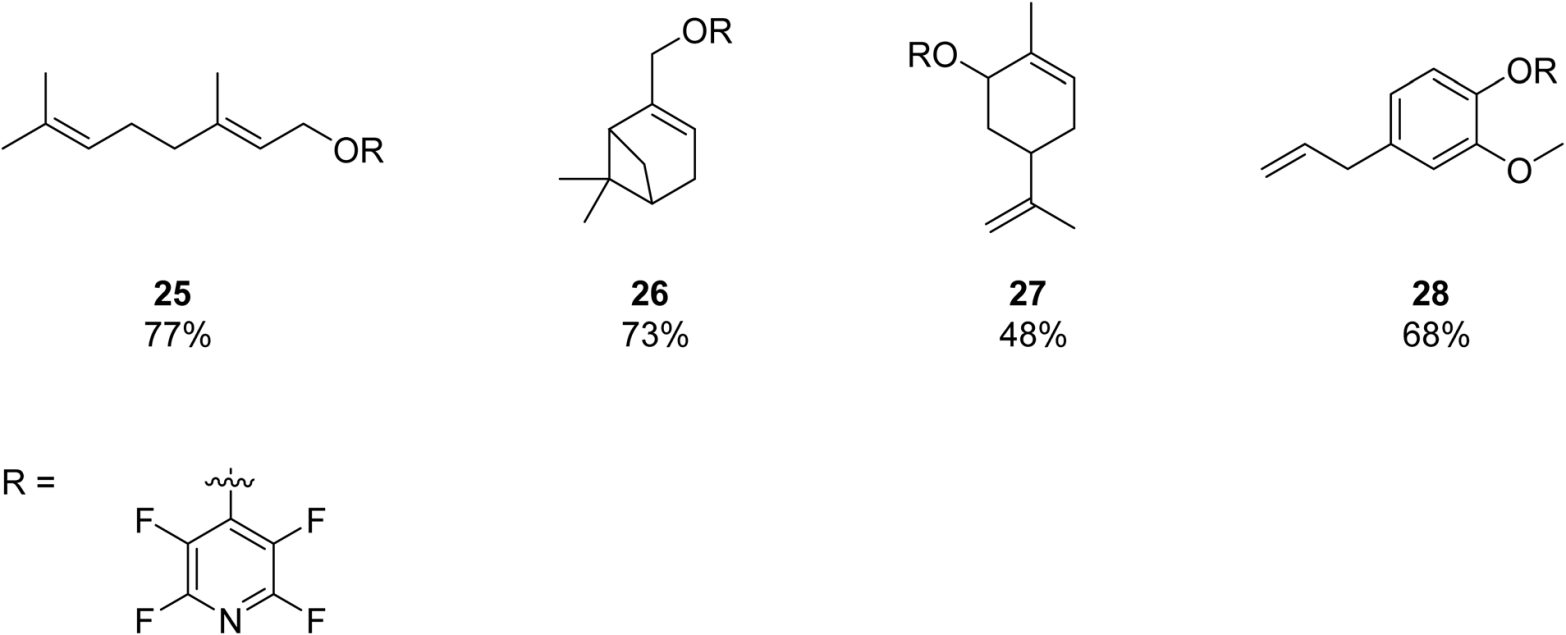
Candidates for inverse vulcanization of PFP with S_8_ and S_2_Cl_2_ and isolated yields.

During this work, it was discovered that none of the isolated samples are amendable to classical mass spectrometry (MS) techniques. Of the isolated samples, only 28 was found to provide a MW peak by EI-MS. Samples 25, 26, and 27 showed fragmentation patterns by EI-MS, indicating that α,β unsaturated PFP ethers are relatively unstable in by this ionization technique (see Fig. 5).

**Fig. 6:**
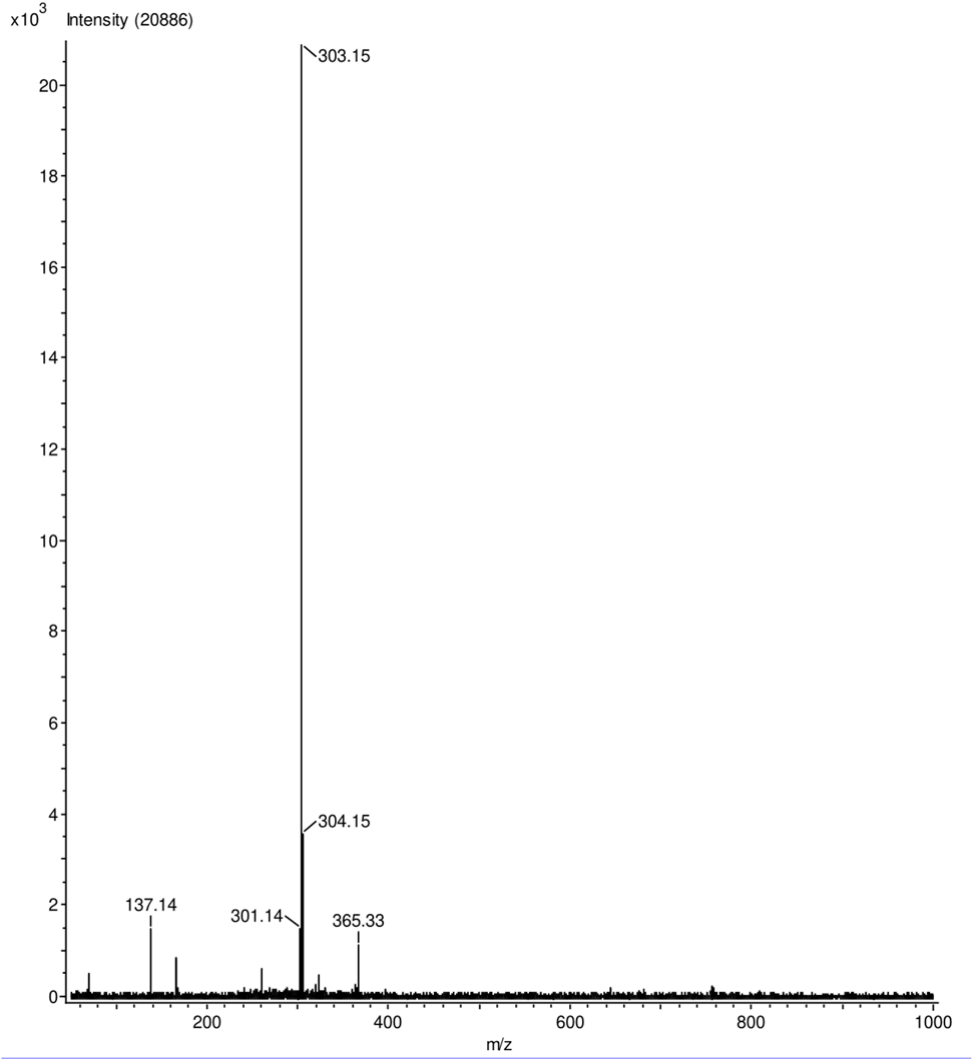
Sample FD-MS spectrum of 25 with MW 303.15 g mol^−1^ (see Fig. 6).

In our hands, field-desorption mass spectroscopy (FD-MS) was found to provide exceptionally clean spectra containing the molecular weight peak and is highly recommended by the authors for similar samples which display fragility under conventional EI and ESI ionization techniques (see Fig. 6). 25 and 28 were additionally observed to decompose on the benchtop over the course of 3 months, indicating thermal or photo instability.

### 2.2 Inverse vulcanization of PFP-containing natural product monomers

**Scheme 3:**
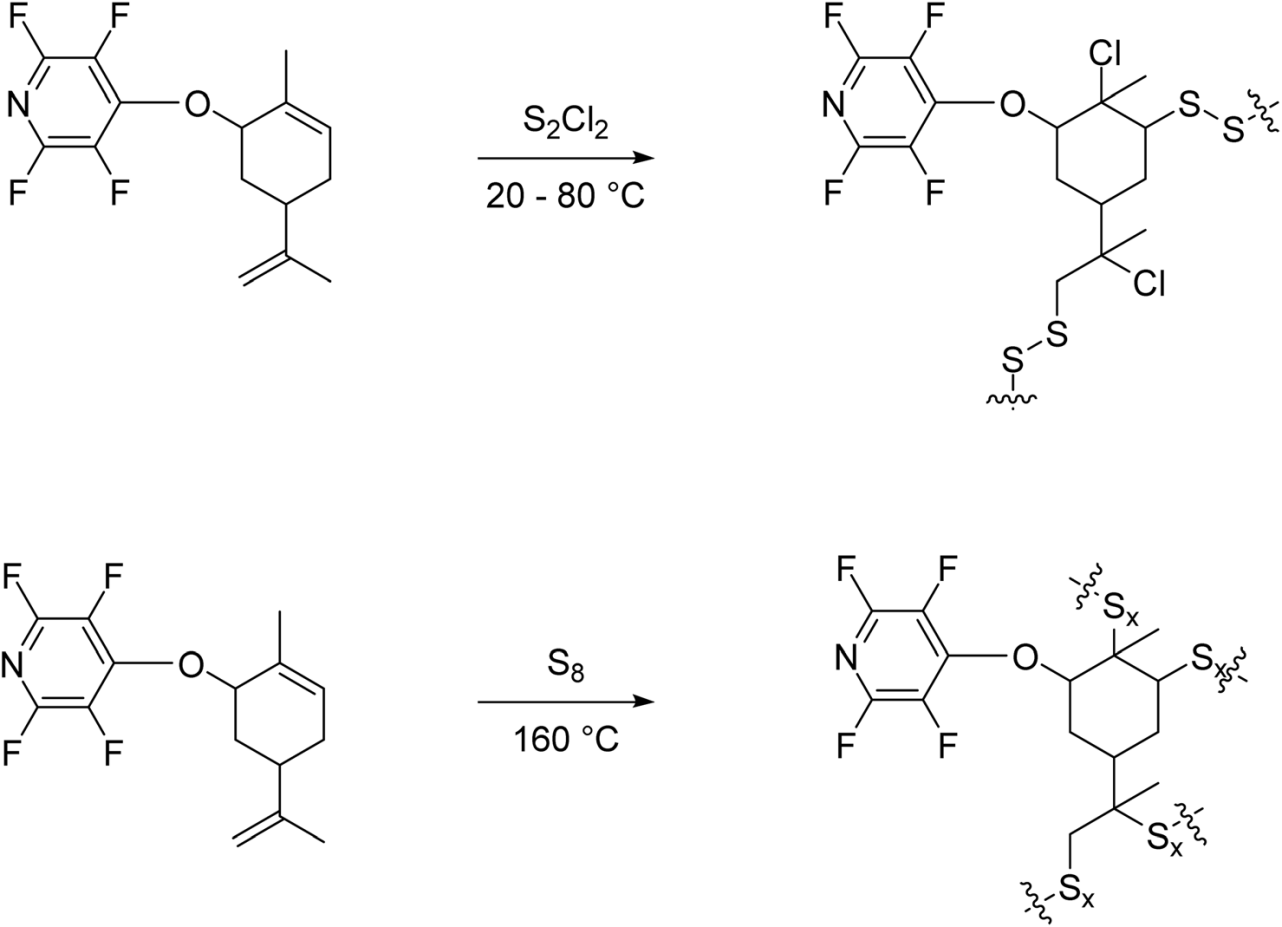
General scheme of the vulcanized materials.

Inverse vulcanization is a term for a crosslinked polymer wherein the organic component can be seen as a crosslinking agent to stabilize long sulfur chains. Inverse vulcanized materials are commonly used in energy generation and storage, environmental remediation, optical devices, repairable material development, and mercury capture.^28,31^ Such materials have been successfully synthesized on kilogram scale to develop high-capacity polymer electrodes for Li–S batteries with 1,3-diisopropenylbenzene as a comonomer.^32^ These sulfur-rich materials have not seen use outside of academic research, however some companies such as ThioTech, Outside the Box Materials, Uberbinder, and Clean Earth Technology have undertaken research in this area to attempt commercial exploitation.^31^ Several natural product derivatives of PFP were envisioned to be viable for inverse vulcanization through reaction with elemental sulfur or with sulfur monochloride due to their unsaturated components (see Scheme 3).

The sulfur monochloride product was tested at room temperature and with heating in bulk, with both air and nitrogen atmospheres investigated. Inverse vulcanizations with elemental sulfur were conducted in bulk and were allowed to proceed until the sample formed a solid at 160 °C, or until 1 week passed, in the case of 28-S_8_. The thermal data and gel-permeation chromatography (GPC) results are summarized in Table 1. Yields for the sulfur monochloride reactions are quite low: vulcanization of 25-S_2_Cl_2_ in tetrahydrofuran (THF) in atmospheric conditions provided the highest isolated yield of 42%, compared to 4% at 70 °C under nitrogen and 20% at room temperature under air. Conversely, 27-S_2_Cl_2_ provides a better yield when cured neat under nitrogen at 70 °C compared to in solution (37% *versus* 12% respectively).

**Table 1:** Thermal data and GPC of the inverse vulcanized polymers produced with 25, 26, 27 and 28

Sample	*T*_onset_ air (°C)	*T*_onset_ N_2_ (°C)	*T*_g_ (°C)	*M*_N_ (g mol^−1^)	*M*_W_ (g mol^−1^)	PDI
25-S_8_	207	209	—	—	—	—
26-S_8_	228	217	−2	—	—	—
27-S_8_	208	206	−5	—	—	—
28-S_8_	227	241	61	—	—	—
25-S_2_Cl_2_ (20 °C, air)	142	143	1	1363^a^	2038^a^	1.5
				45 399^b^	48 252^b^	1.1
25-S_2_Cl_2_ (65 °C, THF air)	168	168	10	842	1983	2.4
25-S_2_Cl_2_ (70 °C, N_2_)	172	173	42	1677	2456	1.5
27-S_2_Cl_2_ (65 °C, THF, air)	162	184	20	457	872	1.9
27-S_2_Cl_2_ (70 °C, N_2_)	202	202	53	1071	2548	2.4

a Low molecular weight distribution peak from GPC.

b High molecular weight distribution peak from GPC.

### 2.3 Gel permeation chromatography (GPC) and thermal analysis of the polymers

25 was chosen to examine various conditions for bulk sulfur monochloride vulcanization and generally forms lower molecular weight oligomers at room temperature and elevated temperature under nitrogen. The best conditions for this reaction appear to be under nitrogen at 70 °C, which gives the highest *M*_N_ value of 1677 g mol^−1^ and a PDI of 1.46. Oddly, room temperature vulcanization of 25 under ambient atmosphere provides 2 peaks in the chromatogram, with an *M*_N_ at 45 399 g mol^−1^ and a PDI of 1.07 as the minor peak (25% by UV detector peak area), indicating the material can undergo significant polymerization to make longer chains given appropriate conditions. 27 mainly produces low molecular weight oligomer (400–1200 g mol ^−1^), likely due to steric bulk of the ring.

The *T*_onset_ (temperature of onset of degradation measured as the intersection of the baseline weight and tangent of the point of maximum gradient of the curve)^33^ (see Table 1) of S_8_ crosslinked materials are consistent at 205–230 °C, with 25-S_8_ showing slightly higher *T*_onset_ values of ∼228 °C in air. Sulfur monochloride vulcanized material gives similar results between air and nitrogen, and 27-S_2_Cl_2_ showing the highest thermal stability when vulcanized at 70 °C under nitrogen (∼201 °C). Compared to 25-S_2_Cl_2_ crosslinked under the same conditions, 27-S_2_Cl_2_ has a ∼40 °C increase in *T*_onset_ at a lower molecular weight (1071 g mol ^−1^ compared to 1677 g mol^−1^), indicating higher stability of the carveol group. Glass transition temperatures (*T*_g_) vary across polymer classes, with S_8_ vulcanized material ranging from −2–61 °C, and sulfur monochloride vulcanized material ranging from 0.6–53.3 °C. In our hands 25-S_8_ does not present a *T*_g_ in the DSC, however, PFP with alkyl units such as 26-S_8_ and 27-S_8_ give low *T*_g_ values of −2 °C and −5 °C respectively, compared to 61 °C for 28-S_8_. This is hypothesized to be due to the rigid nature of the eugenol substituent, indicating that *T*_g_ can be readily modulated in S_8_-vulcanized material, allowing for the production of glassy or more flexible material based on the comonomer. As inverse vulcanized materials are hypothesized to have application in Li–S batteries, materials with highly fluorinated aromatics could be a potential route to lower *T*_g_ values and produce battery films that are capable of low temperature applications.^32^ Materials undergoing S_2_Cl_2_ inverse vulcanization demonstrate an increase of *T*_g_ when vulcanized under N_2_ (42 and 53 °C for 25-S_2_Cl_2_ and 27-S_2_Cl_2_ respectively) compared to normal atmosphere (10 and 20 °C for 25-S_2_Cl_2_ and 27-S_2_Cl_2_ respectively), indicating that higher molecular weight increases the glass transition temperatures, and these materials could have potential use in flexible optical films or as low birefringence materials, similar to those developed by Kang *et al.*^27^

## 3 Conclusion

Perfluoropyridine was found to undergo mechanochemical substitution in up to quantitative conversion through simple, low tech mechanochemistry in a tomato paste can with commonplace grinding media. The approach is gram scalable, cheap, and highly regioselective to the 4-position of the ring. The natural product derivatives of PFP were demonstrated to undergo inverse vulcanization with elemental sulfur and sulfur monochloride, showing utility of the methodology towards prepolymer design and synthesis. Future work will focus on low tech methodologies to rival commercial ball mills using common equipment from the laboratory or hardware store.

## 4 Experimental

### 4.1 Materials

Chemicals were used as received unless otherwise noted. Geraniol (≥97%), eugenol (99%), (−)-carveol (mixture of isomers, 97%), myrtenol (≥95%), pyrrolidine (≥99.0%), pyridine (99.8%), methanol (≥99.6%), isopropanol (≥99.5%), 1,4-diazabicyclo[2.2.2]octane (DABCO) (≥99%), imidazole (≥99.5%), 2-pyrrolidinone (99%), 4-bromophenol (99%), 4-hydroxybenzaldehyde (98%), acrylic acid (≥99.0), glycidol (96%), 2-hydroxyethylmethacrylate (97%), hexanes (mixture of isomers, ≥98.5%), diethyl ether (≥99.0%), ethyl acetate (≥99.5%), chloroform (≥99.8%), dichloromethane (≥99.8%), tetrahydrofuran (≥99.0%), catechol (≥99%), alumina (80–200 mesh, chromatographic grade) and silica (70–230 mesh, 60 Å, for column chromatography) all came from Sigma-Aldrich. Sulfur monochloride (98%) obtained from Sigma-Aldrich and distilled before use, giving an orange liquid. Perfluoropyridine (PFP) (99%), cesium carbonate (99.9%), 3-cyclopenten-1-ol (98%) and diethylene glycol monovinyl ether (98%) were obtained from AK Scientific. Triphenylphosphine (powder, 99%) and sulfur (powder, 325 mesh, 99.5%) came from Alfa Aesar. *tert*-Butanol was obtained from Fischer Scientific. Butylamine (98%) was obtained from EM Scientific. Aniline (99%) was obtained from Anachemia.

### 4.2 Characterization

#### 4.2.1 Spectroscopy

NMR spectra were collected on a Bruker ASCEND III 400 MHz NMR with a Bruker AVANCE III 400 MHz running TopSpin 3.1.6 and processed with MestReNova or TopSpin. ^1^H (400 MHz) and ^13^C NMR (101 MHz) spectra were referenced to an internal standard or CDCl_3_ (7.26 ppm) and ^19^F NMR (376 MHz) spectra were to CCl_3_F (0.00 ppm). Chemical shifts were reported in parts per million (ppm) and coupling constants are reported in Herz to the nearest 0.01 Hz. NMR data is reported as: chemical shift, multiplicity (s, d, t, q, pent, and sext stand for singlet, doublet, triplet, quartet, pentet, and sextet, respectively), coupling constants (Hz), integration, and identity. ^19^F NMR parameters are as follows: 64 scans, acquisition time 1.467 s, relaxation delay 2 s, −43.56 ppm offset, and 237.165 ppm sweep width. Field desorption mass spectroscopy (FD-MS) was conducted on a Jeol JMS-T100GCV AccuTOF GCv 4 g equipped with a field desorption source. Attenuated total reflectance Fourier transform infrared spectroscopy (ATR-FTIR) was performed on a ThermoFisher Nicolet 6700 spectrometer fitted with a Smart iTR sampling accessory and reported in reciprocal centimeters (cm^−1^).

#### 4.2.2 Differential scanning calorimetry (DSC)

Analysis was carried out using a NETZSCH DSC200F3 calorimeter. The calibration was performed using adamantane, biphenyl, indium, tin, bismuth and zinc standards. Nitrogen was used as purge gas. Approximately 10 mg of sample were placed in perforated aluminum pans and the thermal properties were recorded. Data was analyzed using Proteus 8.1 and OriginPro 2021.

#### 4.2.3 Thermal gravimetric analysis (TGA)

Measurements were conducted on a TGA Q50 from TA analysis. Samples were placed in 30 μL aluminum crucibles and heated from 25 to 500 °C at 10 °C min^−1^ under nitrogen or air flow (60 mL min^−1^). Data was analyzed with Origin Pro 2021. *T*_onset_ was defined as the temperature of onset of degradation measured as the intersection of the baseline weight and tangent of the point of maximum gradient of the curve.

#### 4.2.4 Size-exclusion chromatography (SEC)

Analyses of polymers were conducted on a system composed of an Agilent Infinity I pump degasser, oven and detector 1260 VWD G1314F and a Varian 390-LC Multi detector suite fitted with differential refractive index, light scattering, and viscosimeter. The system was equipped with a guard column (Agilent Mesopore, 50 × 7.5 mm) and two columns (Agilent Mesopore and Resipore, 300 × 7.5 mm). The mobile phase was tetrahydrofuran (THF) at a flow rate of 1.0 mL min^−1^. Toluene was added to the samples as a flow marker and samples (typical concentration: 5 mg mL^−1^ and injection 100 μm) were filtered prior to analysis (0.20 μm PTFE filter) and results were calibrated with and poly(methyl methacrylate) (PMMA) standards (550–2 210 000 g mol^−1^) or polystyrene standards (162–371 000 g mol^−1^) using Agilent GPC/SEC v1.2 software.

### 4.3 Preparation

#### 4.3.1 General TCM reaction procedure

For production of mono-substituted perfluoropyridine in the 4-position, ∼95 g of Lab Armor™ aluminum beads were placed in a Hunt's tomato paste can, along with Cs_2_CO_3_ (1.3 eq., 2.390 g, 7.34 mmol), PFP (1.2 eq., 1.098 g, 6.50 mmol), and nucleophile (for geraniol ex. 1 eq., 0.846 g, 5.48 mmol). The reactor was then agitated using a Burrell Wrist Action™ Shaker and monitored using ^19^FNMR at the 5-, 15-, 30-, and 60-minute mark. Reactions were isolated by filtration through a cotton plug and alumina to afford the product as an oil. Reactions for the scope were performed with slight excess of nucleophile (1.2 eq.) to ensure conversion and determine if secondary substitution occurred. Isolated materials were performed with a slight excess of PFP (1.2 eq.) to ensure ease of purification. Note: filtration of the aluminum beads through a glass frit or a glass frit with a Celite plug under nitrogen pressure was unilaterally observed to induce clogging, thereby making filtrations long and tedious. The authors recommend vacuum filtration through a glass funnel with a cotton plug and alumina to ensure a smooth, simple work-up. Prior to the use of a tomato paste can as a milling jar, the can must be washed thoroughly with soap and water, followed by soaking and rinsing with dichloromethane at least 5 times to remove the plastic lining.

##### 4.3.1.1 Synthesis of 25

PFP (1.098 g, 6.50 mmol, 1.2 eq.) was placed in a tomato paste can containing aluminum beads, along with Cs_2_CO_3_ (2.390 g, 7.34 mmol, 1.3 eq.), and geraniol (0.846 g, 5.48 mmol, 1.0 eq.). Quantitative conversion was shown by ^19^F NMR after 10 minutes of agitation. The product was extracted with 3 × 50 mL of chloroform, vacuum filtered through alumina and a cotton plug in a glass funnel, and concentrated to a golden yellow oil (88% isolated yield).

^19^F NMR (CDCl_3_, 376 MHz, CCl_3_F) *δ*: −91.6 (m, 2F, 2,6-position C_5_F_4_N), −159.0 (m, 2F, 3,5-position C_5_F_4_N).

^1^H NMR (CDCl_3_, 400 MHz) *δ*: 5.47 (t, ^3^*J* = 6.94 Hz, 1H, –O–CH_2_–**CH**=C–), 5.02 (s, 1H, –**CH**=C(CH_3_)_2_), 5.00 (s, 2H, –O–**CH**_**2**_–CH=), 2.08 (s, 4H, –**CH**_**2**_**CH**_**2**_–CH=C–), 1.74 (s, 3H, –O–CH_2_–CH=C–**CH**_**3**_), 1.66 (s, 3H, –C–(**CH**_**3**_)_2_), 1.59 (s, 3H –C–(**CH**_**3**_)_2_).

^13^C-NMR (CDCl_3_, 100 MHz) *δ*: 147.2 (m, 4-position C_5_F_4_N), 146.1 (s, –CH=**C**(CH_3_)–CH_2_–), 144.3 (dm, ^1^*J* = 242 Hz, 3,5-position C_5_F_4_N), 135.5 (dm, ^1^*J* = 256 Hz, 2,6-position C_5_F_4_N), 132.3 (s, –CH=**C**(CH_3_)_2_), 123.4 (s, –**CH**=C(CH_3_)_2_), 117.6 (s, –CH_2_–**CH**=(CH_3_)–CH_2_–), 70.9 (t, ^4^*J* = 4.3 Hz, –O–**CH**_**2**_–CH=,), 39.6 (s, =C(CH_3_)–**CH**_**2**_–), 26.3 (s, =C(CH_3_)–CH_2_–**CH**_**2**_–), 25.7 (s, =C(**CH**_**3**_)–CH_3_), 17.8 (s, =C(CH_3_)–**CH**_**3**_), 16.8 (s, =C(**CH**_**3**_)–CH_2_–).

FD-MS *m*/*z*: 303.15, 304.15.

Calc. *m*/*z*: 303.12, 304.13, 305.13.

##### 4.3.1.2 Synthesis of 26

PFP (1.092 g, 6.46 mmol, 1.3 eq.) was placed in a tomato paste can containing aluminum beads, along with Cs_2_CO_3_ (2.418 g, 7.42 mmol, 1.5 eq.), and myrtenol (0.740 g, 4.86 mmol, 1.0 eq.). Quantitative conversion was shown by ^19^F NMR after 15 minutes of agitation. The product was extracted with 3 × 50 mL of chloroform, vacuum filtered through alumina and a cotton plug in a glass funnel, and concentrated to a golden yellow oil (92% isolated yield).

^19^F NMR (CDCl_3_, 376 MHz, CCl_3_F) *δ*: −91.6 (m, 2F 2,6-position C_5_F_4_N), −158.3 (m, 2F 3,5-position C_5_F_4_N).

^1^H NMR (CDCl_3_, 400 MHz) *δ*: 5.72 (s, 1H, –C=**CH**–), 4.86 (dd, ^4^*J* = 11.90 Hz, ^5^*J* = 19.99 Hz, 2H –O–**CH**_**2**_–), 2.41 (dt, ^3^*J* = 5.59 Hz, ^4^*J* = 8.80 Hz, 1H, –O–C–**CH**–C(CH_3_)_2_–), 2.28 (m, 2H, =CH–**CH**_**2**_–CH), 2.10 (s, 3H, –**CH**–CH_2_–CH= and –CH–**CH**_2_–CH–), 1.30 (s, –C(**CH**_**3**_)–CH_3_), 1.02 (d, ^3^*J* = 6.56 Hz, 1H, –CH–**CH**_2_–CH–), 0.75 (s, 3H, –C(CH_3_)–**CH**_**3**_).

^13^C NMR (CDCl_3_, 100 MHz) *δ*: 147.2 (m, 4-position C_5_F_4_N), 144.3 (dm, ^1^*J* = 242 Hz, 3,5-position C_5_F_4_N), 142.7 (s, –O–CH_2_–**C**=), 135.5 (dm, ^1^*J* = 257 Hz, 2,6-position C_5_F_4_N), 124.9 (s, –C=**CH**–CH_2_), 77.0 (t, ^4^*J* = 4.40, –O–**CH**_**2**_–), 43.3 (s, –CH=C–**CH**–), 40.7 (s, –C(CH_3_)_2_–**CH**–CH_2_–CH=), 38.2 (s, –CH–**C**(CH_3_)_2_–CH–), 31.6 (s, =CH–**CH**_**2**_–CH–), 31.5 (s, =C–CH–**CH**_**2**_–CH–), 26.1 (s, –C(**CH**_**3**_)_2_–), 21.0 (s, –C(**CH**_**3**_)_2_–).

FD-MS *m*/*z*: 301.13, 302.13, 303.13.

Calc. *m*/*z*: 301.11, 302.11, 303.12.

##### 4.3.1.3 Synthesis of 27

PFP (1.101 g, 6.51 mmol, 1.3 eq.) was placed in a tomato paste can containing aluminum beads, along with Cs_2_CO_3_ (2.542 g, 7.80 mmol, 1.5 eq.), and (−)-carveol (0.774 g, 5.08 mmol, 1 eq.). Quantitative conversion was shown by ^19^F NMR after 30 minutes of agitation. The product was extracted with 3 × 50 mL of chloroform, vacuum filtered through alumina and a cotton plug in a glass funnel to yield a grey oil. This oil was filtered over silica and concentrated to a golden yellow oil (77% isolated yield).

^19^F NMR (CDCl_3_, 376 MHz, CCl_3_F) *δ*: −91.0 (m, 2F, 2,6-position C_5_F_4_N), −157.7 (m, 2F, 3,5-position C_5_F_4_N).

^1^H NMR (CDCl_3_, 400 MHz) *δ*: 5.86 (m, 2H, –C(CH3)=**CH**–), 5.69 (m, 1H, –C(CH_3_)=**CH**–), 5.26 (s, 1H, –O–**CH**–), 4.97 (s, 2H, –O–**CH**–), 4.76 (s, 4H, –C=**CH**_**2**_), 4.73 (s, 2H, –C=**CH**_**2**_), 2.51 (t, ^3^*J* = 5.52 Hz, 2H, –CH–C(**CH**_**3**_)=CH_2_), 2.36–1.90 (m, 13H, –**CH**_**2**_–**C**(C(CH_3_)=CH_2_)–**CH**_**2**_), 1.86 (s, 6H, –C(**CH**_**3**_)=CH_2_), 1.82 (s, 3H, –C(**CH**_**3**_)=CH_2_), 1.73 (s, 9H, –O–CH–C(**CH**_**3**_)=).

^13^C NMR (CDCl_3_, 101 MHz) *δ*: 148.2 and 147.8 (s, –**C**(CH_3_)=CH_2_), 147.4 (m, 4-position C_5_F_4_N), 144.4 (dm, ^1^*J* = 242 Hz, 3,5-position C_5_F_4_N), 135.5 (dm, ^1^*J* = 257 Hz, 2,6-position C_5_F_4_N), 132.5 and 130.4 (s, –O–CH–**C**(CH_3_)=CH–), 129.6 and 127.3 (s, –CH–C(CH_3_)=**CH**–), 110.0 and 109.8 (s, –C(CH_3_)=**CH**_**2**_), 84.8 (t, ^4^*J* = 3.43 Hz, –O–**CH**–), 82.8 (t, ^4^*J* = 4.10 Hz, –O–**CH**–), 40.5 and 35.2 (s, –CH_2_–**CH**(C(CH_3_)=CH_2_)–CH_2_–), 34.8 and 33.8 (s, –**CH**_**2**_–CH(C(CH_3_)=CH_2_)–CH_2_–), 31.0 and 30.9 (s, –CH_2_–CH(C(CH_3_)=CH_2_)–**CH**_**2**_–), 20.8 (s, –O–CH–C(**CH**_**3**_)=CH–), 20.4 and 18.3 (s, –C(**CH**_**3**_)=CH_2_).

FD-MS *m*/*z*: 301.14, 302.15.

Calc. m/*z* 301.11, 302.11, 303.12.

##### 4.3.1.4 Synthesis of 28

PFP (1.084 g, 6.41 mmol, 1.0 eq.) was placed in a tomato paste can containing aluminum beads, along with Cs_2_CO_3_ (2.346 g, 7.20 mmol, 1.1 eq.), and eugenol (1.158 g, 7.05 mmol, 1.1 eq.). Quantitative conversion was shown by ^19^F NMR after 5 minutes of agitation. The product was extracted with 3 × 50 mL of chloroform, vacuum filtered through alumina and a cotton plug in a glass funnel and concentrated to a golden yellow oil (≥99% isolated yield).

^19^F-NMR (CDCl_3_, 376 MHz, CCl_3_F) *δ*: −91.0 (m, 2,6 position C_5_F_4_N, 2F), −158.3 (m, 3,5 position C_5_F_4_N, 2F).

^1^H-NMR (CDCl_3_, 400 MHz) *δ*: 7.08 (d, ^3^*J* = 8.12 Hz, 1H, –O–Ar (position 5)–), 6.80 (d, ^4^*J* = 1.36 Hz, 1H, –O–Ar (position 3)–), 6.77 (dd, ^3^*J* = 8.10 Hz, ^4^*J* = 2.01 Hz, 1H, –O–Ar (position 6)–), 5.97 (m, 1H, –CH_2_–**CH**=CH_2_), 5.10 (m, 2H, –CH=**CH**_**2**_), 3.79 (s, 3H, –O–CH_**3**_), 3.40 (d, ^3^*J* = 6.56 Hz, 2H, –Ar–CH_2_–CH=).

^13^C-NMR (CDCl_3_, 100 MHz) *δ*: 150.1 (s, –O–Ar(1 position)–O–CH_3_), 146.2 (m, 4-position C_5_F_4_N), 144.0 (dm, ^1^*J* = 242 Hz, 3,5-position C_5_F_4_N), 142.6 (s, –O–Ar (2 position)–O–CH_3_), 139.1 (s, –CH_2_–**CH**=CH_2_), 136.9 (s, –O–Ar (4 position)–O–CH_3_), 135.1 (dm, ^1^*J* = 260 Hz, 2,6-position C_5_F_4_N), 120.9 (s, –O–Ar (6 position)–O–CH_3_), 119.6 (s, –O–Ar (5 position)–O–CH_3_), 116.5 (s, –CH=**CH**_**2**_), 113.0 (s, –O–Ar (3 position)–O–CH_3_), 56.0 (s, –O–**CH**_**3**_), 40.0 (s, –Ar–**CH**_**2**_–CH=CH_2_).

GC-EI/MS *m*/*z*: 313.1 (100%), 314.1 (15.4%), 315.1 (1.6%).

Calc. *m*/*z*: 313.07 (100%), 314.08 (16.4%), 315.08 (1.7%).

#### 4.3.2 General procedure for inverse vulcanization of natural product monomers with S_8_

Polymers were prepared using 25% by weight of the desired monomer and 75% by weight sulfur powder. The solutions were heated while stirring to 160 °C under atmospheric conditions until solid. The bulk material underwent TGA and DSC analysis without further purification.

##### 4.3.2.1 Inverse vulcanization of 25 with S_8_

0.528 g of 25 (25% wt.) and 1.543 g of sulfur powder (75% wt.) were combined and left to stir at 160 °C for 71 hours, upon which a solid was formed and the product was removed from heat and cooled to form a dark brown solid (1.683 g isolated yield).

##### 4.3.2.2 Inverse vulcanization of 26 with S_8_

0.540 g of 26 (27% wt) were added to 1.425 g of sulfur powder (73% wt). The solution was left to stir at 160 °C until the solution solidified (71 hours) to form a dark brown solid (1.161 g isolated yield).

##### 4.3.2.3 Inverse vulcanization of 27 with S_8_

0.532 g of 27 perfluoropyridine (26% wt), and 1.487 g of sulfur powder (74% wt) were combined, and allowed to stir until a solid was formed after 144 hours. The polymer formed was a dark brown solid (1.109 g isolated yield).

##### 4.3.2.4 Inverse vulcanization of 28 with S_8_

0.512 g of 28 (26% wt) and 1.480 g of sulfur powder (74% wt) were left to stir at 160 °C for 1 week. A viscous red solution was formed that solidified to a red-brown solid upon cooling to room temperature (1.459 g isolated yield).

#### 4.3.3 General procedure for inverse vulcanization of natural product monomers with S_2_Cl_2_

The prepolymer (1 eq.) was introduced into a clean dram vial with stirring and freshly distilled S_2_Cl_2_ (1 eq.) was added. The mixture was stirred under air or a N_2_ atmosphere, left to cure at 20 °C or 70 °C overnight for 20–24 hours. The mixture was precipitated into methanol, aged until solid, and filtered to afford the product as a powder.

##### 4.3.3.1 Inverse vulcanization of 25 with S_2_Cl_2_ at room temperature

Sulfur monochloride (0.394 g, 2.9 mmol, 1.4 eq.) was added to 25 (0.655 g, 2.2 mmol, 1 eq.) with stirring at room temperature for 24 hours. A dark, viscous oil was formed. This was dissolved in DCM and precipitated in methanol. The purified polymer was a solid and gave a final yield of 0.192 g, (20%).

##### 4.3.3.2 Inverse vulcanization of 25 with S_2_Cl_2_ in THF at 65 °C

Sulfur monochloride (0.13 mL, 0.220 g, 1.63 mmol, 1.4 eq.) was added to 25 (0.542 g, 1.79 mmol, 1.1 eq.) in 1 mL THF with stirring at 65 °C for 24 hours to form a yellow solution. This was dissolved in DCM and precipitated in methanol. The purified polymer was a light brown solid and gave a final yield of 0.322 g, (42%).

##### 4.3.3.3 Inverse vulcanization of 25 with S_2_Cl_2_ at 70 °C under N_2_

Sulfur monochloride (0.08 mL, 0.135 g, 1.00 mmol, 1.06 eq.) was added to 25 (0.286 g, 0.94 mmol, 1 eq.) with stirring at room temperature under a nitrogen blanket for 10 minutes, followed by heating at 70 °C for 22 hours to afford a dark solid. This was dissolved in THF and precipitated in methanol to give a brown powder with a final yield of 0.017 g (4%).

##### 4.3.3.4 Inverse vulcanization of 27 with S_2_Cl_2_ in THF at 65 °C

Sulfur monochloride (0.13 mL, 0.220 g, 1.63 mmol, 1.01 eq.), was added to 27 (0.486 g, 1.61 mmol, 1 eq.) in 1 mL of THF and heated at 65 °C for 22 hours to form an orange solution. The solution precipitated in excess methanol. The isolated polymer was a light brown solid, with a yield of 0.087 g (12%).

##### 4.3.3.5 Inverse vulcanization of 27 with S_2_Cl_2_ at 70 °C under N_2_

Sulfur monochloride (0.07 mL, 0.118 g, 0.876 mmol, 1 eq.), was added to 27 (0.275 g, 0.913 mmol, 1.04 eq.) and allowed to stir for ten minutes before heating to 70 °C for 22 hours, and a red solid was formed. This was dissolved in THF and precipitated in methanol. The isolated polymer was a light brown solid, with a yield of 0.146 g (37%).

## Abbreviations

ATR: attenuated total reflectance
DABCO: 1,4-diazabicyclo[2.2.2]octane
DSC: differential scanning calorimetry
EI: Electron impact
ESI: Electrospray ionization
FD: Field desorption
FTIR: Fourier transform infrared spectroscopy
GPC: gel permeation chromatography
MOF: Metal-organic framework
MS: mass spectrometry
MW: molecular weight
NMR: nuclear magnetic resonance
PDI: polydispersity index
PFP: pentafluoropyridine
PMMA: poly(methyl methacrylate)
PTFE: poly(tetrafluoroethylene)
SEC: size-exclusion chromatography
TCM: tin can milling
TGA: thermal gravimetric analysis
THF: tetrahydrofuran
TSE: twin screw extrusion
UV: ultraviolet

## Author contributions

The manuscript was written through contributions of all authors. All authors have given approval to the final version of the manuscript. The conceptual ideas for the manuscript came from Jason Pulfer and Chadron Friesen. The experimental work was performed by Jason Pulfer and Miriam Aldom. GPC, TGA, and DSC analysis was provided by Maxime Colpaert.

## Conflicts of interest

The authors declare no competing financial interest.

## Supplementary Material

RA-015-D5RA03019F-s001

## Data Availability

The data supporting this article have been included as part of the SI. Supplementary information is available: details on the synthesis and structural characterization of monomers by ^19^F NMR, ^1^H NMR, ^13^C NMR, GC-MS, and FD-MS. See DOI: https://doi.org/10.1039/d5ra03019f.
