# Supplementary material for: Tin can milling: low-tech mechanochemical synthesis of plant-based prepolymers incorporating perfluoropyridine
Source: RSC Adv. 2025 Sep 16;15(40):33151–61. doi: 10.1039/d5ra03019f (PMC12439250; doi:10.1039/d5ra03019f)
Supplement: RA-015-D5RA03019F-s001 [file RA-015-D5RA03019F-s001.pdf]

Supplementary Information (SI) for RSC Advances.
This journal is © The Royal Society of Chemistry 2025

Supporting Information for:

Tin Can Milling: Low Tech Mechanochemical Synthesis of Plant-Based Prepolymers Incorporating Perfluoropyridine

*Jason Pulfer,[1,2] Miriam Aldom,[1] Maxime Colpaert,[3] Tim Storr,[2] and Chadron M. Friesen[1]**

[1]Trinity Western University, 22500 University Drive, V2Y 1Y1, Langley, British Columbia, Canada

[2]Simon Fraser University, 8888 University Drive, V5A 1S6, Burnaby, British Columbia, Canada

[3]ICGM, Université de Montpellier, CNRS, École Nationale Supérieure de Chimie de Montpellier, Montpellier Cedex 5, 34293 Montpellier, France.

Table of Contents

***Materials*......5**
***Monomer Synthesis, Isolation, and Purification*......5**
- **General Reaction Procedure:......5**
- **Reaction of Perfluoropyridine with 1:......6**
- **Reaction of Perfluoropyridine with 2:......6**
- **Reaction of Perfluoropyridine with 3:......6**
- **Reaction of Perfluoropyridine with 4:......7**
- **Reaction of Perfluoropyridine with 5:......7**
- **Reaction of Perfluoropyridine with 6:......7**
- **Reaction of Perfluoropyridine with 7:......8**
- **Reaction of Perfluoropyridine with 8:......8**
- **Reaction of Perfluoropyridine with 9:......8**
- **Reaction of Perfluoropyridine with 10:......9**
- **Reaction of Perfluoropyridine with 11:......9**
- **Reaction of Perfluoropyridine with 12:......9**

Reaction of Perfluoropyridine with 13: 10
Reaction of Perfluoropyridine with 14: 10
Reaction of Perfluoropyridine with 15: 10
Reaction of Perfluoropyridine with 16: 11
Reaction of Perfluoropyridine with 17: 11
Reaction of Perfluoropyridine with 18: 11
Reaction of Perfluoropyridine with 19: 12
Reaction of Perfluoropyridine with 20: 12
Reaction of Perfluoropyridine with 21: 13
Reaction of Perfluoropyridine with 22: 13
Reaction of Perfluoropyridine with 23: 14
Reaction of Perfluoropyridine with 24: 15

Polymerizations and Characterization: 16

General Procedure for Inverse Vulcanization of monomers with S_8: 16
Inverse Vulcanization of 25 with S_8 16
Inverse Vulcanization of 26 with S_8 17
Inverse Vulcanization of 27 with S_8 17
Inverse Vulcanization of 28 with S_8 17

General Procedure for Inverse Vulcanization of monomers with S_2Cl_2: 17
Polymerization of 25 with S_2Cl_2 at room temperature: 17
Polymerization of 25 with S_2Cl_2 at 65 °C: 18
Polymerization of 25 with S_2Cl_2 at 70 °C under N_2: 18
Polymerization of 27 with S_2Cl_2 at 70 °C under air: 18
Polymerization of 27 with S_2Cl_2 at 70 °C under N_2: 18

Nuclear Magnetic Resonance (NMR) Spectroscopy: 19

.... **19**

Figure S2. ^{19}F NMR of 2. 20
Figure S3. ^{19}F NMR of 3. 21
Figure S4. ^{19}F NMR of 4. 22
Figure S5. ^{19}F NMR of 5. 23
Figure S6. ^{19}F NMR of 6. 24
Figure S7. ^{19}F NMR of 7. 25
Figure S8. ^{19}F NMR of 8. 26
Figure S9. ^{19}F NMR of 9. 27
Figure S10. ^{19}F NMR of 10. 28
Figure S11. ^{19}F NMR of 11. 29
Figure S12. ^{19}F NMR of 12. 30
Figure S13. ^{19}F NMR of 13. 31

Figure S14. ^{19}F NMR of 14. 32

Figure S15. ^{19}F NMR of 15. 33

Figure S16. ^{19}F NMR of 16. 34

Figure S17. ^{19}F NMR of 17. 35

Figure S18. ^{19}F NMR of 18. 36

Figure S19. ^{19}F NMR of 19. 37

Figure S20. ^{19}F NMR of 20 38

Figure S21. ^{19}F NMR of 25. 39

Figure S22. ^{1}H NMR of 25. 40

Figure S23. ^{13}C NMR of 25. 41

Figure S24. ^{19}F NMR of 26. 42

Figure S25. ^{1}H NMR of 26. 43

Figure S26. ^{13}C NMR of 26. 44

Figure S27. ^{19}F NMR of 27. 45

Figure S28. ^{1}H NMR of 27. 46

Figure S29. ^{13}C NMR of 27. 47

Figure S30. ^{19}F NMR of 28. 48

Figure S31. ^{1}H NMR of 28. 49

Figure S32. ^{13}C NMR of 28. 50

ATR-FTIR ***51***

Prepolymers: **51**

Figure S33: ATR-FTIR of **25**. 51
Figure S34: ATR-FTIR of **26**. 52
Figure S35: ATR-FTIR of **27**. 53
Figure S36: ATR-FTIR of **28**. 54

Polymers: **55**

Figure S37: ATR-FTIR of **25-S_2Cl_2**. 55
Figure S38: ATR-FTIR of **27-S_2Cl_2**. 56
Figure S39: ATR-FTIR of **25-S_8**. 57
Figure S40: ATR-FTIR of **26-S_8**. 58
Figure S41: ATR-FTIR of **27-S_8**. 59
Figure S42: ATR-FTIR of **28-S_8**. 60

Mass Spectroscopy: ***61***

Figure S43: FD-MS spectrum of 25. 61

Figure S44: FD-MS spectrum of 26. 62

Figure S45: FD-MS spectrum of 27. 63

Figure S46: GC EI-MS spectrum of 28. 64

Thermal Gravimetric Analysis (TGA): ***64***

Figure S47: TGA under air of 25 inverse vulcanized with S_2Cl_2 at room temperature.................64

Figure S48: TGA under nitrogen of 25 inverse vulcanized with S_2Cl_2 at room temperature. 65

Figure S49: TGA under air of 25 inverse vulcanized with S_2Cl_2 at 65 °C..65

Figure S50: TGA under nitrogen of 25 inverse vulcanized with S_2Cl_2 at 65 °C. 66

Figure S51: TGA under air of 25 inverse vulcanized with S_2Cl_2 at 70 °C in nitrogen atmosphere. 66

Figure S52: TGA under nitrogen of 25 inverse vulcanized with S_2Cl_2 at 70 °C in nitrogen atmosphere. 67

Figure S53: TGA under air of 27 inverse vulcanized with S_2Cl_2 at 70 °C..67

Figure S54: TGA under nitrogen of 27 inverse vulcanized with S_2Cl_2 at 70 °C. 68

Figure S55: TGA under air of 27 inverse vulcanized with S_2Cl_2 at 70 °C under nitrogen. 68

Figure S56: TGA under nitrogen of 27 inverse vulcanized with S_2Cl_2 at 70 °C under nitrogen. ..69

Figure S57: TGA under air of 25 inverse vulcanized with S_8..69

Figure S58: TGA under nitrogen of 25 inverse vulcanized with S_8. 70

Figure S59: TGA under air of 26 inverse vulcanized with S_8..70

Figure S60: TGA under nitrogen of 26 inverse vulcanized with S_8. 71

Figure S61: TGA under air of 27 inverse vulcanized with S_8..71

Figure S62: TGA under nitrogen of 27 inverse vulcanized with S_8. 72

Figure S63: TGA under air of 28 inverse vulcanized with S_8..72

Figure S64: TGA under nitrogen of 28 inverse vulcanized with S_8. 73

Differential Scanning Calorimetry (DSC):...73

Figure S65: DSC of 25 inverse vulcanized with S_2Cl_2 at 20 °C. 73

Figure S66: DSC of 25 inverse vulcanized with S_2Cl_2 at 65 °C. 74

Figure S67: DSC of 25 inverse vulcanized with S_2Cl_2 at 70 °C under nitrogen. 75

Figure S68: DSC of 27 inverse vulcanized with S_2Cl_2 at 70 °C...76

Figure S69: DSC of 27 inverse vulcanized with S_2Cl_2 at 70 °C under nitrogen. 77

Figure S70: DSC of 25 inverse vulcanized with S_8. 78

Figure S71: DSC of 26 inverse vulcanized with S_8. 79

Figure S72: DSC of 27 inverse vulcanized with S_8. 79

Figure S73: DSC of 28 inverse vulcanized with S_8. 80

Gel Permeation Chromatography:...81

Figure S74: GPC trace of 25 inverse vulcanized with S_2Cl_2 at room temperature. 81

Figure S75: GPC trace of 25 inverse vulcanized with S_2Cl_2 at 65 °C. 81

Figure S76: GPC trace of 25 inverse vulcanized with S_2Cl_2 at 70 °C under nitrogen.....................82

Figure S77: GPC trace of 27 inverse vulcanized with S_2Cl_2 at 70 °C. 82

Figure S78: GPC trace of 27 inverse vulcanized with S_2Cl_2 at 70 °C under nitrogen....................83

Materials

Chemicals were used as received unless otherwise noted. Geraniol (≥ 97%), eugenol (99%), (-)-carveol (mixture of isomers, 97%), myrtenol (≥ 95%), pyrrolidine (≥99.0%), pyridine (99.8%), methanol (≥99.6%), isopropanol (≥99.5%), 1,4-diazabicyclo[2.2.2]octane (DABCO) (≥99%), imidazole (≥99.5%), 2-pyrrolidinone (99%), 4-bromophenol (99%), 4-hydroxybenzaldehyde (98%), acrylic acid (≥99.0), glycidol (96%), 2-hydroxyethylmethacrylate (97%), hexanes (mixture of isomers, ≥98.5%), diethyl ether (≥99.0%), ethyl acetate (≥99.5%), chloroform (≥99.8%), dichloromethane (≥99.8%), tetrahydrofuran (≥99.0%), catechol (≥99%), alumina (80-200 mesh, chromatographic grade) and silica (70-230 mesh, 60Å, for column chromatography) all came from Sigma Aldrich. Sulfur monochloride (98%) was also obtained from Sigma Aldrich, it was distilled giving an orange liquid before use. Perfluoropyridine (PFP) (99%), cesium carbonate (99.9%), 3-cyclopenten-1-ol (98%) and diethylene glycol monovinyl ether (98%) were obtained from AK Scientific. Triphenylphosphine (powder, 99%) and sulfur (powder, -325 mesh, 99.5%) came from Alfa Aesar. *Tert*-Butanol was obtained from Fischer Scientific. Butylamine (98%) from EM Scientific; Aniline (99%) from Anachemia.

Monomer Synthesis, Isolation, and Purification

General Reaction Procedure:

For production of mono-substituted pentafluoropyridine in the 4-position mechanochemically, ~95 g of Lab Armor™ aluminum beads were placed in a Hunt's tomato paste can, along with 1.2 eq (2.346 g, 7.2 mmol) Cs_2CO_3, 1 eq PFP (1.01 g, 0.622mL, 6 mmol), and 1.2

eq (7.2 mmol) of nucleophile (e.g. 1.11 g, 1.25 mL of geraniol). The reactor was then agitated using a Burrell Wrist Action Shaker and monitored using ^{19}F NMR at the 5-, 15-, 30-, and 60-minute mark.

Reaction of Perfluoropyridine with 1:

PFP (1.097 g, 6.49 mmol, 1eq.), Cs_2CO_3 (2.390 g, 7.34 mmol, 1.1 eq.), and methanol (0.271 g, 8.46 mmol, 1.3 eq.) were combined in a tomato paste can with aluminum beads. 5 minutes of agitation showed 91% conversion to mono-substituted PFP, with 9% di-substituted PFP monitored by ^{19}F NMR with Freon-11 as an internal standard.

^{19}F NMR ($CDCl_3$, 376 MHz, CCl_3F) δ: -91.2 (m, 2,6 position C_5F_4N, 2F), -94.4 (dd, di 5 position C_5F_3N, ^3J = 25 Hz, ^4J = 23 Hz, 1F), -160.4 (m, 3,5 position C_5F_4N, 2F), -160.4 (dm, di 6 position C_5F_3N , ^3J = 25 Hz, 1F), - 168.1 (dm, di 3 position C_5F_3N, ^4J = 23 Hz, 1F).

Reaction of Perfluoropyridine with 2:

PFP (1.117 g, 6.61 mmol, 1 eq.), Cs_2CO_3 (2.681 g, 8.23 mmol, 1.2 eq.), and isopropanol (0.446 g, 7.42 mmol, 1.1 eq.) were combined in a tomato paste can with aluminum beads. 5 minutes of agitation shows quantitative conversion to mono-substituted PFP monitored by ^{19}F NMR with Freon-11 as an internal standard.

^{19}F NMR ($CDCl_3$, 376 MHz, CCl_3F) δ -91.5 (m, 2,6 position C_5F_4N, 2F), -158.9 (m, 3,5 position C_5F_4N, 2F).

Reaction of Perfluoropyridine with 3:

PFP (1.089 g, 6.44 mmol, 1 eq.), Cs_2CO_3 (2.31 9 g, 7.12 mmol, 1.1 eq.), and t-butanol (0.569 g, 7.68 mmol, 1.2 eq.) were combined in a tomato paste can with aluminum beads. 30

minutes of agitation shows 0% conversion by ^{19}F NMR with Freon-11 as an internal standard in $CDCl_3$.

^{19}F NMR ($CDCl_3$, 376 MHz, CCl_3F) δ -86.5 (s, 2,6 position C_5F_5N, 2F), -132.6 (m, 4 position C_5F_5N), -160.5 (m, 3,5 position C_5F_5N, 2F).

Reaction of Perfluoropyridine with 4:

PFP (1.154 g, 6.83 mmol, 1eq.), Cs_2CO_3 (2.650 g, 8.13 mmol, 1.2 eq.), and glycidol (0.621 g, 8.38 mmol, 1.2 eq.) were combined in a tomato paste can with aluminum beads. 5 minutes of agitation showed quantitative conversion to mono-substituted PFP monitored by ^{19}F NMR with Freon-11 as an internal standard.

^{19}F NMR ($CDCl_3$, 376 MHz, CCl_3F) δ -90.6 (m, 2,6 position C_5F_4N, 2F), -158.9 (m, 3,5 position C_5F_4N, 2F).

Reaction of Perfluoropyridine with 5:

PFP (1.150 g, 6.80 mmol, 1 eq.), Cs_2CO_3 (2.410 g, 7.40 mmol, 1.1 eq.), and linalool (1.193 g, 7.73 mmol, 1.1 eq.) were combined in a tomato paste can with aluminum beads. 60 minutes of agitation showed 67% conversion to mono-substituted PFP, with 33% unreacted PFP. This was monitored by ^{19}F NMR with Freon-11 as an internal standard.

^{19}F NMR ($CDCl_3$, 376 MHz, CCl_3F) δ -86.5 (s, unreacted C_5F_5N 2,6 position, 1F), -91.0 (m, 2,6 position C_5F_4N, 2F), -132.6 (m, unreacted C_5F_5N 4 position, 0.6F), -158.5 (m, 3,5 position C_5F_4N, 2F), -160.7 (m, unreacted C_5F_5N 3,5 position, 1F).

Reaction of Perfluoropyridine with 6:

PFP (1.116 g, 6.60 mmol, 1 eq.), Cs_2CO_3 (2.399 g, 7.36 mmol, 1.1 eq.), and 3-cyclopenten-1-ol (0.556 g, 6.61 mmol, 1 eq.) were combined in a tomato paste can with aluminum beads. 15

minutes of agitation showed 95% conversion to mono-substituted PFP monitored by ^{19}F NMR with Freon-11 as an internal standard.

^{19}F NMR ($CDCl_3$, 376 MHz, CCl_3F) δ -91.6 (m, 2,6 position C_5F_4N, 2F), -94.3 (t, unreacted C_5F_5N position 2 and 6, ^3J = 24 Hz, 0.06F), -139.4 (dt, unreacted C_5F_5N, ^3J = 18 Hz, ^4J= 12 Hz, 0.03F), -159.5 (m, 3,5 position C_5F_4N, 2F), -167.4 (d, unreacted C_5F_5N position 3 and 5, ^3J=23Hz, 0.08F).

Reaction of Perfluoropyridine with 7:

PFP (1.154 g, 6.83 mmol, 1eq.), Cs_2CO_3 (2.492 g, 7.65 mmol, 1.1 eq.), and diethylene glycol monovinyl ether (1.062 g, 8.04 mmol, 1.2 eq.) were combined in a tomato paste can with aluminum beads. 5 minutes of agitation showed quantitative conversion to mono-substituted PFP monitored by ^{19}F NMR with Freon-11 as an internal standard.

^{19}F NMR ($CDCl_3$, 376 MHz, CCl_3F) δ -91.2 (m, 2,6 position C_5F_4N, 2F), -159.0 (m, 3,5 position C_5F_4N, 2F).

Reaction of Perfluoropyridine with 8:

PFP (1.101 g, 6.51 mmol, 1eq.), Cs_2CO_3 (2.397 g, 7.36 mmol, 1.1 eq.), and 4-hydroxybenzaldehyde (0.854 g, 6.99 mmol, 1.1 eq.) were combined in a tomato paste can with aluminum beads. 30 minutes of agitation showed 67% conversion to mono-substituted PFP, with 33% unreacted PFP monitored by ^{19}F NMR with Freon-11 as an internal standard.

^{19}F NMR ($CDCl_3$, 376 MHz, CCl_3F) δ -86.5 (s, unreacted C_5F_5N 2, 6 position, 1F) -87.8 (m, 2,6 position C_5F_4N, 2F), -132. 6 (m, unreacted C_5F_5N 4 position, 0.5F), -153.8 (m, 3,5 position C_5F_4N, 2F), -160.7 (m, unreacted C_5F_5N 2, 6 position, 1F).

Reaction of Perfluoropyridine with 9:

PFP (1.085 g, 6.42 mmol, 1 eq.), Cs_2CO_3 (2.317 g, 7.11 mmol, 1.1 eq.), and 4-bromophenol (1.189 g, 6.87 mmol, 1.1 eq.) were combined in a tomato paste can with aluminum beads. 15 minutes of agitation showed 96% conversion to mono-substituted PFP, with 4% unreacted PFP monitored by ^{19}F NMR with Freon-11 as an internal standard.

^{19}F NMR ($CDCl_3$, 376 MHz, CCl_3F) δ: -88.5 (m, 2,6 position C_5F_4N, 2F), -154.6 (m, 3,5 position C_5F_4N, 2F).

Reaction of Perfluoropyridine with 10:

PFP (1.108 g, 6.55 mmol, 2eq.), Cs_2CO_3 (2.322 g, 7.13 mmol, 2.2 eq.), and catechol (0.353 g, 3.21 mmol, 1 eq.) were combined in a tomato paste can with aluminum beads. 30 minutes of agitation showed quantitative conversion to mono-substituted PFP monitored by ^{19}F NMR with Freon-11 as an internal standard.

^{19}F NMR ($CDCl_3$, 376 MHz, CCl_3F) δ: -88.5 (m, 2,6 position C_5F_4N, 4F), -156.1 (m, 3,5 position C_5F_4N, 4F).

Reaction of Perfluoropyridine with 11:

PFP (1.135 g, 6.71 mmol, 1 eq.), Cs_2CO_3 (2.346 g, 7.20 mmol, 1.1 eq.), and 2-hydroxyethyl methacrylate (1.029 g, 7.91 mmol, 1.2 eq.) were combined in a tomato paste can with aluminum beads. 5 minutes of agitation showed quantitative conversion to mono-substituted PFP monitored by ^{19}F-NMR with Freon-11 as an internal standard.

^{19}F NMR ($CDCl_3$, 376 MHz, CCl_3F) δ: -90.6 (m, 2,6 position C_5F_4N, 2F), -159.1 (m, 3,5 position C_5F_4N, 2F).

Reaction of Perfluoropyridine with 12:

PFP (1.128 g, 6.67 mmol, 1 eq.), Cs_2CO_3 (2.457 g, 7.54 mmol, 1.1 eq.), and acrylic acid (0.565 g, 7.84 mmol, 1.2 eq.) were combined in a tomato paste can with aluminum beads. 30 minutes of agitation showed quantitative conversion to mono-substituted PFP monitored by ^{19}F-NMR in D_2O with Freon-11 as an internal standard.

^{19}F NMR (D_2O, 376 MHz, CCl_3F) δ: -100.0 (m, 2,6 position C_5F_4N, 2F), -120.8 (s, F^-), -168.5 (m, 3,5 position C_5F_4N, 2F).

Reaction of Perfluoropyridine with 13:

PFP (1.125 g, 6.55 mmol, 1eq.), Cs_2CO_3 (2.603 g, 7.99 mmol, 1.2 eq.), and butylamine (0.516 g, 7.05 mmol, 1.1 eq.) were combined in a tomato paste can with aluminum beads. 5 minutes of agitation showed quantitative conversion to mono-substituted PFP monitored by ^{19}F-NMR with Freon-11 as an internal standard.

^{19}F NMR ($CDCl_3$, 376 MHz, CCl_3F) δ: -94.9 (m, 2,6 position C_5F_4N, 2F), -165.1 (m, 3,5 position C_5F_4N, 2F).

Reaction of Perfluoropyridine with 14:

PFP (1.134 g, 6.71 mmol, 1eq.), Cs_2CO_3 (2.271 g, 6.97 mmol, 1 eq.), and pyrrolidine (0.563 g, 7.92 mmol, 1.2 eq.) were combined in a tomato paste can with aluminum beads. 5 minutes of agitation showed 95% conversion to mono-substituted PFP, with 5% di-substituted determined using ^{19}F-NMR with Freon-11 as an internal standard.

^{19}F NMR ($CDCl_3$, 376 MHz, CCl_3F) δ: -96.0 (m, 2,6 position C_5F_4N, 2F), -97.0 (dd, di 5 position C_5F_3N , ^3J = 24 Hz, ^4J = 25 Hz, 1F), -156.6 (dm, di 6 position C_5F_3N , ^3J = 23 Hz, 1F), -161.2 (m, 3,5 position C_5F_4N, 2F), - 171.2 (dm, di 3 position C_5F_3N, ^4J = 26 Hz, 1F).

Reaction of Perfluoropyridine with 15:

PFP (1.127 g, 6.67 mmol, 1 eq.), Cs_2CO_3 (2.426 g, 7.45 mmol, 1.1 eq.), and imidazole (0.474 g, 6.96 mmol, 1 eq.) were combined in a tomato paste can with aluminum beads. 5 minutes of agitation showed quantitative conversion to mono-substituted PFP monitored by ^{19}F-NMR with Freon-11 as an internal standard.

^{19}F NMR ($CDCl_3$, 376 MHz, CCl_3F) δ: -87.1 (m, 2,6 position C_5F_4N, 2F), -150.0 (m, 3,5 position C_5F_4N, 2F).

Reaction of Perfluoropyridine with 16:

PFP (1.087 g, 6.43 mmol, 1 eq.), Cs_2CO_3 (2.403 g, 7.38 mmol, 1.1 eq.), and aniline (0.671 g, 7.20 mmol, 1.1 eq.) were combined in a tomato paste can with aluminum beads. 30 minutes of agitation showed 4% conversion to mono-substituted PFP by ^{19}F-NMR with Freon-11 as an internal standard.

^{19}F NMR (376 MHz, $CDCl_3$) δ -86.07 (m, 2F), -88.36 (m, 0.06F), -92.43 (m, 0.03F), -132.11 (m, 1F), -147.63 (m, 0.06F), -155.58 (m, 0.04F), -160.25 (m, 2F).

Reaction of Perfluoropyridine with 17:

PFP (1.099 g, 6.50 mmol, 1 eq.), Cs_2CO_3 (2.321 g, 7.12 mmol, 1.1 eq.), and 2-pyrrolidinone (0.363 g, 7.47 mmol, 1.1 eq.) were combined in a tomato paste can with aluminum beads. 5 minutes of agitation showed quantitative conversion to mono-substituted PFP monitored by ^{19}F-NMR with Freon-11 as an internal standard.

^{19}F NMR ($CDCl_3$, 376 MHz, CCl_3F) δ: -90.0 (m, 2,6 position C_5F_4N, 2F), -144.1 (m, 3,5 position C_5F_4N, 2F).

Reaction of Perfluoropyridine with 18:

PFP (1.106 g, 6.54 mmol, 1eq.), Cs_2CO_3 (2.272 g, 6.97 mmol, 1.1 eq.), and pyridine (0.608 g, 7.69 mmol, 1.2 eq.) were combined in a tomato paste can with aluminum beads. 30 minutes of agitation showed quantitative conversion to mono-substituted PFP monitored in D_2O by ^{19}F-NMR with Freon-11 as an internal standard.

^{19}F NMR (D_2O, 376 MHz, CCl_3F) δ: -100.1 (m, 2,6 position C_5F_4N, 2F), -121.0 (s, F^-), -168.5 (m, 3,5 position C_5F_4N, 2F).

Reaction of Perfluoropyridine with 19:

PFP (1.105g, 6.54 mmol, 1 eq.), Cs_2CO_3 (2.321g, 7.12 mmol, 1.1 eq.), and 2,4-diazobicyclo[2.2.2]octane (0.399g, 3.56 mmol, 0.5 eq.) were combined in a tomato paste can with aluminum beads. 30 minutes of agitation showed 78% conversion to mono-substituted PFP in D-$_2$O monitored by ^{19}F-NMR with Freon-11 as an internal standard.

^{19}F NMR (D_2O, 376 MHz, CCl_3F) δ: -100.1 (m, 2,6 position C_5F_4N, 2F), -120.8 (s, F^-), -168.4 (m, 3,5 position C_5F_4N, 2F).

Reaction of Perfluoropyridine with 20:

PFP (1.097 g, 6.49 mmol, 1eq.), Cs_2CO_3 (2.305 g, 7.07 mmol, 1.1 eq.), and triphenylphosphine (1.866 g, 7.11 mmol, 1.1 eq.) were combined in a tomato paste can with aluminum beads. 15 minutes of agitation showed quantitative conversion to mono-substituted PFP monitored by ^{19}F-NMR with Freon-11 as an internal standard. A shift in the ^{31}P-NMR was observed relative to a 1% triphenylphosphine solution in $CDCl_3$ as an internal standard.

^{19}F NMR (D_2O, 376 MHz, CCl_3F) δ: -101.5 (m, 2,6 position C_5F_4N, 2F), -122.0 (s, F^-), -169.9 (m, 3,5 position C_5F_4N, 2F).

^{31}P NMR (D_2O, 162 MHz, 1% triphenyl phosphine) δ 2.14 (s, 1P).

Reaction of Perfluoropyridine with 21:

PFP (1.098 g, 6.50 mmol, 1.2 eq.) was placed in a tomato paste can containing aluminum beads, along with Cs_2CO_3 (2.390 g, 7.34 mmol, 1.3 eq.), and geraniol (0.846 g, 5.48 mmol, 1.0 eq.). Quantitative conversion was shown by ^{19}F NMR after 10 minutes of agitation. The product was extracted with 3x50 mL of chloroform, vacuum filtered through alumina and a cotton plug in a glass funnel, and concentrated to a golden yellow oil (88% isolated yield, compound **25**)

^{19}F NMR ($CDCl_3$, 376 MHz, CCl_3F) δ: -91.6 (m, 2F, 2,6-position C_5F_4N), -159.0 (m, 2F, 3,5-position C_5F_4N).

^{1}H NMR ($CDCl_3$, 400 MHz) δ: 5.47 (t, ^{3}J = 6.94Hz ,1H, -O-CH_2-**CH**=C-), 5.02 (s, 1H, -**CH**=$C(CH_3)_2$), 5.00 (s, 2H, -O-**CH_2**-CH=), 2.08 (s, 4H, -**CH_2CH_2**-CH=C-), 1.74 (s, 3H, -O-CH_2-CH=C-**CH_3**), 1.66 (s, 3H, -C-(**CH_3**)$_2$), 1.59 (s, 3H -C-(**CH_3**)$_2$).

^{13}C-NMR ($CDCl_3$, 100MHz) δ: 147.2 (m, 4-position C_5F_4N), 146.1 (s, -CH=**C**(CH_3)-CH_2-), 144.3 (dm, ^{1}J = 242 Hz, 3,5-position C_5F_4N), 135.5 (dm, ^{1}J = 256 Hz, 2,6-position C_5F_4N), 132.3 (s, -CH=**C**$(CH_3)_2$), 123.4 (s, -**CH**=$C(CH_3)_2$), 117.6 (s, -CH_2-**CH**=(CH_3)-CH_2-), 70.9 (t, ^{4}J = 4.3 Hz, -O-**CH_2**-CH=,), 39.6 (s, =C(CH_3)-**CH_2**-), 26.3 (s, =C(CH_3)-CH_2-**CH_2**-), 25.7 (s, =C(**CH_3**)-CH_3), 17.8 (s, =C(CH_3)-**CH_3**), 16.8 (s, =C(**CH_3**)-CH_2-).

FD-MS *m/z*: 303.15, 304.15.

Calc. *m/z*: 303.12, 304.13, 305.13.

Reaction of Perfluoropyridine with 22:

PFP (1.092 g, 6.46 mmol, 1.3 eq.) was placed in a tomato paste can containing aluminum beads, along with Cs_2CO_3 (2.418 g, 7.42 mmol, 1.5 eq.), and myrtenol (0.740 g, 4.86 mmol, 1.0

eq.). Quantitative conversion was shown by ^{19}F NMR after 15 minutes of agitation. The product was extracted with 3x50 mL of chloroform, vacuum filtered through alumina and a cotton plug in a glass funnel and concentrated to a golden yellow oil (92% isolated yield, compound **26**).

^{19}F NMR ($CDCl_3$, 376 MHz, CCl_3F) δ: -91.6 (m, 2F 2,6-position C_5F_4N), -158.3 (m, 2F 3,5-position C_5F_4N).

^{1}H NMR ($CDCl_3$, 400 MHz) δ: 5.72 (s, 1H, -C=**CH**-), 4.86 (dd, ^4J = 11.90 Hz, ^5J = 19.99 Hz, 2H -O-**CH_2**-), 2.41 (dt, ^3J = 5.59 Hz, ^4J = 8.80 Hz, 1H, -O-C-**CH**-$C(CH_3)_2$-), 2.28 (m, 2H, =CH-**CH_2**-CH), 2.10 (s, 3H, -**CH**-CH_2-CH= and -CH-**CH_2**-CH-), 1.30 (s, -C(**CH_3**)-CH_3), 1.02 (d, ^3J = 6.56 Hz, 1H, -CH-**CH_2**-CH-), 0.75 (s, 3H, -C(CH_3)-**CH_3**).

^{13}C NMR ($CDCl_3$, 100MHz) δ: 147.2 (m, 4-position C_5F_4N), 144.3 (dm, ^1J = 242 Hz, 3,5-position C_5F_4N), 142.7 (s, -O-CH_2-**C**=), 135.5 (dm, ^1J = 257 Hz, 2,6-position C_5F_4N), 124.9 (s, -C=**CH**-CH_2), 77.0 (t, ^4J = 4.40, -O-**CH_2**-), 43.3 (s, -CH=C-**CH**-), 40.7 (s, -$C(CH_3)_2$-**CH**-CH_2-CH=), 38.2 (s, -CH-**C**$(CH_3)_2$-CH-), 31.6 (s, =CH-**CH_2**-CH-), 31.5 (s, =C-CH-**CH_2**-CH-), 26.1 (s, -C(**CH_3**)$_2$-), 21.0 (s, -C(**CH_3**)$_2$-).

FD-MS *m/z*: 301.13, 302.13, 303.13.

Calc. *m/z*: 301.11, 302.11, 303.12.

Reaction of Perfluoropyridine with 23:

PFP (1.101 g, 6.51 mmol, 1.3 eq.) was placed in a tomato paste can containing aluminum beads, along with Cs_2CO_3 (2.542 g, 7.80 mmol, 1.5 eq.), and (-)-carveol (0.774 g, 5.08 mmol, 1 eq.). Quantitative conversion was shown by ^{19}F NMR after 30 minutes of agitation. The product was extracted with 3x50 mL of chloroform, vacuum filtered through alumina and a cotton plug in a glass funnel to yield a grey oil. This oil was filtered over silica and concentrated to a golden yellow oil (77 % isolated yield, compound **27**).

^{19}F NMR ($CDCl_3$, 376 MHz, CCl_3F) δ: -91.0 (m, 2F, 2,6-position C_5F_4N), -157.7 (m, 2F, 3,5-position C_5F_4N).

^{1}H NMR ($CDCl_3$, 400 MHz) δ: 5.86 (m, 2H, -C(CH3)=**CH**-), 5.69 (m, 1H, -C(CH3)=**CH**-), 5.26 (s, 1H, -O-**CH**-), 4.97 (s, 2H, -O-**CH** -), 4.76 (s, 4H, -C=**CH_2**), 4.73 (s, 2H, -C=**CH_2**), 2.51 (t, ^{3}J = 5.52 Hz, 2H, -CH-C(**CH_3**)=CH_2), 2.36-1.90 (m, 13H, **-CH_2-C**(C(CH_3)=CH_2)-**CH_2**), 1.86 (s, 6H, -C(**CH_3**)=CH_2), 1.82 (s, 3H, -C(**CH_3**)=CH_2), 1.73 (s, 9H, -O-CH-C(**CH_3**)=).

^{13}C NMR ($CDCl_3$, 101 MHz) δ: 148.2 and 147.8 (s, -**C**(CH_3)=CH_2), 147.4 (m, 4-position C_5F_4N), 144.4 (dm, ^{1}J = 242 Hz, 3,5-position C_5F_4N), 135.5 (dm, ^{1}J = 257 Hz, 2,6-position C_5F_4N), 132.5 and 130.4 (s, -O-CH-**C**(CH_3)=CH-), 129.6 and 127.3 (s, -CH-C(CH_3)=**CH**-), 110.0 and 109.8 (s, -C(CH_3)=**CH_2**), 84.8 (t, ^{4}J = 3.43 Hz, -O-**CH**-), 82.8 (t, ^{4}J = 4.10 Hz, -O-**CH**-), 40.5 and 35.2 (s, -CH_2-**CH**(C(CH_3)=CH_2)-CH_2-), 34.8 and 33.8 (s, -**CH_2**-CH(C(CH_3)=CH_2)-CH_2-), 31.0 and 30.9 (s, -CH_2-CH(C(CH_3)=CH_2)-**CH_2**-), 20.8 (s, -O-CH-C(**CH_3**)=CH-), 20.4 and 18.3 (s, -C(**CH_3**)=CH_2).

FD-MS m/z: 301.14, 302.15.

Calc. m/z: 301.11, 302.11, 303.12.

Reaction of Perfluoropyridine with 24:

PFP (1.084 g, 6.41 mmol, 1.0 eq.) was placed in a tomato paste can containing aluminum beads, along with Cs_2CO_3 (2.346 g, 7.20 mmol, 1.1 eq.), and eugenol (1.158 g, 7.05 mmol, 1.1 eq.). Quantitative conversion was shown by ^{19}F NMR after 5 minutes of agitation. The product was extracted with 3x50 mL of chloroform, vacuum filtered through alumina and a cotton plug in a glass funnel and concentrated to a golden yellow oil ($\geq$99% isolated yield, compound **28**).

^{19}F-NMR ($CDCl_3$, 376 MHz, CCl_3F) δ: -91.0 (m, 2,6 position C_5F_4N, 2F), -158.3 (m, 3,5 position C_5F_4N, 2F).

^{1}H-NMR ($CDCl_3$, 400 MHz) δ: 7.08 (d, ^3J = 8.12 Hz, 1H, -O-Ar (position 5)-), 6.80 (d, ^4J = 1.36 Hz, 1H, -O-Ar (position 3)-), 6.77 (dd, ^3J = 8.10 Hz, ^4J = 2.01 Hz, 1H, -O-Ar (position 6)-), 5.97 (m, 1H, -CH_2-**CH**=CH_2), 5.10 (m, 2H, -CH=**CH_2**), 3.79 (s, 3H, -O-CH_3), 3.40 (d, ^3J = 6.56 Hz, 2H, -Ar-CH_2-CH=).

^{13}C-NMR ($CDCl_3$, 100MHz) δ: 150.1 (s, -O-Ar(1 position)-O-CH_3), 146.2 (m, 4-position C_5F_4N), 144.0 (dm, ^1J = 242 Hz, 3,5-position C_5F_4N), 142.6 (s, -O-Ar(2 position)-O-CH_3), 139.1 (s, -CH_2-**CH**=CH_2), 136.9 (s, -O-Ar(4-position)-O-CH_3), 135.1 (dm, ^1J = 260 Hz, 2,6-position C_5F_4N), 120.9 (s, -O-Ar(6 position)-O-CH_3), 119.6 (s, -O-Ar(5 position)-O-CH_3), 116.5 (s, -CH=**CH_2**), 113.0 (s, -O-Ar(3 position) -O-CH_3), 56.0 (s, -O-**CH_3**), 40.0 (s, -Ar-**CH_2**-CH=CH_2).

GC-EI/MS *m/z*: 313.1 (100%), 314.1 (15.4%), 315.1 (1.6%).

Calc. *m/z*: 313.07 (100%), 314.08 (16.4%), 315.08 (1.7%).

Polymerizations and Characterization:

General Procedure for Inverse Vulcanization of monomers with S_8:

Polymers were prepared using 25% by weight of the desired monomer and 75% by weight sulfur powder. The solutions were heated to 160°C with stirring until solid.

Inverse Vulcanization of 25 with S_8

0.528 g of **25** (25% wt.) and 1.543g of sulfur powder (75% wt.) were combined and left to stir at 160 °C for 71 hours, upon which a solid was formed and the product was removed from heat and cooled to form a dark brown solid (**25-S_8**).

Inverse Vulcanization of 26 with S_8

0.540 g of **26** (27% wt.) were added to 1.425 g of sulfur powder (73% wt.). The solution was left to stir at 160 ˚C until the solution solidified (71 hours) to form a dark brown solid (**26-S_8**).

Inverse Vulcanization of 27 with S_8

0.532 g of **27** perfluoropyridine (26% wt.), and 1.487 g of sulfur powder (74% wt.) were combined, and allowed to stir at 160 °C until a solid was formed after 144 hours. The polymer formed was a dark brown solid (**27-S_8**).

Inverse Vulcanization of 28 with S_8

0.512 g of **28** (26% wt.) and 1.480 g of sulfur powder (74% wt.) were left to stir at 160 ˚C for 1 week. A viscous red solution was formed that solidified to a red-brown solid upon cooling to room temperature (**28-S_8**).

General Procedure for Inverse Vulcanization of monomers with S_2Cl_2:

The prepolymer (1 eq.) was introduced into a clean dram vial with stirring and freshly distilled S_2Cl_2 (1 eq.) was added. The mixture was stirred under N_2 or air atmosphere, and either left to cure at 20 °C or 70 °C overnight for 20-24 hours. The mixture was precipitated into methanol, aged until solid, and filtered to afford the product as a powder.

Polymerization of 25 with S_2Cl_2 at room temperature:

Sulfur monochloride (0.394 g, 2.9 mmol, 1.4 eq) was added to **25** (0.655 g, 2.2 mmol, 1 eq) with stirring at room temperature for 24 hours. A dark, viscous oil was formed. This was dissolved

in DCM and precipitated in methanol. The purified polymer was a solid and gave a final yield of 0.192g (20 %).

Polymerization of 25 with S_2Cl_2 at 65 °C:

Sulfur monochloride (0.13 mL, 0.220 g, 1.63 mmol, 1.4 eq) was added to **25** (0.542 g, 1.79 mmol, 1.1 eq) in 1 mL THF with stirring at 65 °C for 24 hours to form a yellow solution. This was dissolved in DCM and precipitated in methanol. The purified polymer was a light brown solid and gave a final yield of 0.322 g (42 %).

Polymerization of 25 with S_2Cl_2 at 70 °C under N_2:

Sulfur monochloride (0.08 mL, 0.135 g, 1.00 mmol, 1.06 eq) was added to **25** (0.286 g, 0.94 mmol, 1 eq) with stirring at room temperature under a nitrogen blanket for 10 minutes, followed by heating at 70 °C for 22 hours to afford a dark solid. This was dissolved in THF and precipitated in methanol to give a brown powder with a final yield of 0.017 g (4 %).

Polymerization of 27 with S_2Cl_2 at 70 °C under air:

Sulfur monochloride (0.13 mL, 0.220 g, 1.63 mmol, 1.01 eq), was added to **27** (0.486 g, 1.61 mmol, 1 eq) in 1 mL of THF and heated at 65 °C for 22 hours to form an orange solution. The solution precipitated in excess methanol. The isolated polymer was a light brown solid, with a yield of 0.087 g (12 %).

Polymerization of 27 with S_2Cl_2 at 70 °C under N_2:

Sulfur monochloride (0.07 mL, 0.118 g, 0.876 mmol, 1 eq), was added to **27** (0.275 g, 0.913 mmol, 1.04 eq) and allowed to stir for ten minutes before heating to 70 °C for 22 hours, and a red solid was formed. This was dissolved in THF and precipitated in methanol. The isolated polymer was a light brown solid, with a yield of 0.146 g (37 %).

Nuclear Magnetic Resonance (NMR) Spectroscopy:

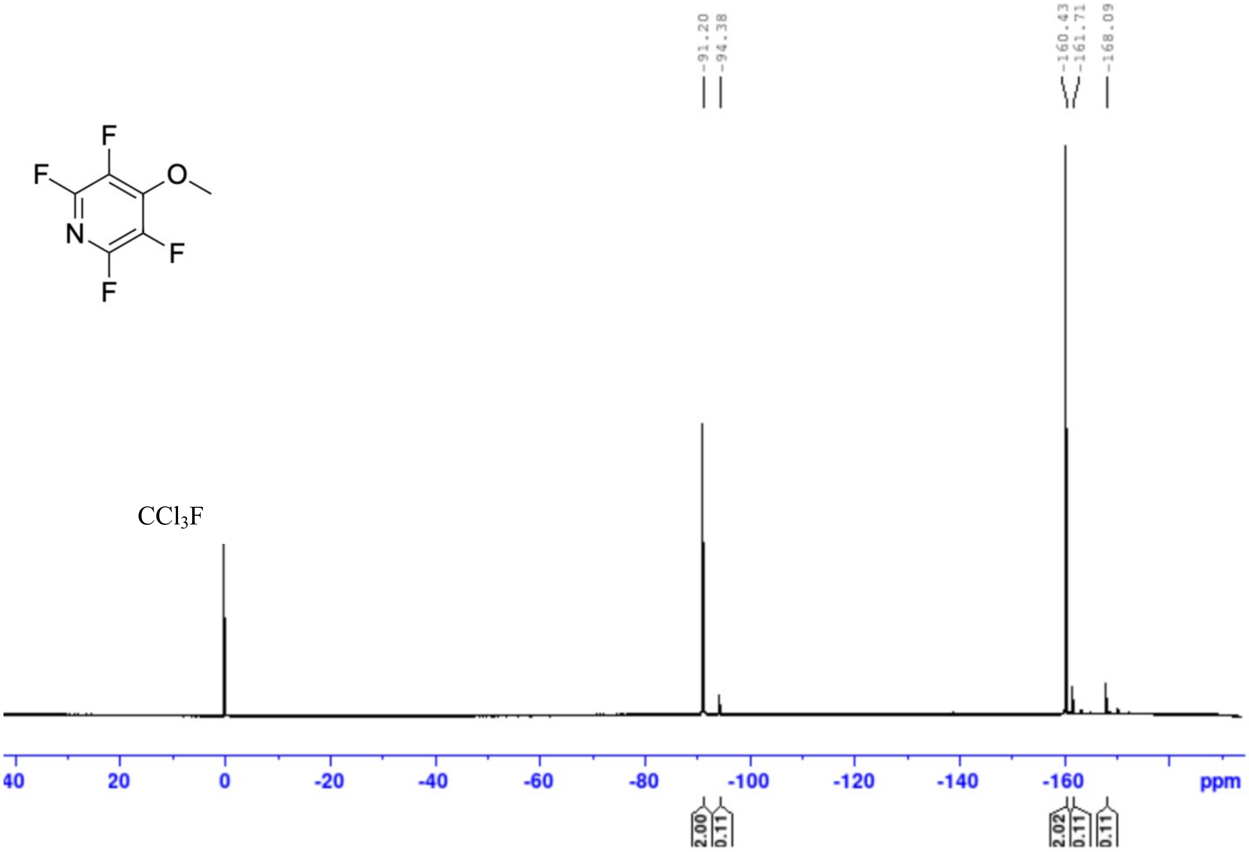

Figure S1. ^{19}F NMR of **1**.

^{19}F NMR ($CDCl_3$, 376 MHz, CCl_3F) δ -91.2 (m, 2,6 position C_5F_4N, 2F), -94.4 (dd, di 5 position C_5F_3N, ^3J = 25 Hz, ^4J = 23 Hz, 1F), -160.4 (m, 3,5 position C_5F_4N, 2F), -160.4 (dm, di 6 position C_5F_3N , ^3J = 25 Hz, 1F), - 168.1 (dm, di 3 position C_5F_3N, ^4J = 23 Hz, 1F).

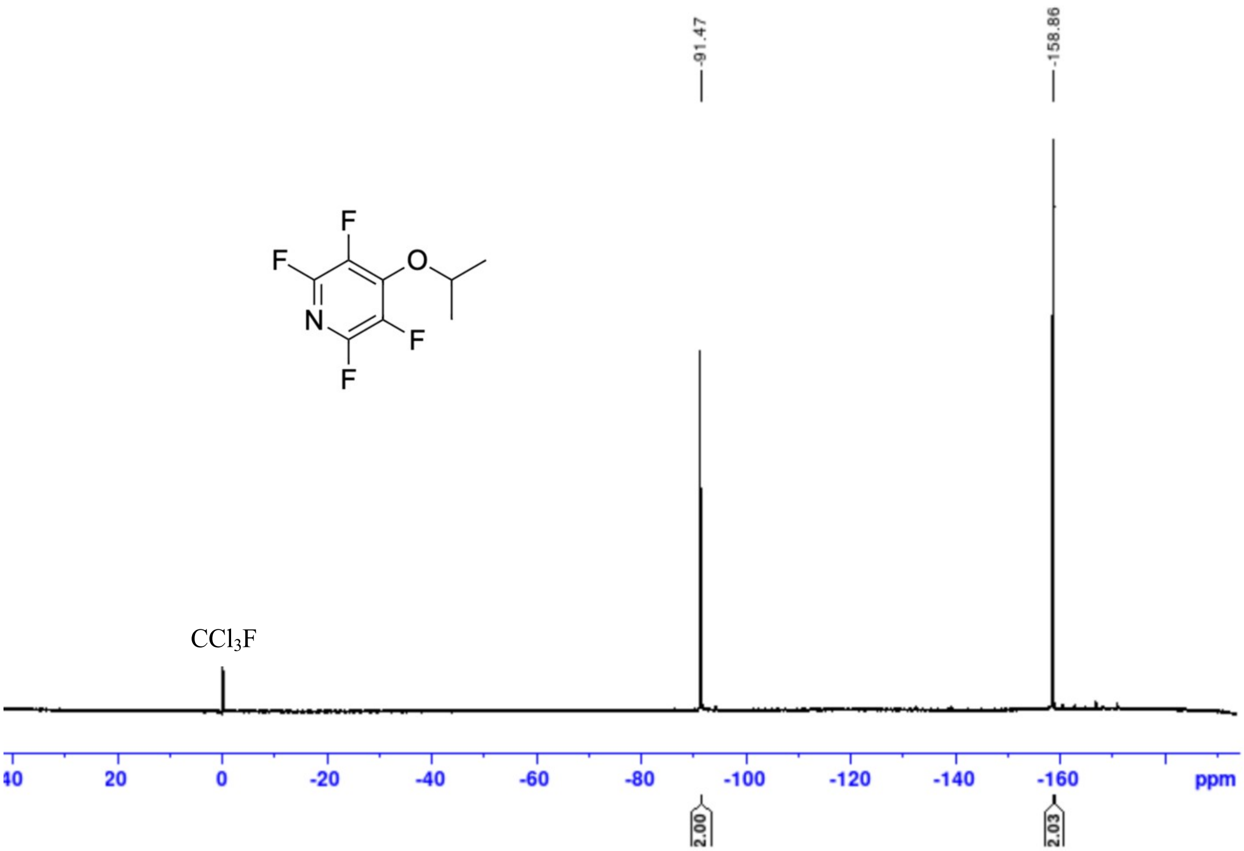

Figure S2. ^{19}F NMR of **2**.

^{19}F NMR ($CDCl_3$, 376 MHz, CCl_3F) δ -91.5 (m, 2,6 position C_5F_4N, 2F), -158.9 (m, 3,5 position C_5F_4N, 2F).

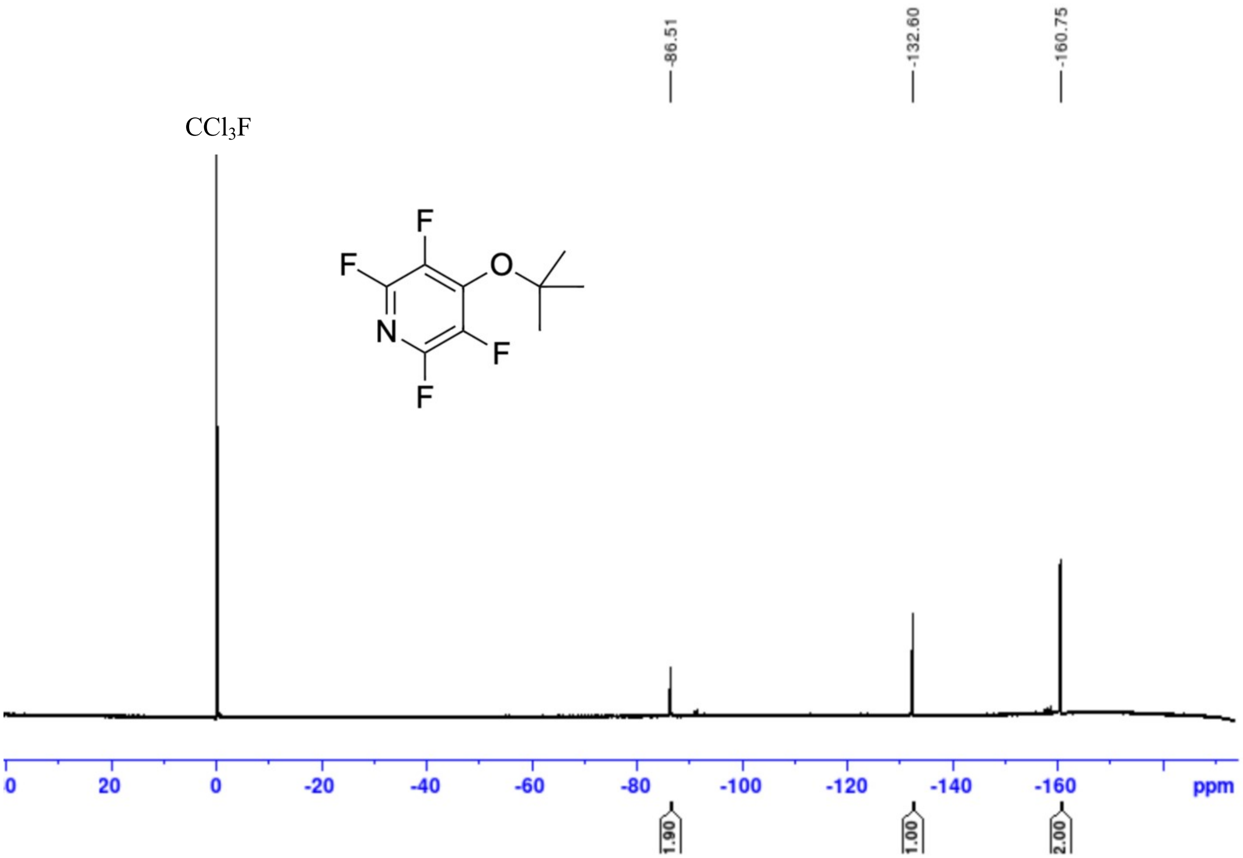

Figure S3. ^{19}F NMR of **3**.

^{19}F NMR ($CDCl_3$, 376 MHz, CCl_3F) δ -86.5 (s, 2,6 position C_5F_5N, 2F), -132.6 (m, 4 position C_5F_5N), -160.5 (m, 3,5 position C_5F_5N, 2F).

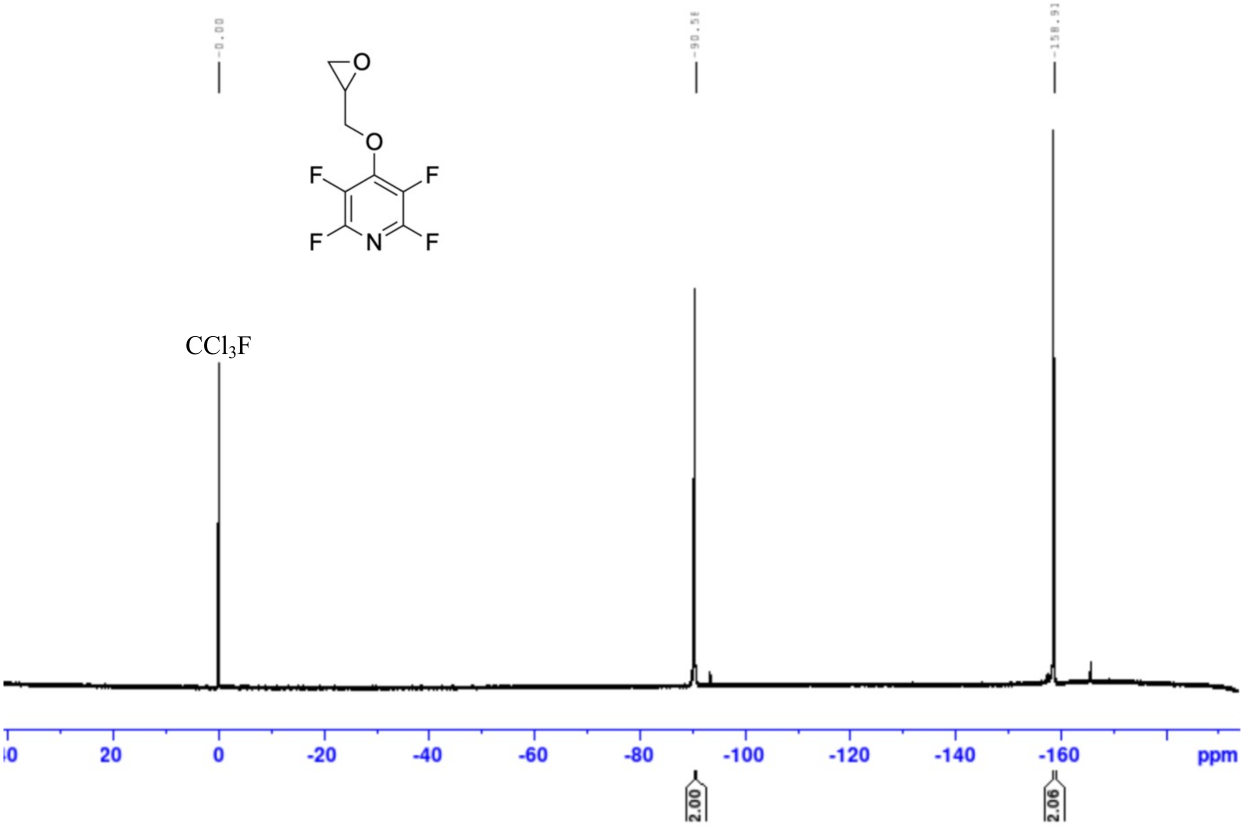

Figure S4. ^{19}F NMR of **4**.

^{19}F NMR ($CDCl_3$, 376 MHz, CCl_3F) δ -90.6 (m, 2,6 position C_5F_4N, 2F), -158.9 (m, 3,5 position C_5F_4N, 2F).

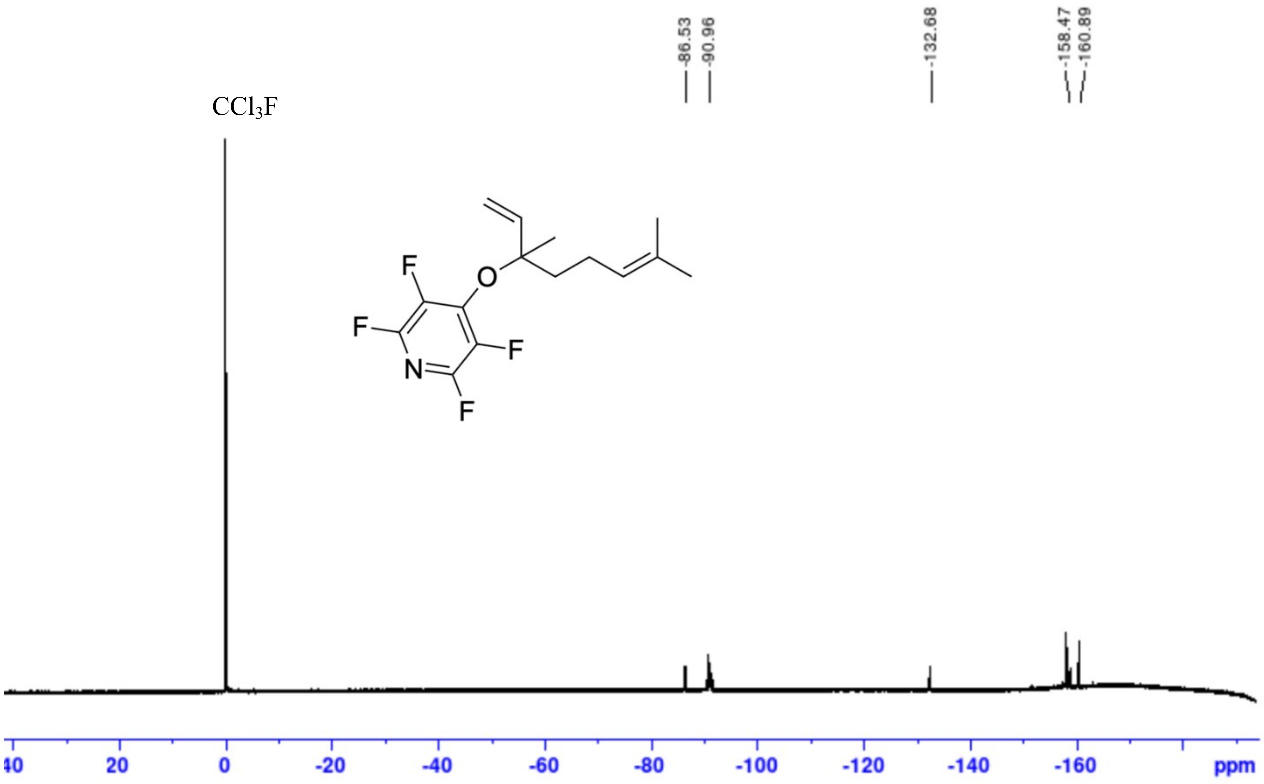

Figure S5. ^{19}F NMR of **5**.

^{19}F NMR ($CDCl_3$, 376 MHz, CCl_3F) δ -86.5 (s, unreacted C_5F_5N 2,6 position, 1F), -91.0 (m, 2,6 position C_5F_4N, 2F), -132.6 (m, unreacted C_5F_5N 4 position, 0.6F), -158.5 (m, 3,5 position C_5F_4N, 2F), -160.7 (m, unreacted C_5F_5N 3,5 position, 1F).

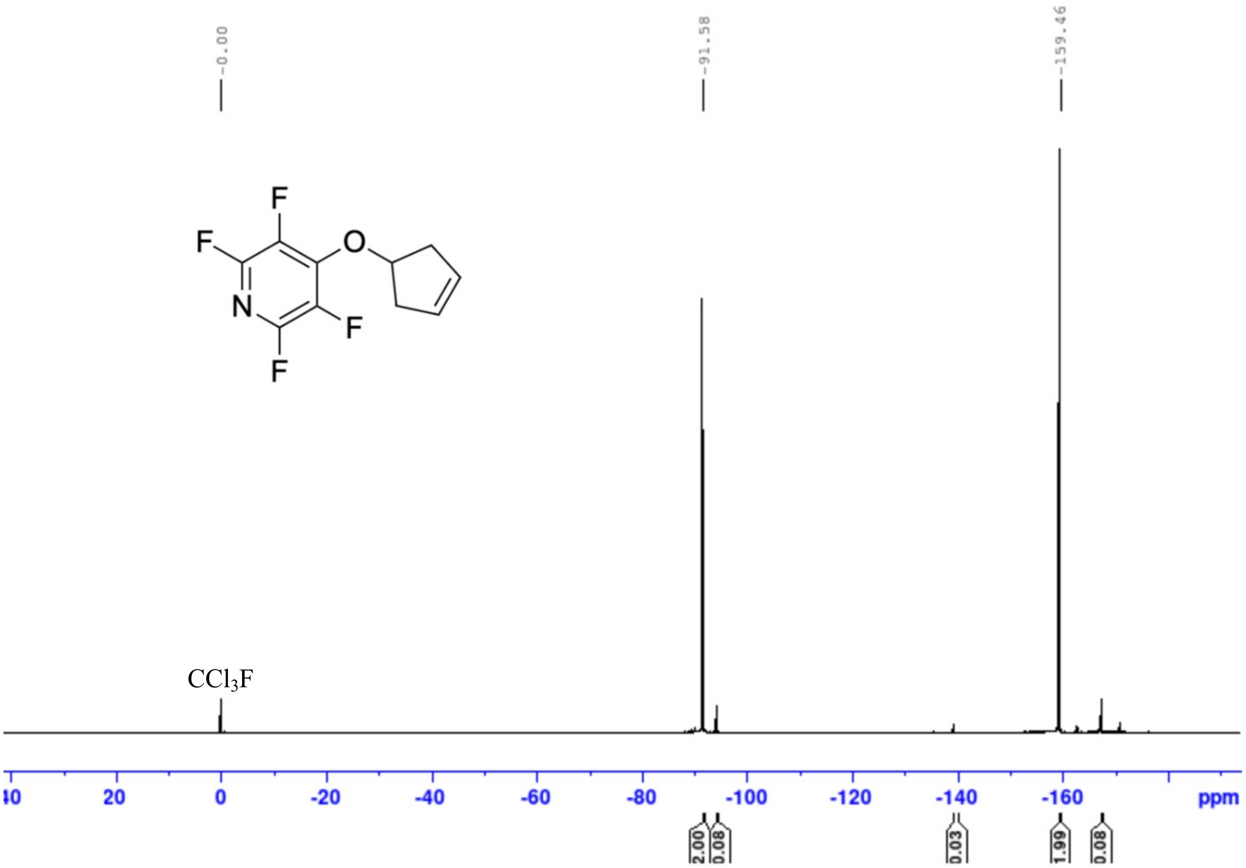

Figure S6. ^{19}F NMR of **6**.

^{19}F NMR ($CDCl_3$, 376 MHz, CCl_3F) δ -91.6 (m, 2,6 position C_5F_4N, 2F), -94.3 (t, unreacted C_5F_5N position 2 and 6, ^3J = 24 Hz, 0.06F), -139.4 (dt, unreacted C_5F_5N, ^3J = 18 Hz, ^4J= 12 Hz, 0.03F), -159.5 (m, 3,5 position C_5F_4N, 2F), -167.4 (d, unreacted C_5F_5N position 3 and 5, ^3J=23Hz, 0.08F).

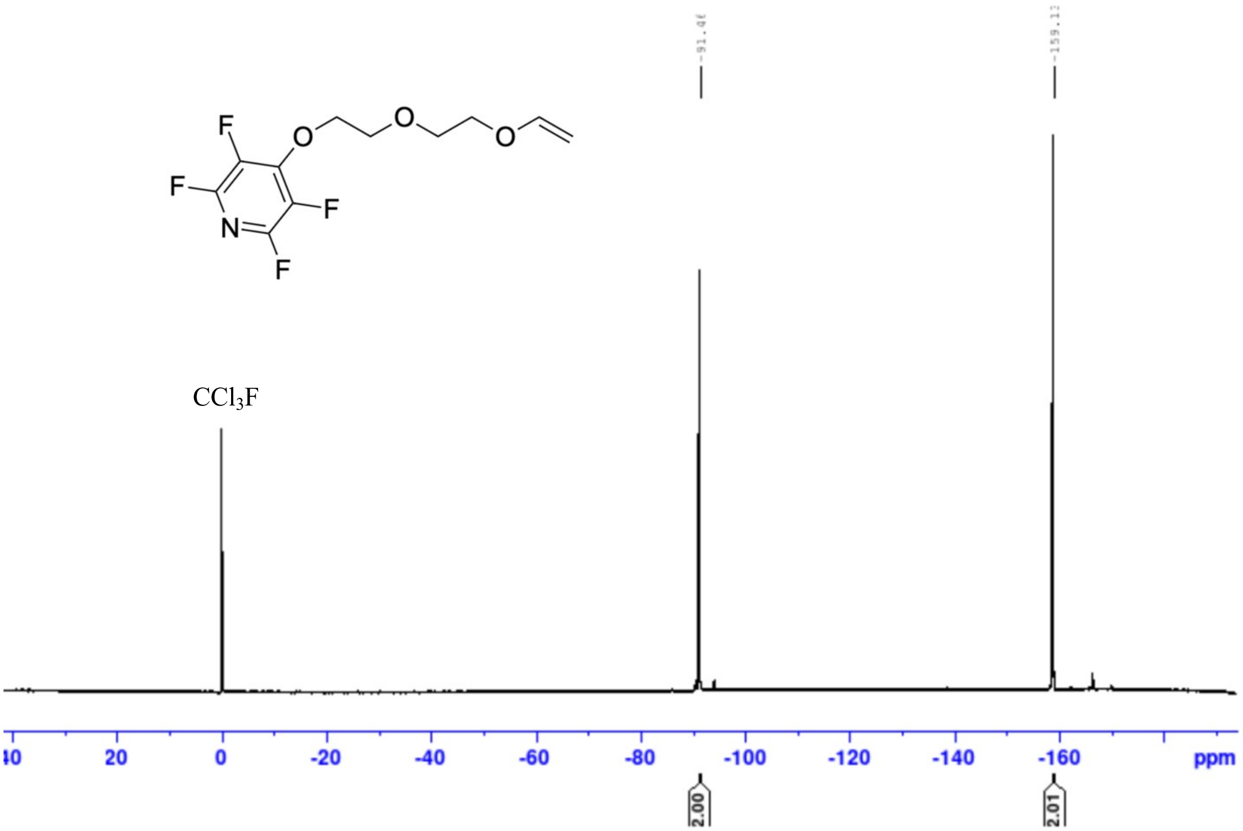

Figure S7. ^{19}F NMR of **7**.

^{19}F NMR ($CDCl_3$, 376 MHz, CCl_3F) δ -91.2 (m, 2,6 position C_5F_4N, 2F), -159.0 (m, 3,5 position C_5F_4N, 2F).

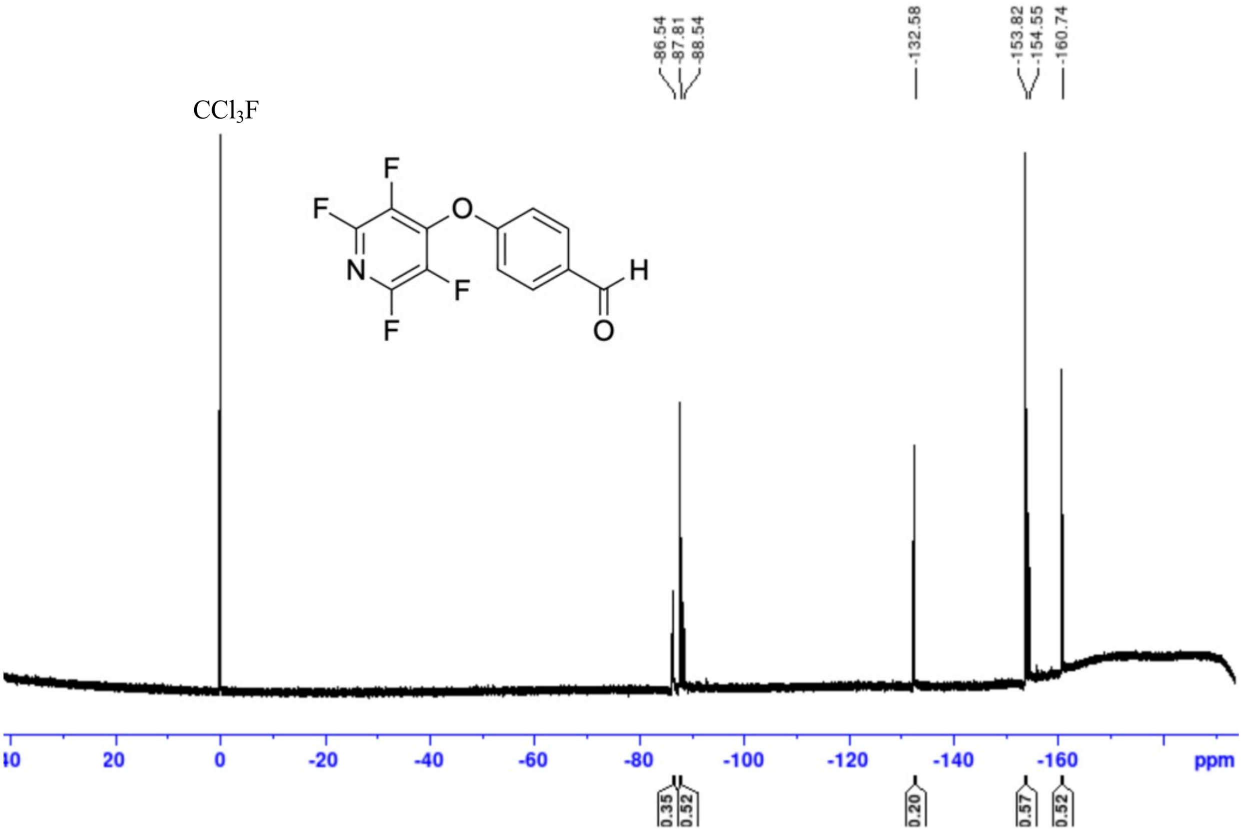

Figure S8. ^{19}F NMR of **8**.

^{19}F NMR ($CDCl_3$, 376 MHz, CCl_3F) δ -86.5 (s, unreacted C_5F_5N 2, 6 position, 1F) -87.8 (m, 2,6 position C_5F_4N, 2F), -132. 6 (m, unreacted C_5F_5N 4 position, 0.5F), -153.8 (m, 3,5 position C_5F_4N, 2F), -160.7 (m, unreacted C_5F_5N 2, 6 position, 1F).

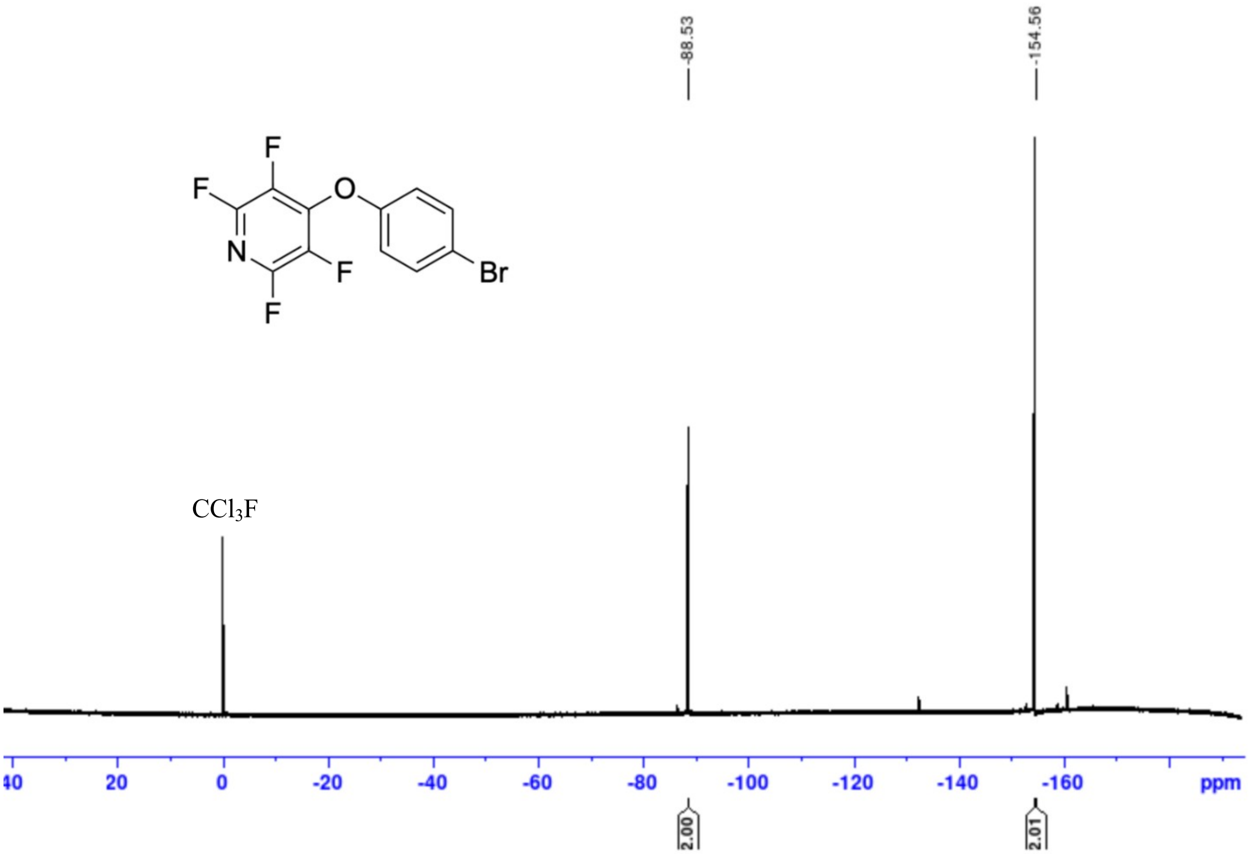

Figure S9. ^{19}F NMR of **9**.

^{19}F NMR ($CDCl_3$, 376 MHz, CCl_3F) δ -88.5 (m, 2,6 position C_5F_4N, 2F), -154.6 (m, 3,5 position C_5F_4N, 2F).

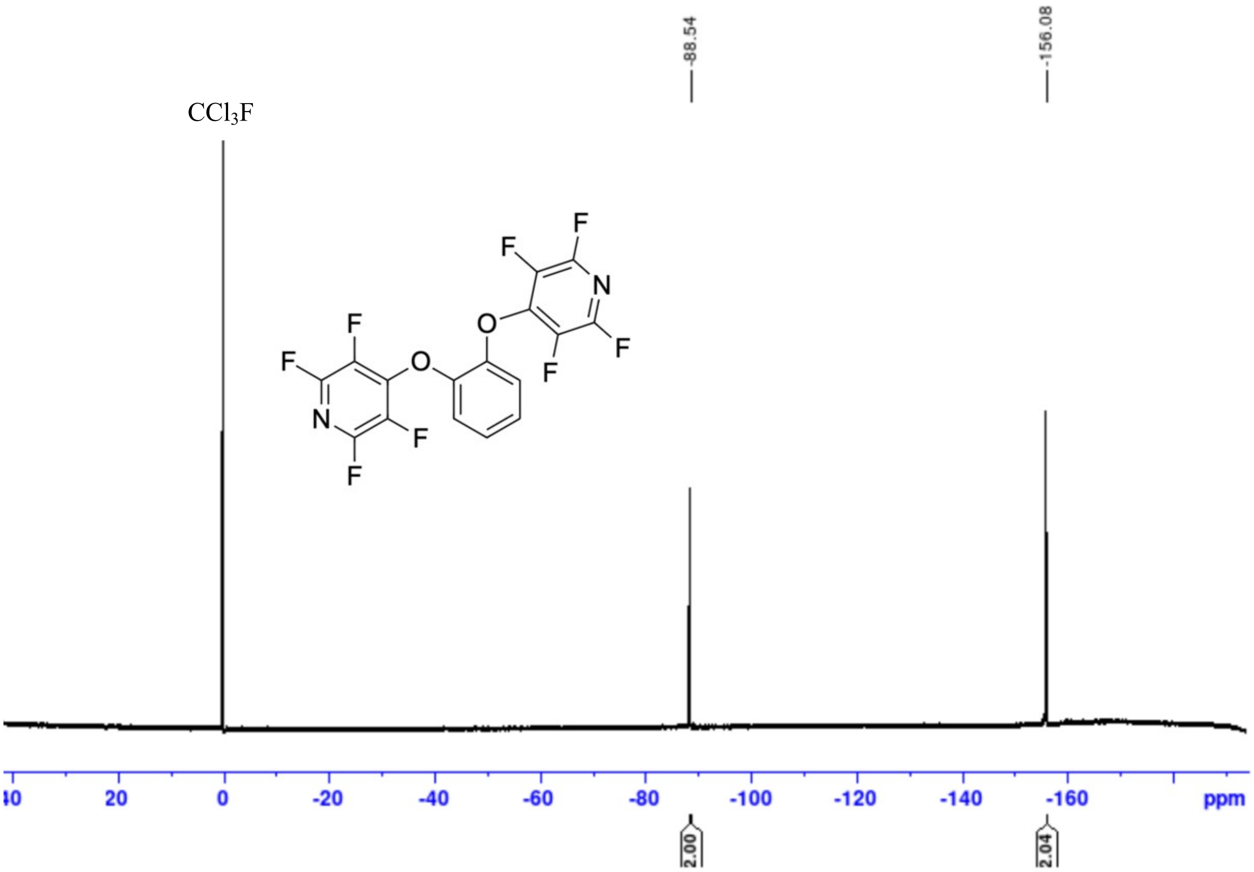

Figure S10. ^{19}F NMR of **10**.

^{19}F NMR ($CDCl_3$, 376 MHz, CCl_3F) δ -88.5 (m, 2,6 position C_5F_4N, 4F), -156.1 (m, 3,5 position C_5F_4N, 4F).

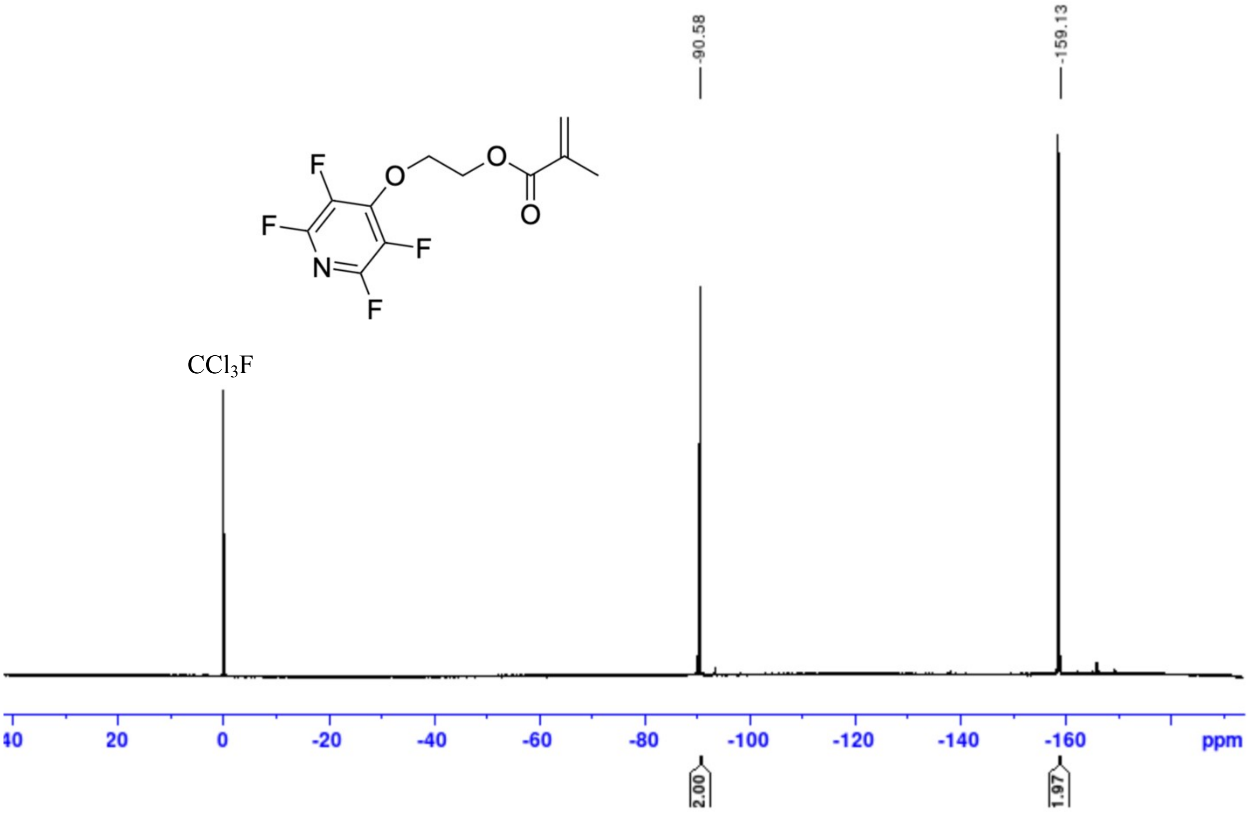

Figure S11. ^{19}F NMR of **11**.

^{19}F NMR ($CDCl_3$, 376 MHz, CCl_3F) δ -90.6 (m, 2,6 position C_5F_4N, 2F), -159.1 (m, 3,5 position C_5F_4N, 2F).

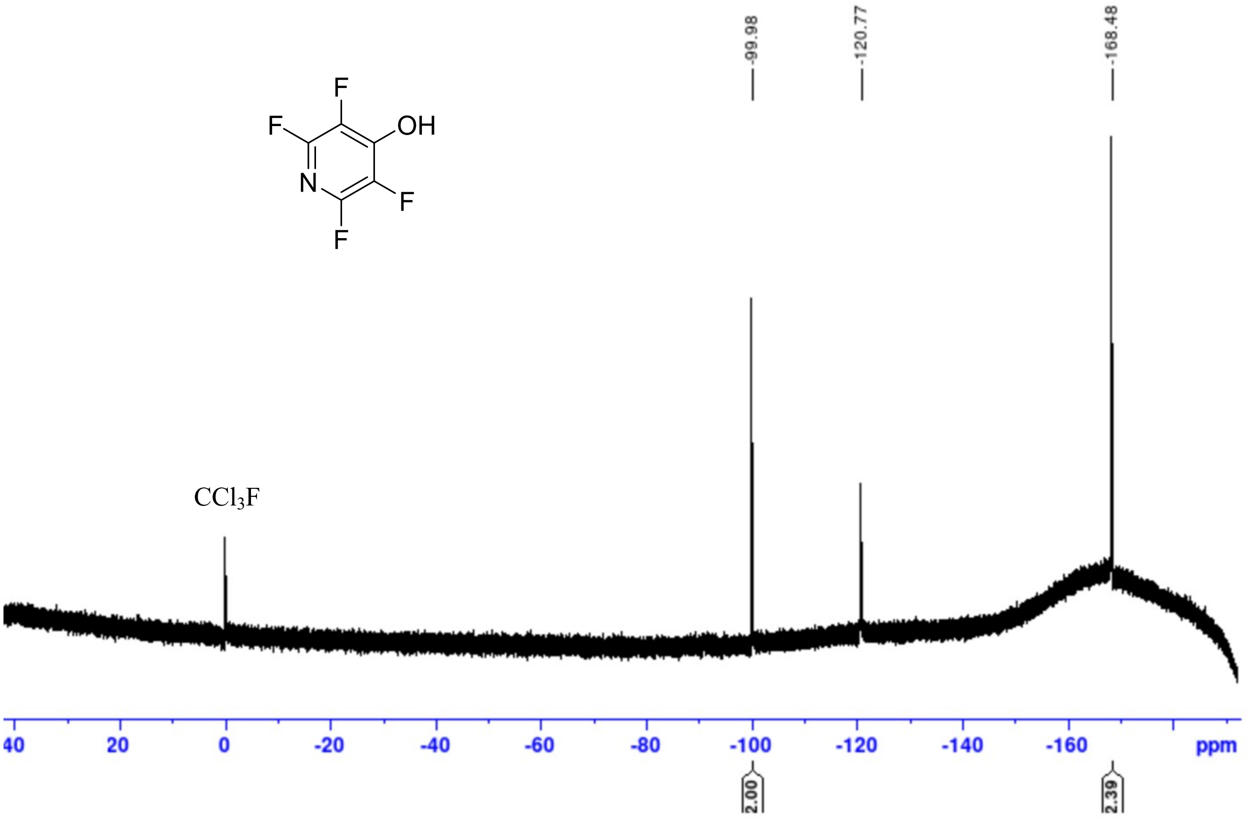

Figure S12. ^{19}F NMR of **12**.

^{19}F NMR (D_2O, 376 MHz, CCl_3F) δ -100.0 (m, 2,6 position C_5F_4N, 2F), -120.8 (s, F^-), -168.5 (m, 3,5 position C_5F_4N, 2F).

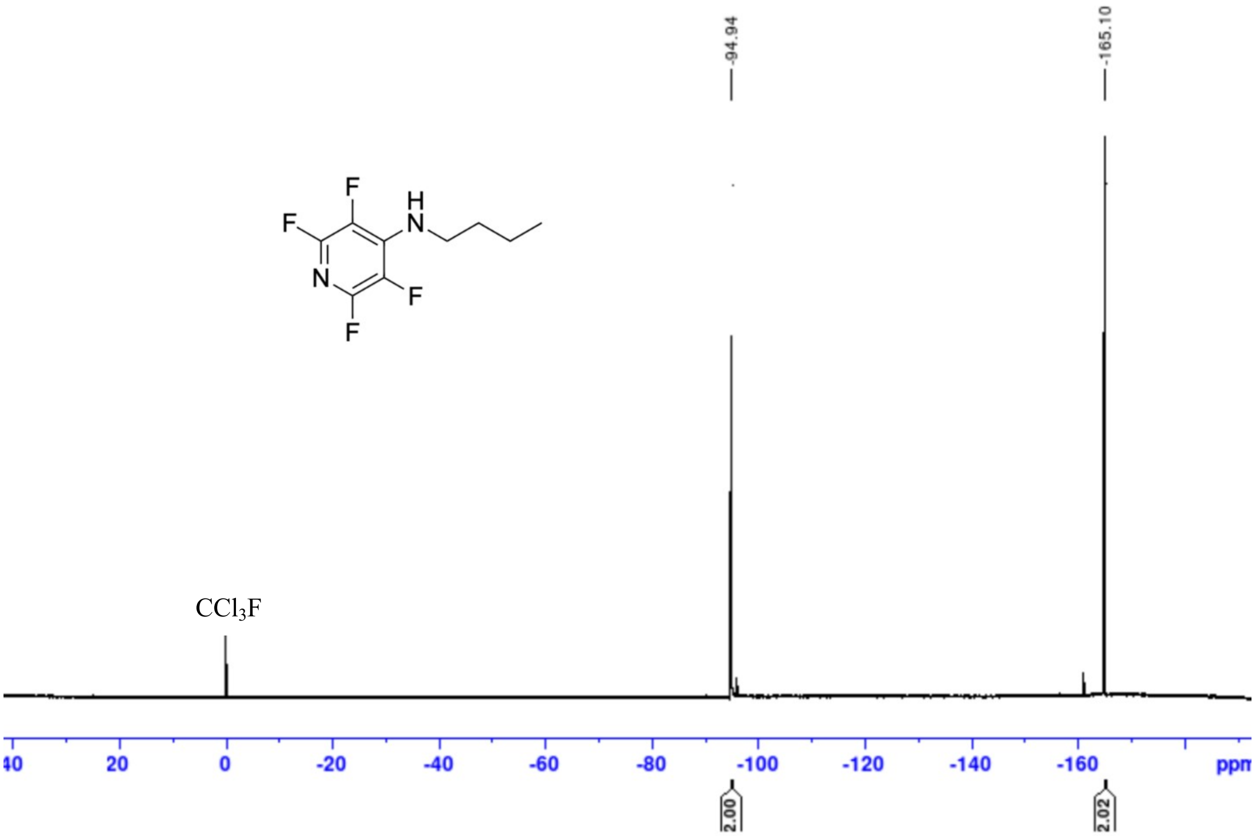

Figure S13. ^{19}F NMR of **13**.

^{19}F NMR ($CDCl_3$, 376 MHz, CCl_3F) δ -94.9 (m, 2,6 position C_5F_4N, 2F), -165.1 (m, 3,5 position C_5F_4N, 2F).

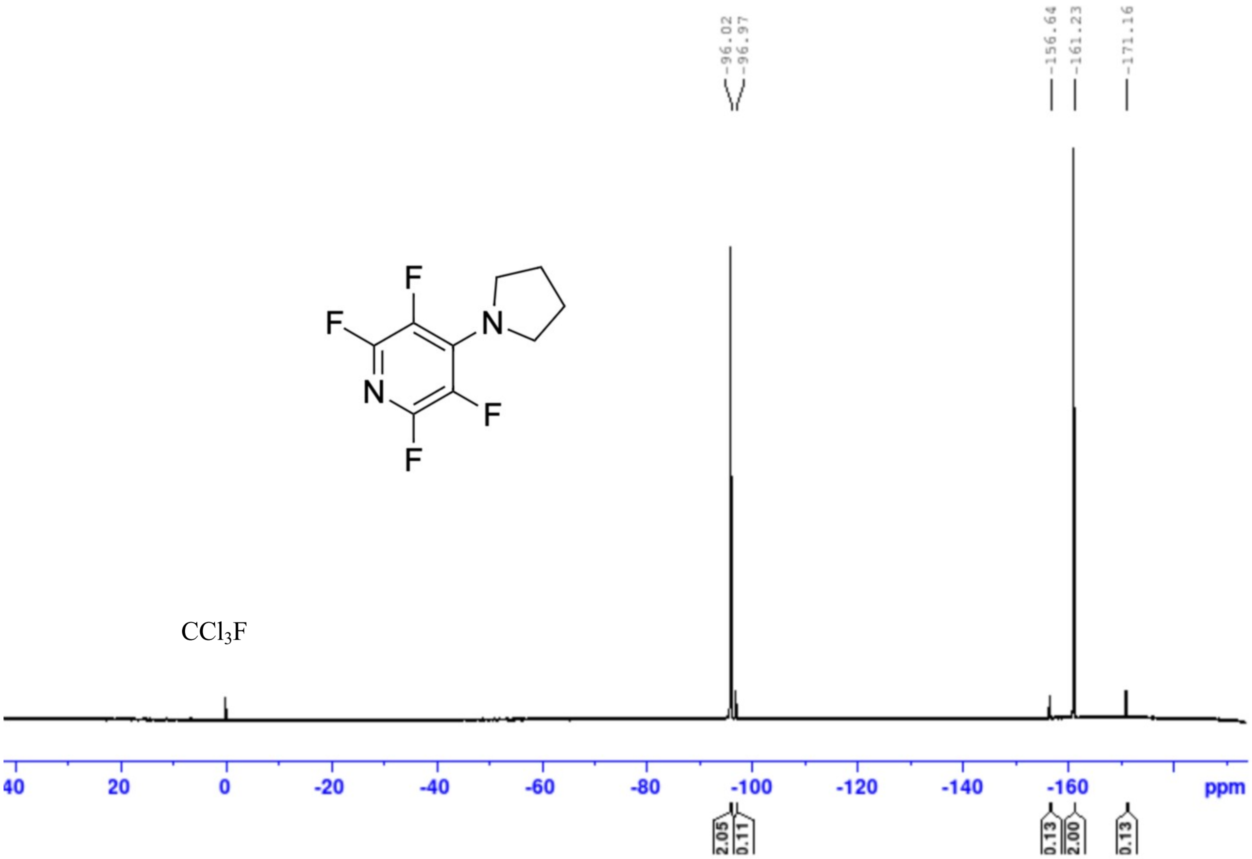

Figure S14. ^{19}F NMR of **14**.

^{19}F NMR ($CDCl_3$, 376 MHz, CCl_3F) δ -96.0 (m, 2,6 position C_5F_4N, 2F), -97.0 (dd, di 5 position C_5F_3N , ^3J = 24 Hz, ^4J = 25 Hz, 1F), -156.6 (dm, di 6 position C_5F_3N , ^3J = 23 Hz, 1F), -161.2 (m, 3,5 position C_5F_4N, 2F), - 171.2 (dm, di 3 position C_5F_3N, ^4J = 26 Hz, 1F).

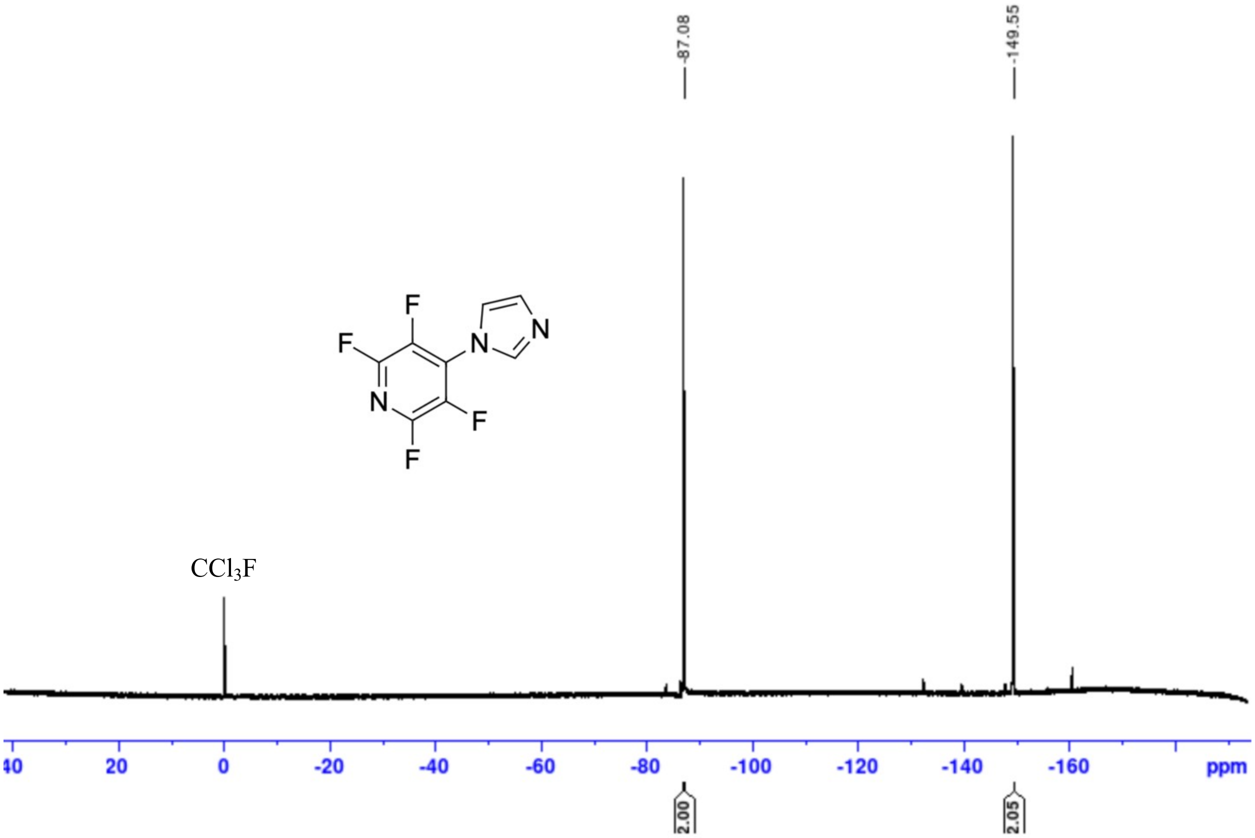

Figure S15. ^{19}F NMR of **15**.

^{19}F NMR ($CDCl_3$, 376 MHz, CCl_3F) δ -87.1 (m, 2,6 position C_5F_4N, 2F), -150.0 (m, 3,5 position C_5F_4N, 2F).

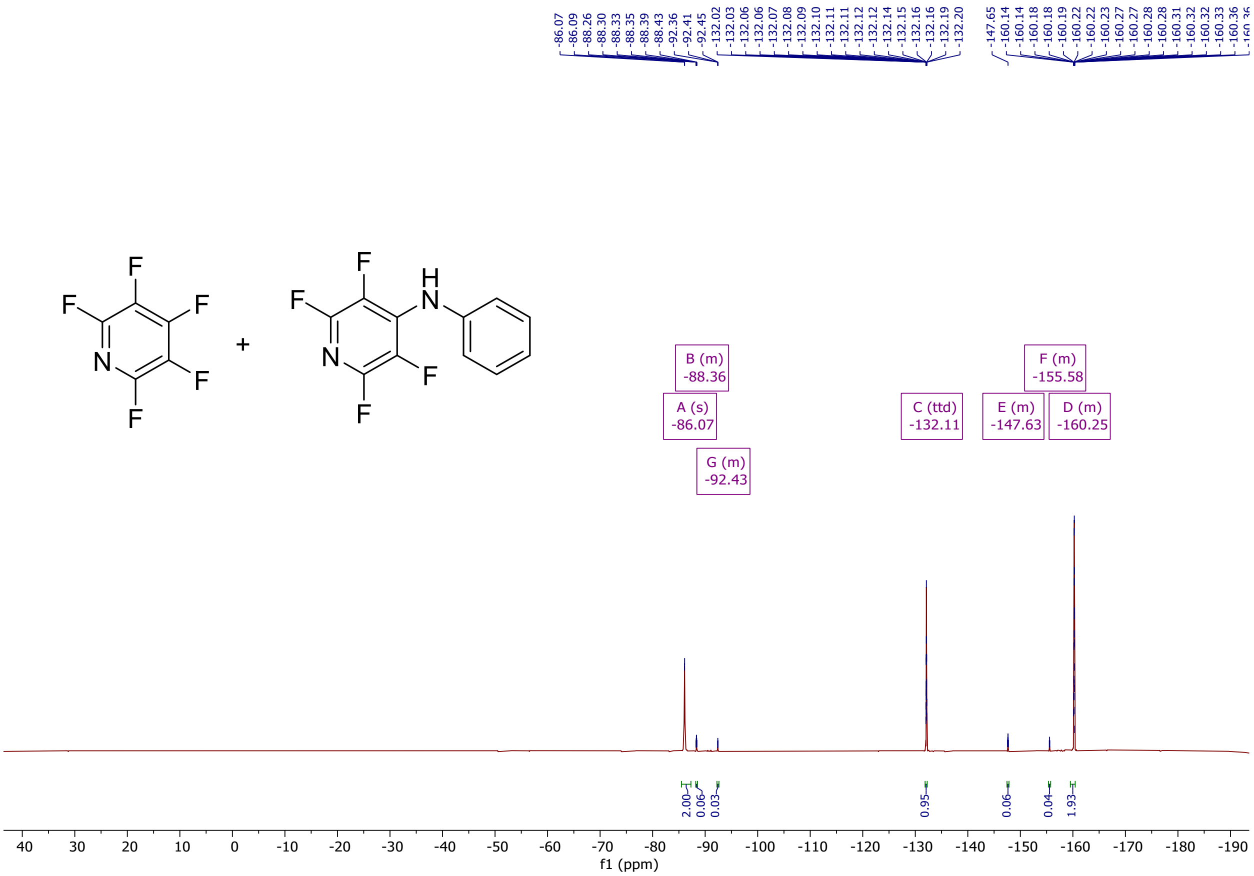

Figure S16. ^{19}F NMR of **16**.

^{19}F NMR (376 MHz, $CDCl_3$) δ -86.07 (m, 2F), -88.36 (m, 0.06F), -92.43 (m, 0.03F), -132.11 (m, 1F), -147.63 (m, 0.06F), -155.58 (m, 0.04F), -160.25 (m, 2F).

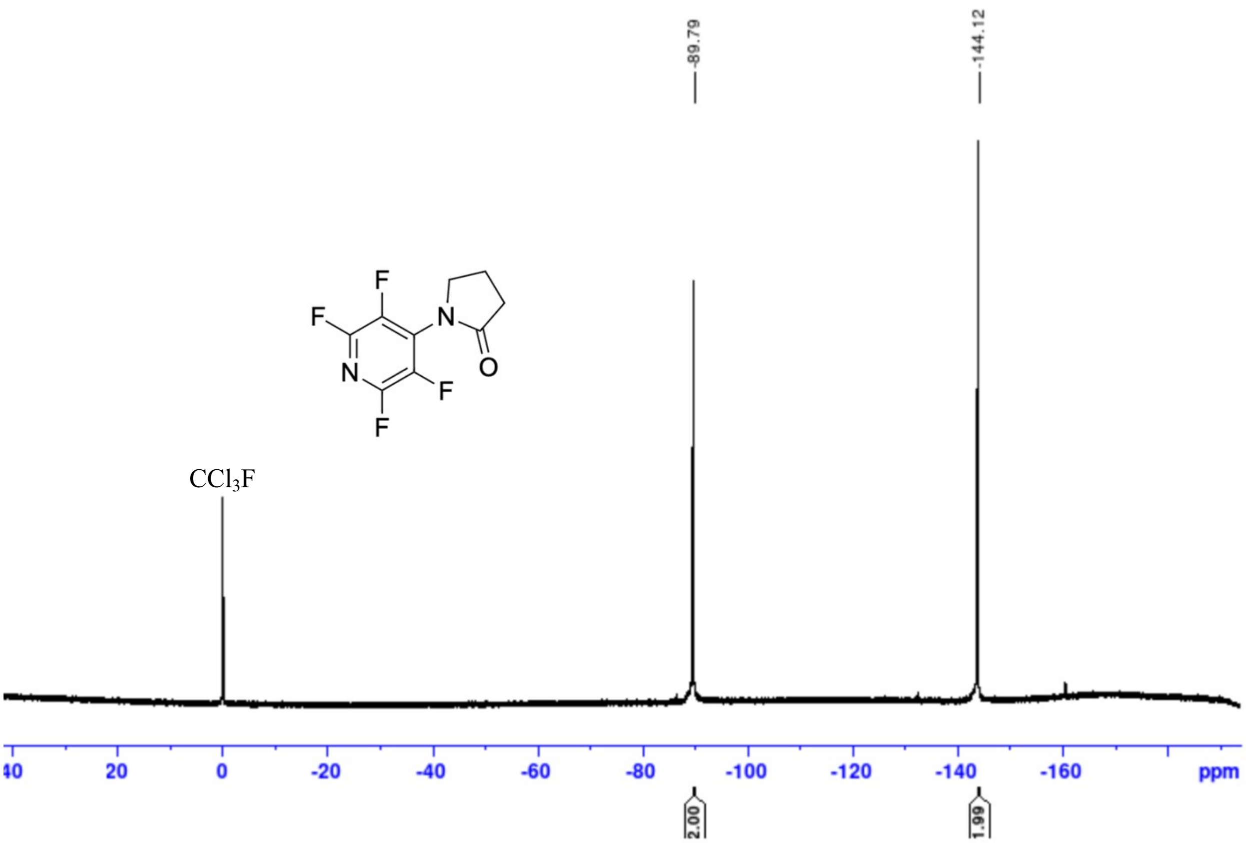

Figure S17. ^{19}F NMR of **17**.

^{19}F NMR ($CDCl_3$, 376 MHz, CCl_3F) δ -90.0 (m, 2,6 position C_5F_4N, 2F), -144.1 (m, 3,5 position C_5F_4N, 2F).

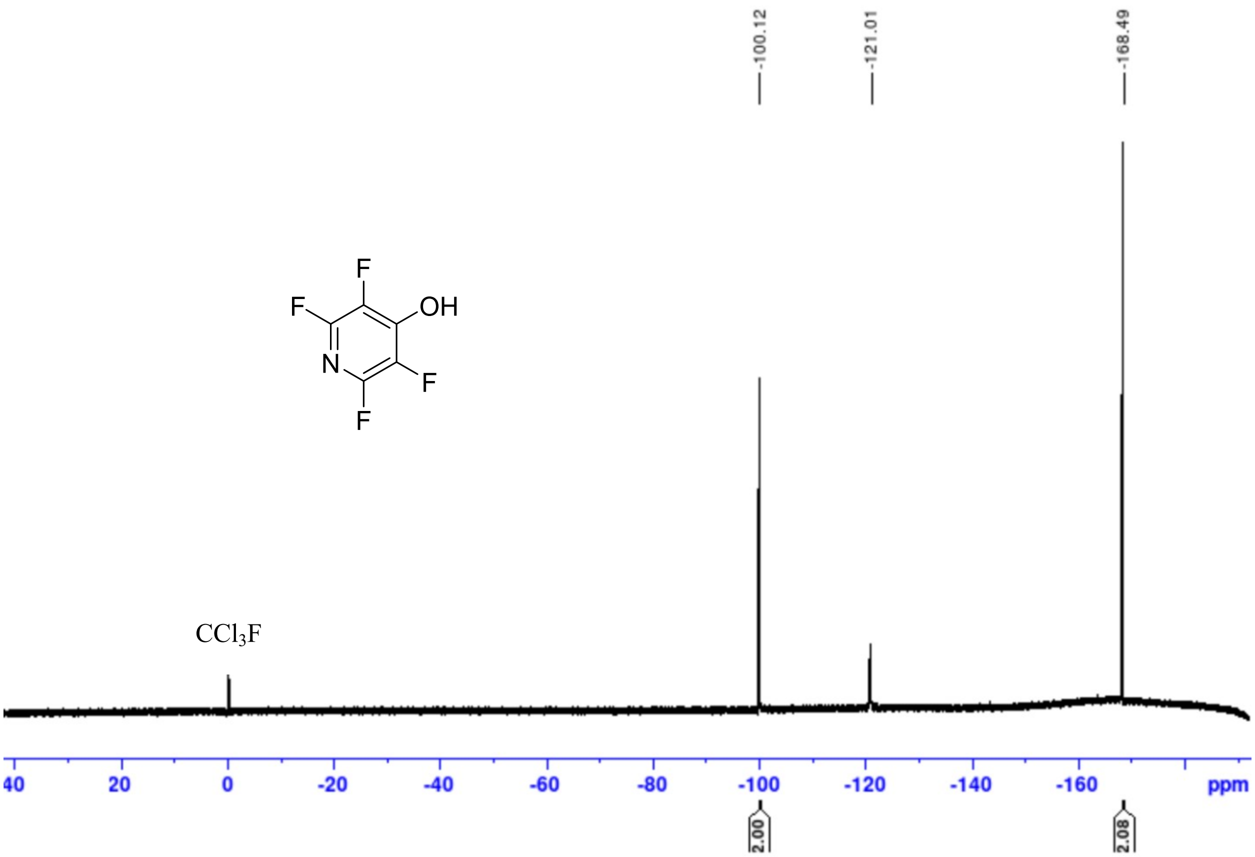

Figure S18. ^{19}F NMR of **18**.

^{19}F NMR (D_2O, 376 MHz, CCl_3F) δ -100.1 (m, 2,6 position C_5F_4N, 2F), -121.0 (s, F^-), -168.5 (m, 3,5 position C_5F_4N, 2F).

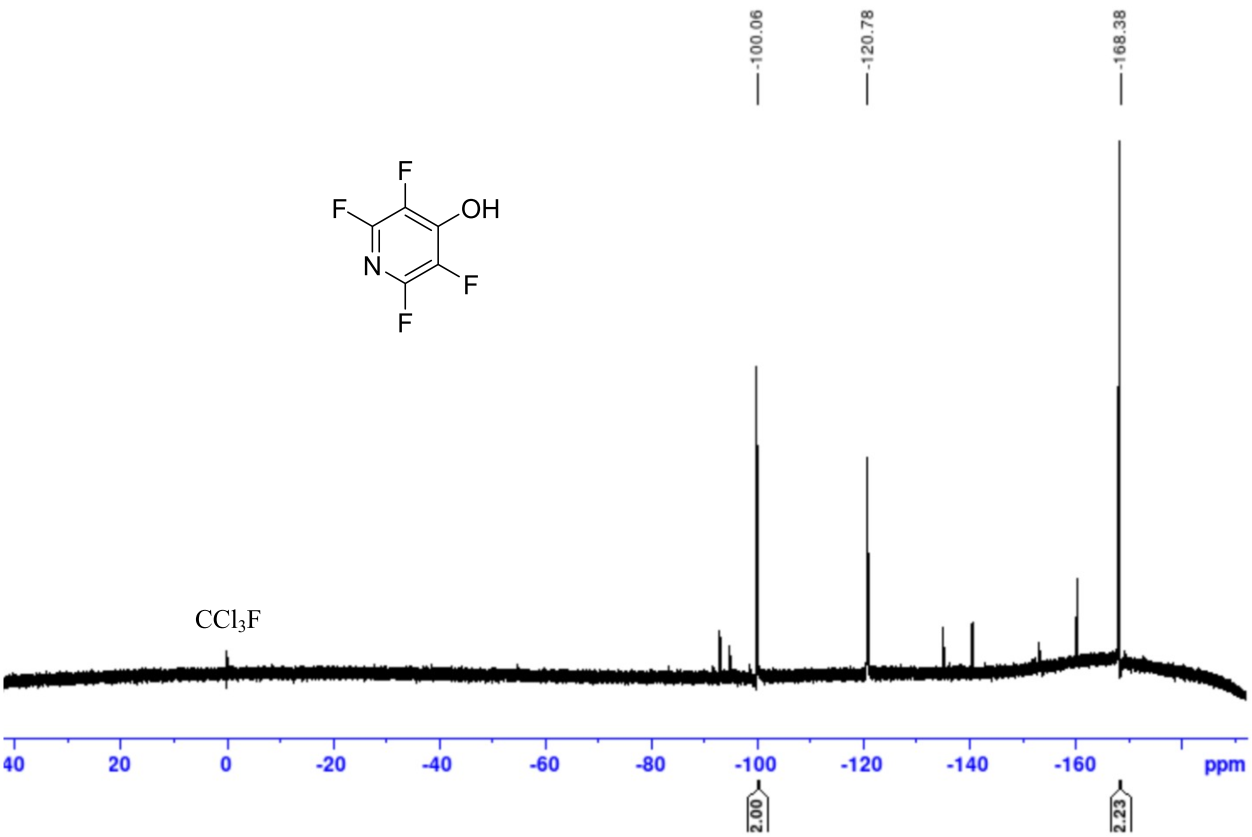

Figure S19. ^{19}F NMR of **19**.

^{19}F NMR (D_2O, 376 MHz, CCl_3F) δ -100.1 (m, 2,6 position C_5F_4N, 2F), -120.8 (s, F^-), -168.4 (m, 3,5 position C_5F_4N, 2F).

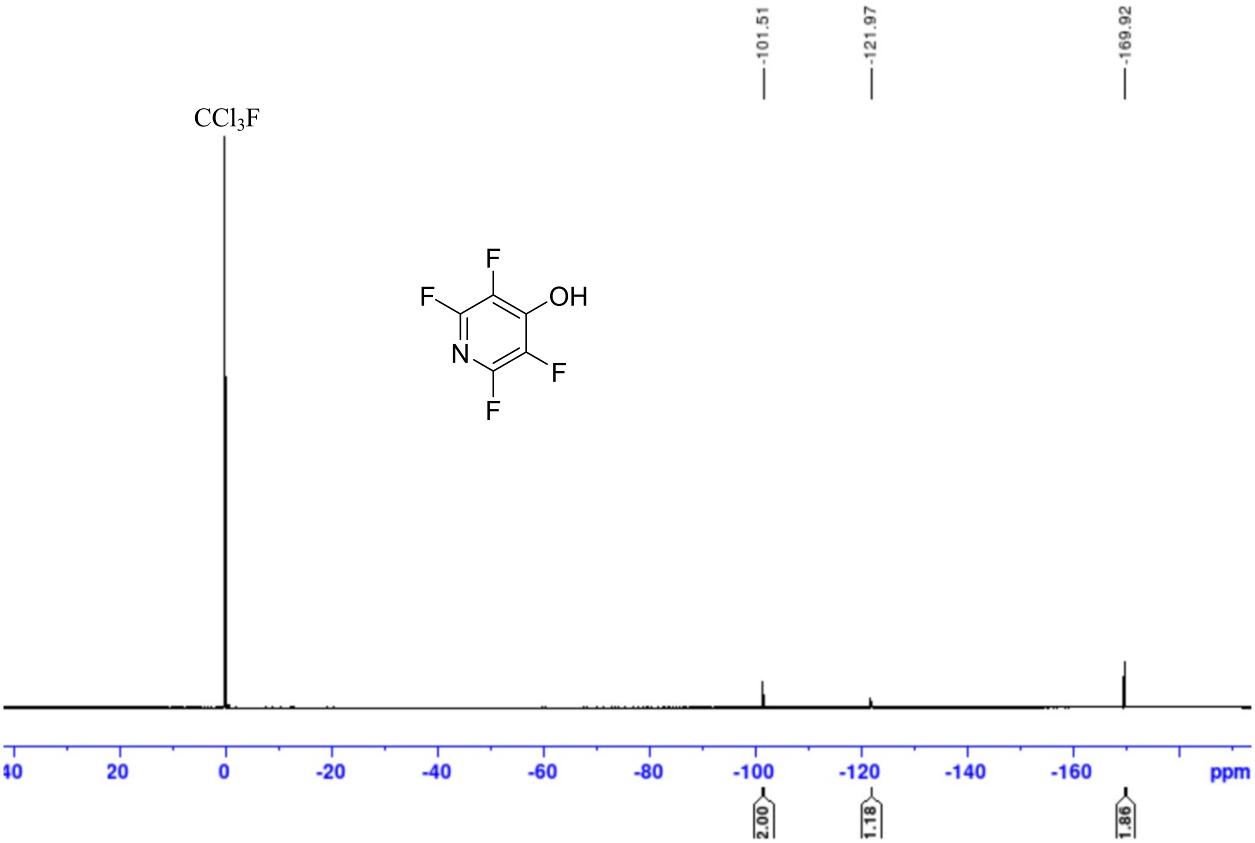

Figure S20. ^{19}F NMR of **20**.

^{19}F NMR (D_2O, 376 MHz, CCl_3F) δ -101.5 (m, 2,6 position C_5F_4N, 2F), -122.0 (s, F^-), -169.9 (m, 3,5 position C_5F_4N, 2F).

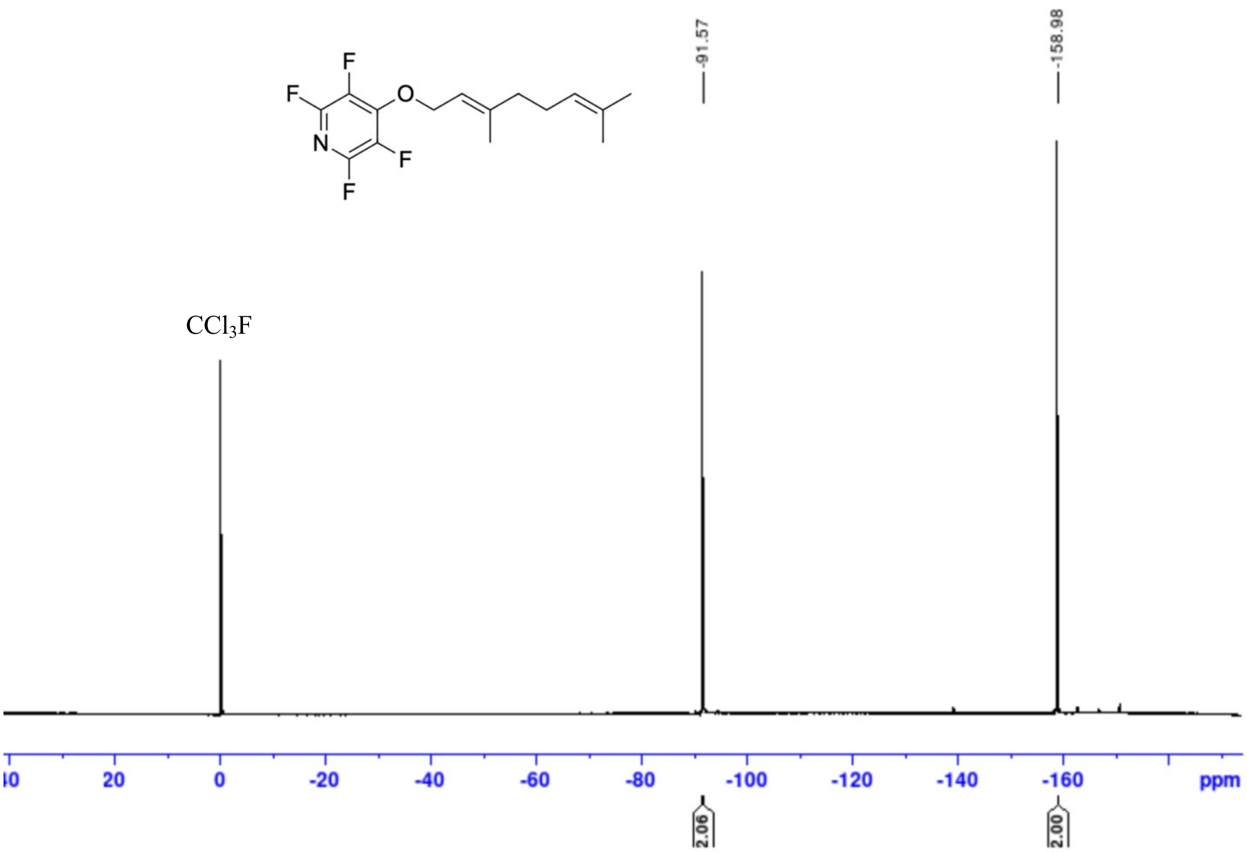

Figure S21. ^{19}F NMR of **25**.

^{19}F NMR ($CDCl_3$, 376 MHz, CCl_3F) δ -91.6 (m, 2,6 position C_5F_4N, 2F), -159.0 (m, 3,5 position C_5F_4N, 2F).

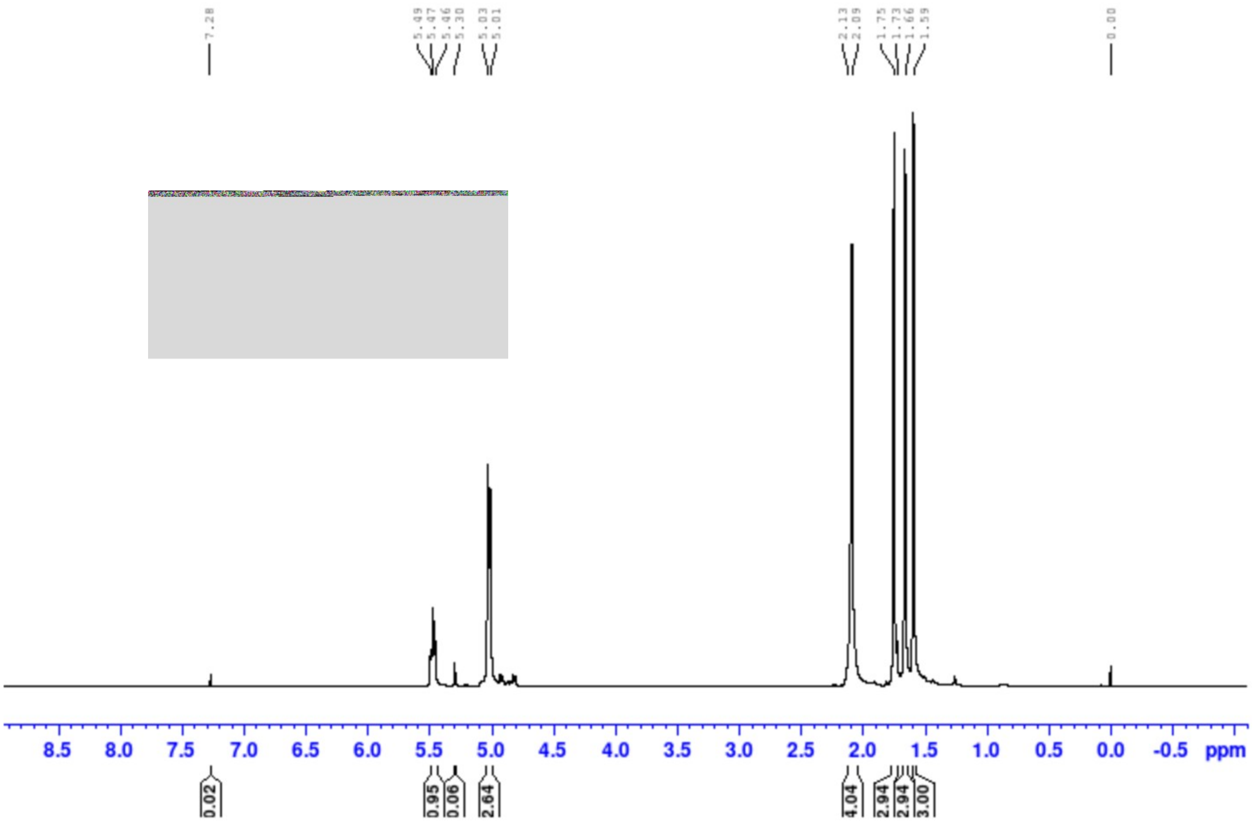

Figure S22. ^{1}H NMR of **25**.

^{1}H NMR ($CDCl_3$, 400 MHz) d 5.47 (t, -O-CH_2-**CH**=C-, ^3J = 6.94Hz ,1H), 5.02 (s, -**CH**=C(CH_3)$_2$, 1H), 5.00 (s, -O-**CH_2**-CH=, 2H), 2.08 (s, -**CH_2CH_2**-CH=C-, 4H), 1.74 (s, -O-CH_2-CH=C-**CH_3**, 3H), 1.66 (s, -C-(**CH_3**)$_2$, 3H), 1.59 (s, -C-(**CH_3**)$_2$, 3H).

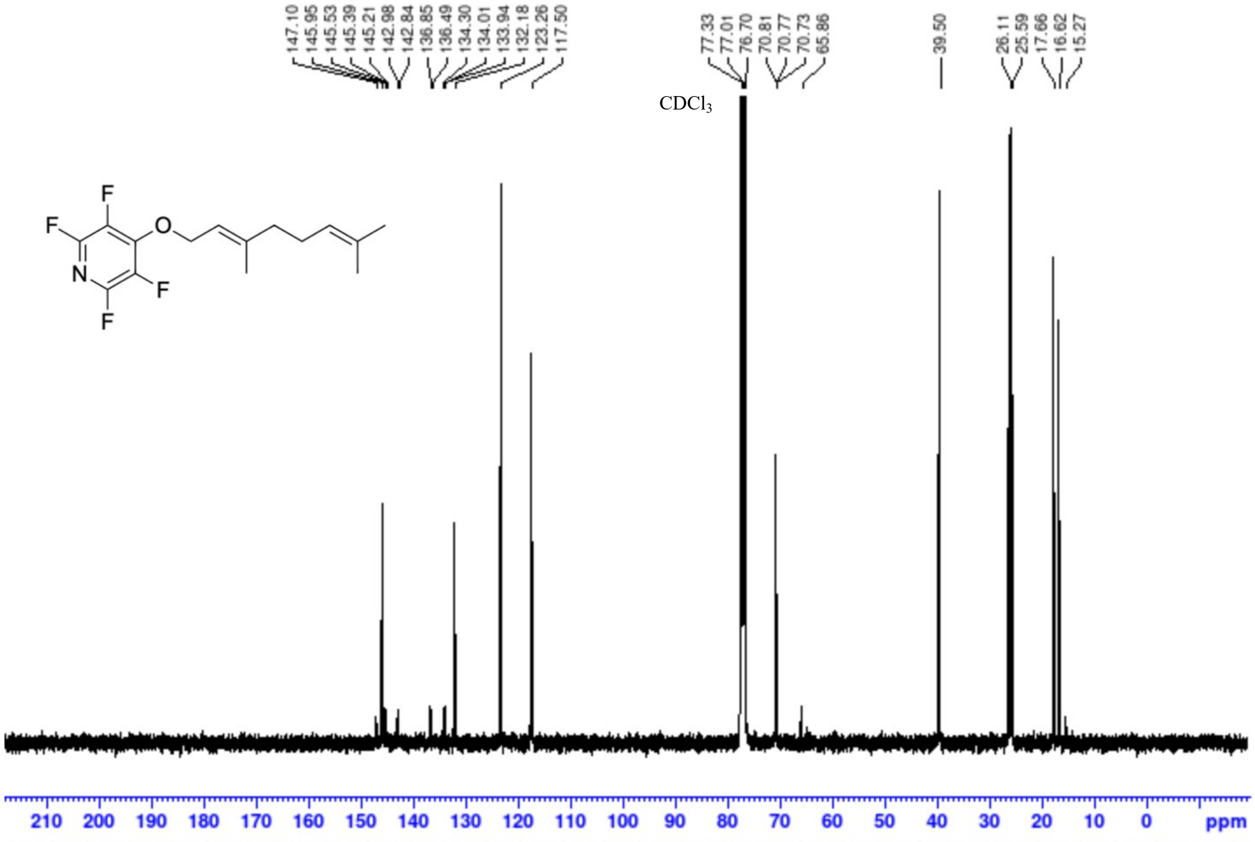

Figure S23. ^{13}C NMR of **25**.

^{13}C NMR ($CDCl_3$, 100MHz) d 147.2 (m, 4 position C_5F_4N), 146.1 (s, -CH=C(CH_3)-CH_2-), 144.3 (dm, 3,5 position C_5F_4N, ^1J = 242 Hz), 135.5 (dm, 2,6 position C_5F_4N, ^1J = 256 Hz), 132.3 (s, -CH=**C**(CH_3)$_2$), 123.4 (s, -**CH**=C(CH_3)$_2$), 117.6 (s, -CH_2-**CH**=(CH_3)-CH_2-), 70.9 (t, -O-**CH_2**-CH=, ^4J = 4.3 Hz), 39.6 (s, =C(CH_3)-**CH_2**-), 26.3 (s, =C(CH_3)-CH_2-**CH_2**-), 25.7 (s, =C(**CH_3**)-CH_3), 17.8 (s, =C(CH_3)-**CH_3**), 16.8 (s, =C(**CH_3**)-CH_2-).

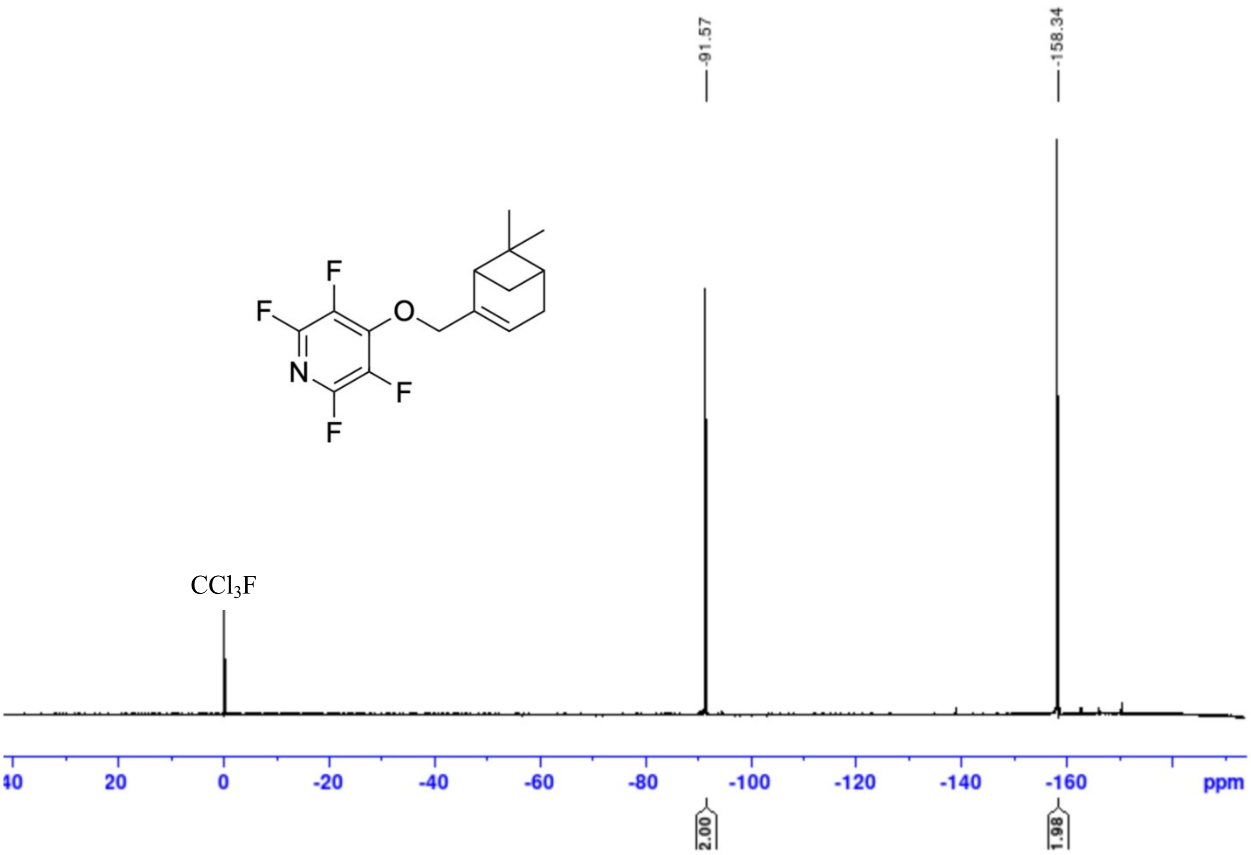

Figure S24. ^{19}F NMR of **26**.

^{19}F NMR ($CDCl_3$, 376 MHz, CCl_3F) δ -91.6 (m, 2,6 position C_5F_4N, 2F), -158.3 (m, 3,5 position C_5F_4N, 2F).

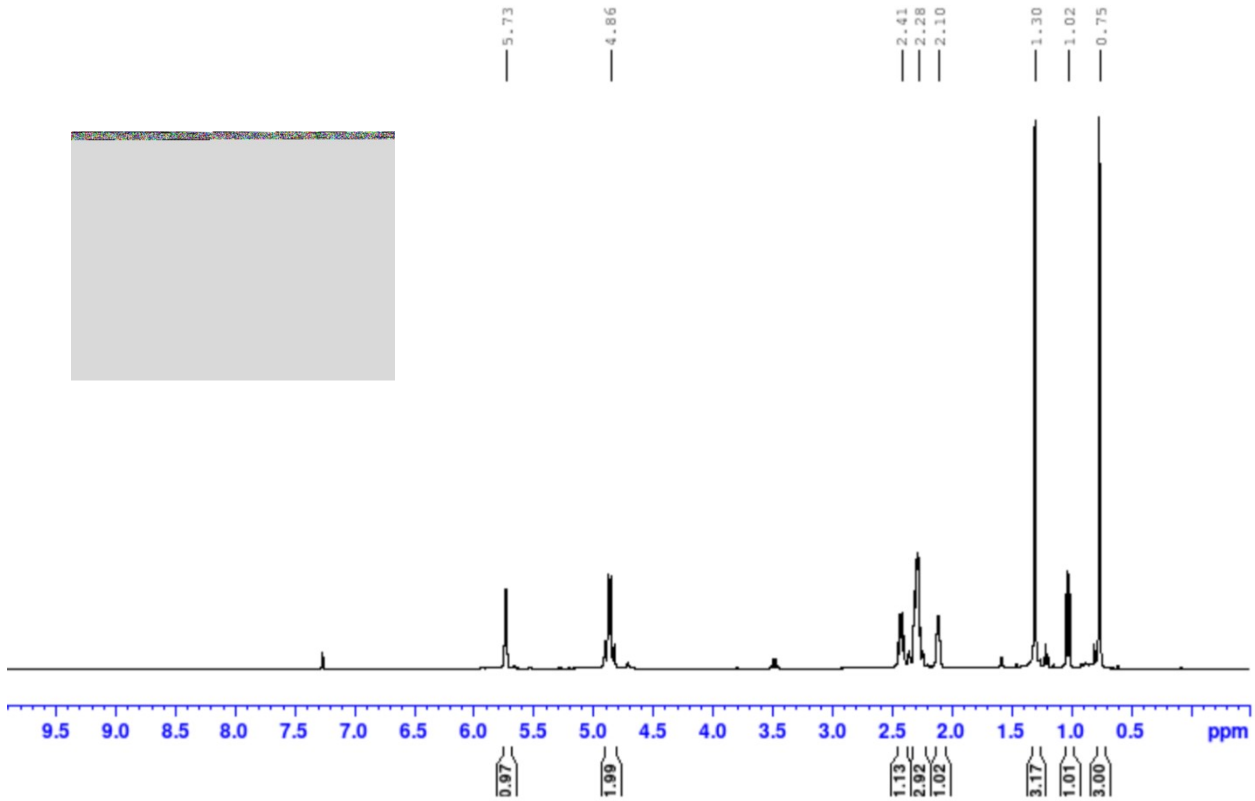

Figure S25. ^{1}H NMR of **26**.

^{1}H NMR ($CDCl_3$, 400 MHz) d 5.72 (s, -C=**CH**-,1H), 4.86 (dd, -O-**CH$_2$-**, ^4J = 11.90 Hz, ^5J = 19.99 Hz, 2H), 2.41 (dt, -O-C-**CH**-C(CH_3)$_2$-, ^3J = 5.59 Hz, ^4J = 8.80 Hz, 1H), 2.28 (m, =CH-**CH$_2$**-CH, 2H), 2.10 (s, **-CH**-CH_2-CH= and -CH-**CH**$_2$-CH-, 3H), 1.30 (s, -C(**CH$_3$**)-CH_3), 1.02 (d, -CH-**CH**$_2$-CH-, ^3J = 6.56 Hz, 1H), 0.75 (s, -C(CH_3)-**CH$_3$,** 3H).

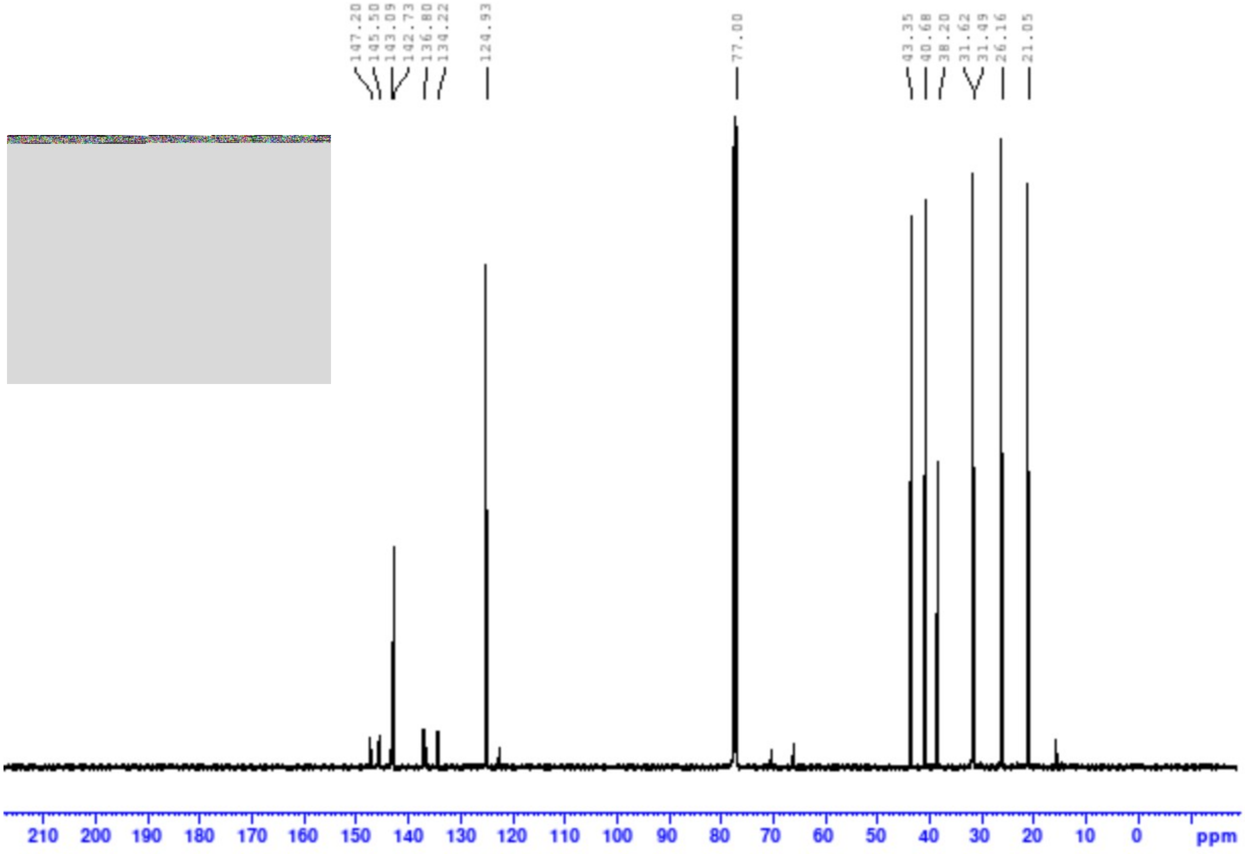

Figure S26. ^{13}C NMR of **26**.

^{13}C NMR ($CDCl_3$, 100MHz) d 147.2 (m, 4 position C_5F_4N), 144.3 (dm, 3,5 position C_5F_4N, ^1J = 242 Hz), 142.7 (s, -O-CH_2-**C**=), 135.5 (dm, 2,6 position C_5F_4N, ^1J = 257 Hz), 124.9 (s, -C=**CH**-CH_2), 77.0 (t, -O-**CH_2**-, ^4J = 4.40), 43.3 (s, -CH=C-**CH**-), 40.7 (s, -C(CH_3)$_2$-**CH**-CH_2-CH=), 38.2 (s, -CH-**C**(CH_3)$_2$-CH-), 31.6 (s, =CH-**CH_2**-CH-), 31.5 (s, =C-CH-**CH_2**-CH-), 26.1 (s, -C(**CH_3**)$_2$-), 21.0 (s, -C(**CH_3**)$_2$-).

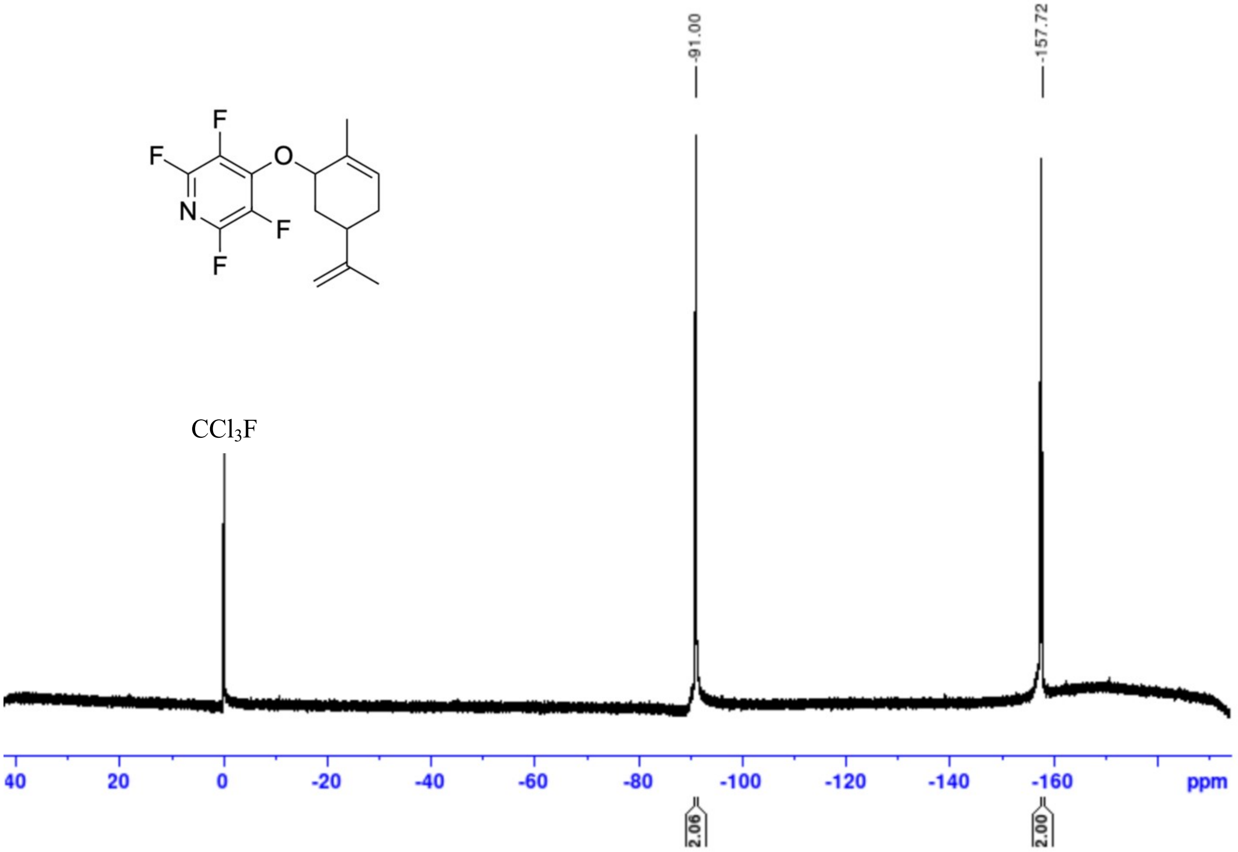

Figure S27. ^{19}F NMR of **27**.

^{19}F NMR ($CDCl_3$, 376 MHz, CCl_3F) δ -91.0 (m, 2,6 position C_5F_4N, 2F), -157.7 (m, 3,5 position C_5F_4N, 2F).

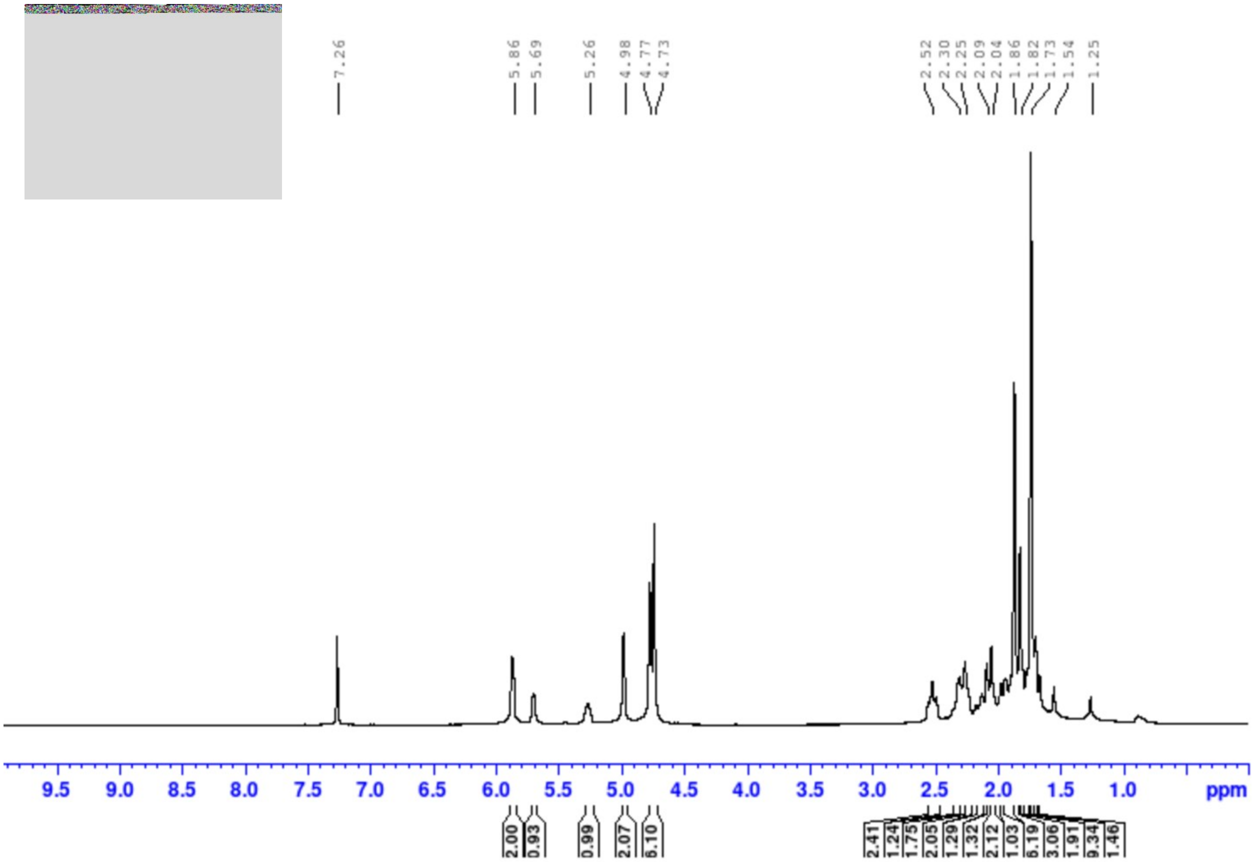

Figure S28. ^{1}H NMR of **27**.

^{1}H NMR ($CDCl_3$, 400 MHz) d 5.86 (m, -C(CH3)=**CH**-,2H), 5.69 (m, -C(CH3)=**CH**-,1H), 5.26 (s, -O-**CH**-, 1H), 4.97 (s, -O-**CH** -, 2H), 4.76 (s, -C=**CH$_2$**, 4H), 4.73 (s, -C=**CH$_2$**, 2H), 2.51 (t, -CH-C(**CH$_3$**)=CH_2, ^3J = 5.52 Hz, 2H), 2.36-1.90 (m, **-CH$_2$-C**(C(CH_3)=CH_2)**-CH$_2$-**, 13H), 1.86 (s, -C(**CH$_3$**)=CH_2, 6H), 1.82 (s, -C(**CH$_3$**)=CH_2, 3H), 1.73 (s,-O-CH-C(**CH$_3$**)=, 9H).

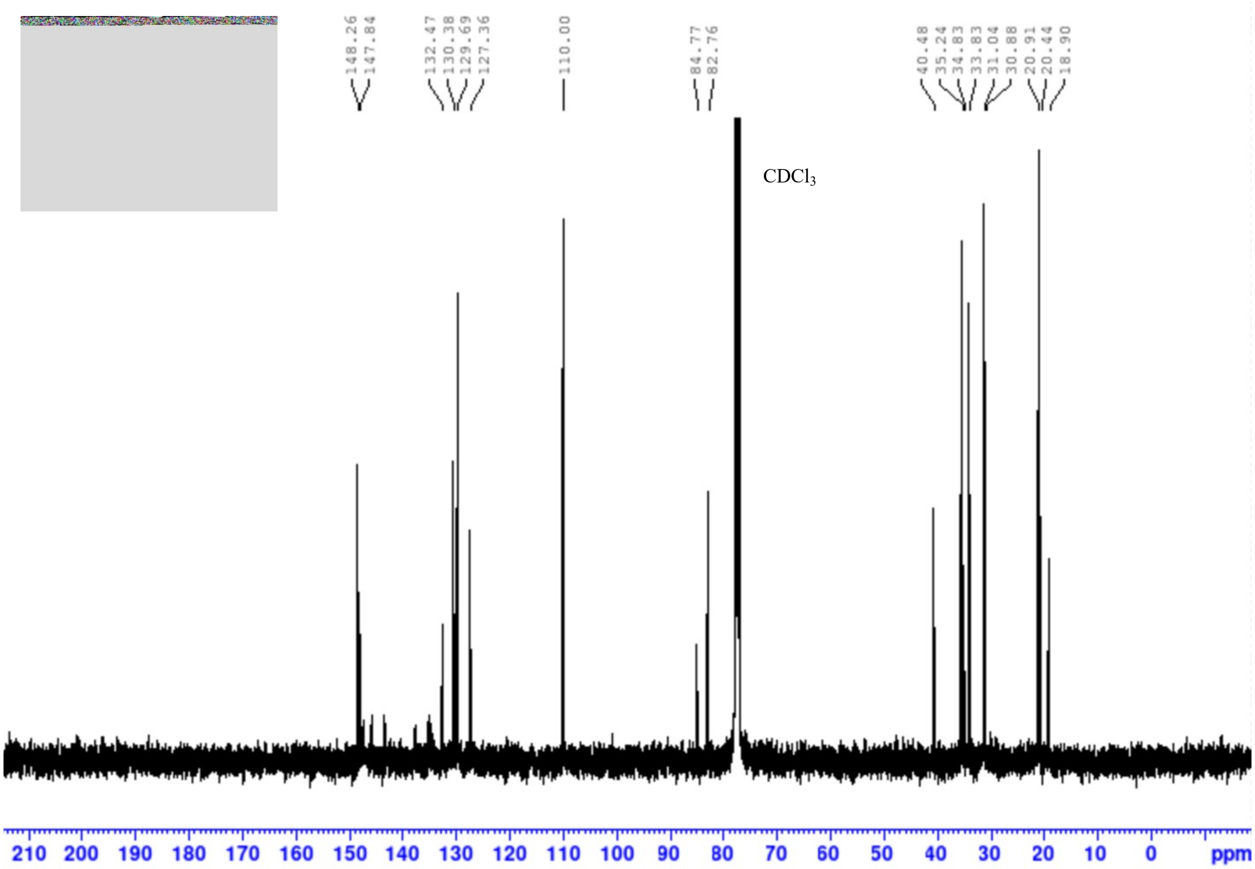

Figure S29. ^{13}C NMR of **27**.

^{13}C NMR ($CDCl_3$, 100MHz) d 148.2 and 147.8 (s, -**C**(CH_3)=CH_2), 147.4 (m, 4 position C_5F_4N), 144.4 (dm, 3,5 position C_5F_4N, ^1J = 242 Hz), 135.5 (dm, 2,6 position C_5F_4N, ^1J = 257 Hz), 132.5 and 130.4 (s, -O-CH-**C**(CH_3)=CH-), 129.6 and 127.3 (s, -CH-C(CH_3)=**CH**-), 110.0 and 109.8 (s, -C(CH_3)=**CH_2**), 84.8 (t, -O-**CH**-, ^4J = 3.43 Hz), 82.8 (t, -O-**CH**-, ^4J = 4.10 Hz), 40.5 and 35.2 (s, -CH_2-**CH**(C(CH_3)=CH_2)-CH_2-), 34.8 and 33.8 (s, -**CH_2**-CH(C(CH_3)=CH_2)-CH_2-), 31.0 and 30.9 (s, -CH_2-CH(C(CH_3)=CH_2)-**CH_2**-), 20.8 (s, -O-CH-C(**CH_3**)=CH-), 20.4 and 18.3 (s, -C(**CH_3**)=CH_2).

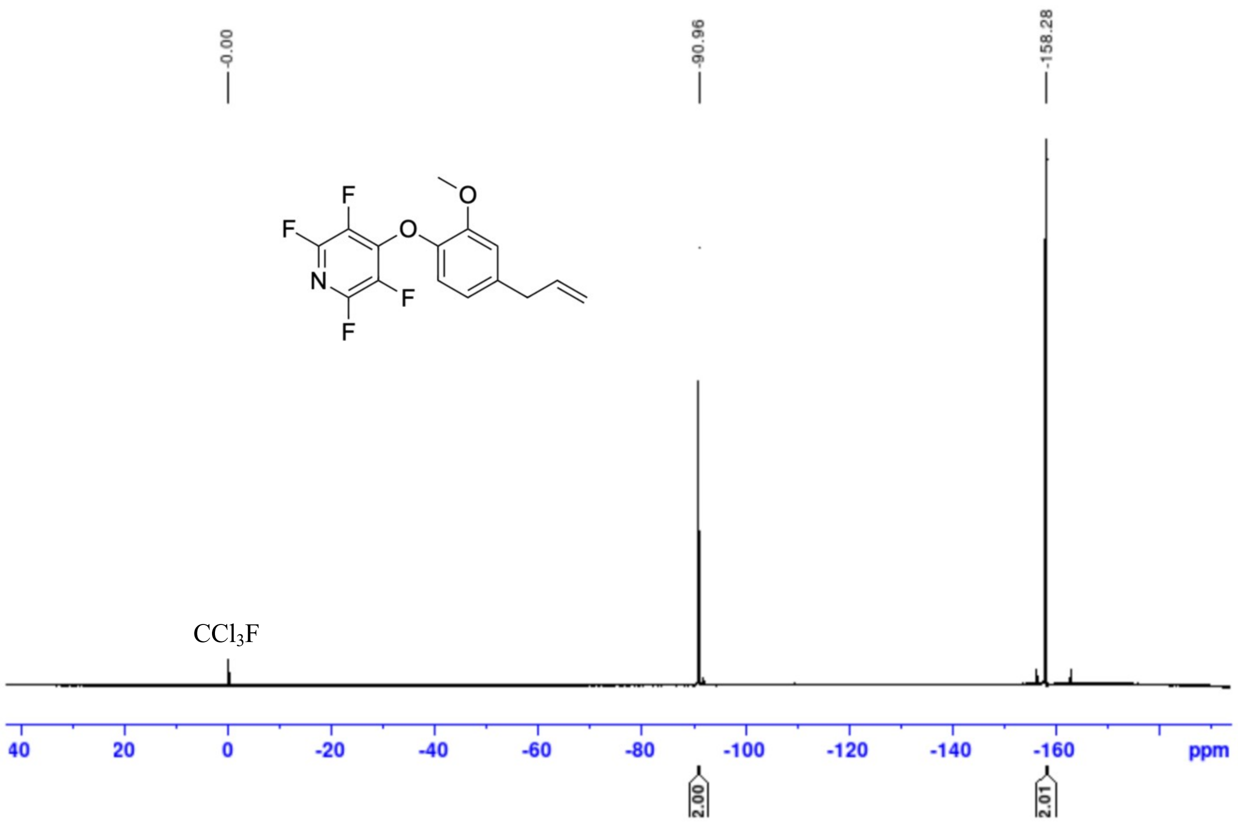

Figure S30. ^{19}F NMR of **28**.

^{19}F NMR ($CDCl_3$, 376 MHz, CCl_3F) δ -91.0 (m, 2,6 position C_5F_4N, 2F), -158.3 (m, 3,5 position C_5F_4N, 2F).

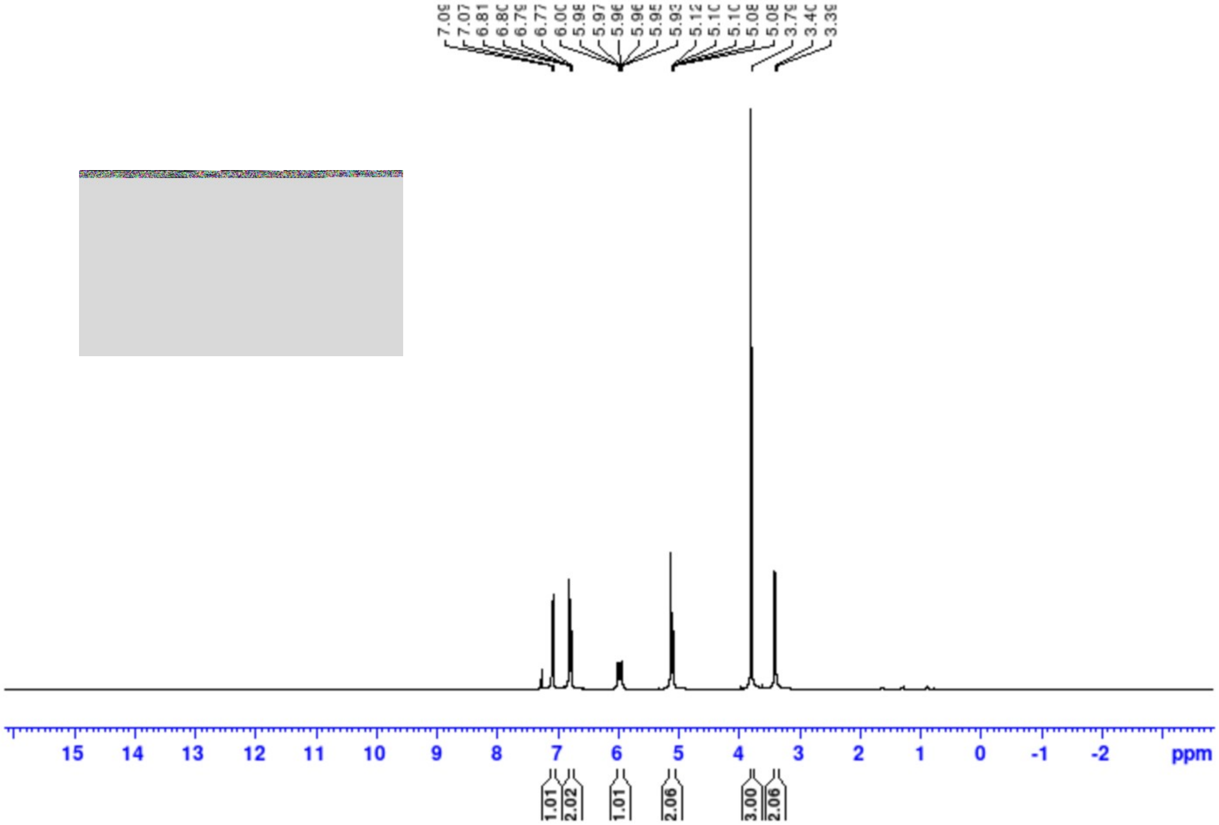

Figure S31. ^{1}H NMR of **28**.

^{1}H NMR ($CDCl_3$, 400 MHz) d 7.08 (d, -O-Ar (position 5)-, ^3J = 8.12 Hz, 1H), 6.80 (d, -O-Ar (position 3)-, ^4J = 1.36 Hz, 1H), 6.77 (dd, -O-Ar (position 6)-,^3J = 8.10 Hz, ^4J = 2.01 Hz, 1H), 5.97 (m, -CH_2-**CH**=CH_2, 1H), 5.10 (m, -CH=**CH_2**, 2H), 3.79 (s, -O-CH_3, 3H), 3.40 (d, -Ar-CH_2-CH=, ^3J = 6.56 Hz, 2H).

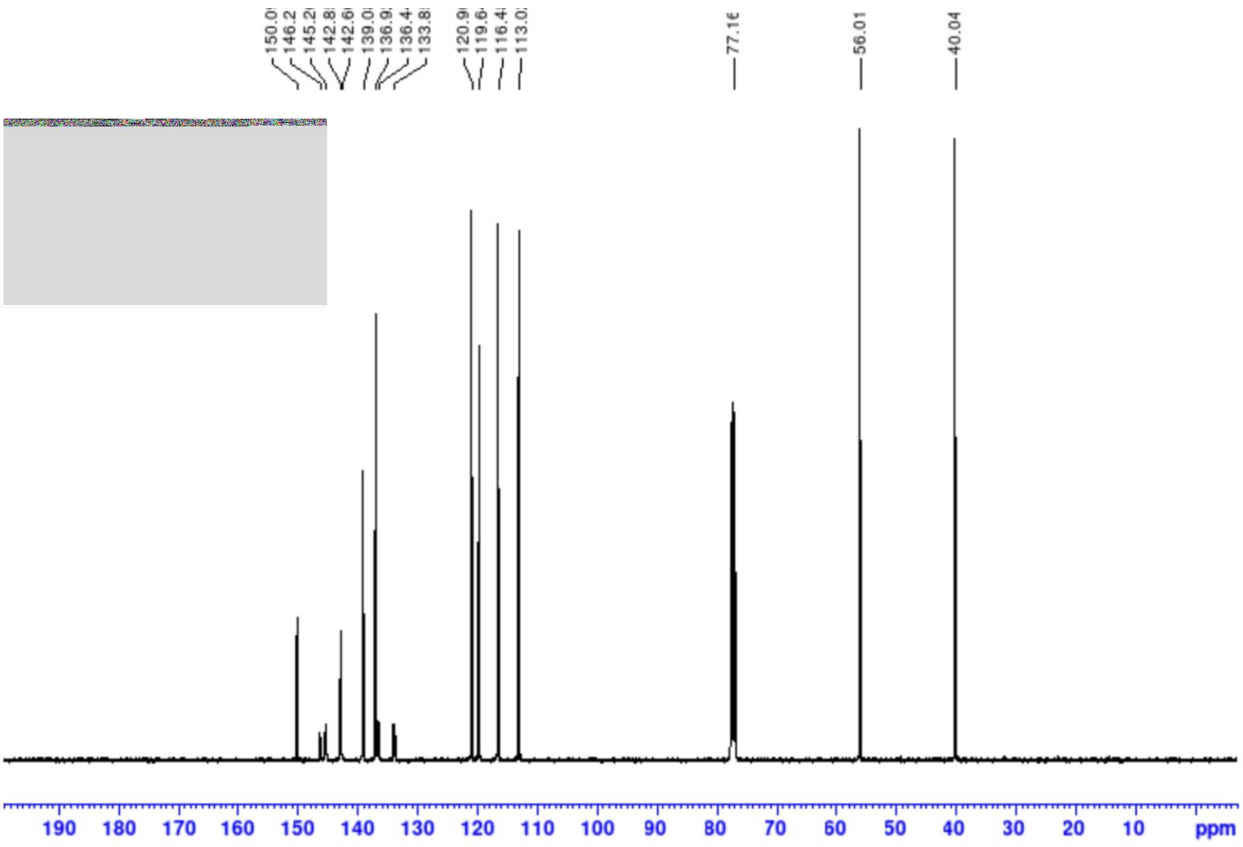

Figure S32. ^{13}C NMR of **28**.

^{13}C NMR ($CDCl_3$, 100MHz) d 150.1 (s, -O-Ar(1 position)-O-CH_3), 146.2 (m, 4 position C_5F_4N), 144.0 (dm, 3,5 position C_5F_4N, ^{1}J = 242 Hz), 142.6 (s, -O-Ar(2 position)-O-CH_3), 139.1 (s, -CH_2-**CH**=CH_2), 136.9 (s, -O-Ar(4 position)-O-CH_3), 135.1 (dm, 2,6 position C_5F_4N, ^{1}J = 260 Hz), 120.9 (s, -O-Ar(6 position)-O-CH_3), 119.6 (s, -O-Ar(5 position)-O-CH_3), 116.5 (s, -CH=**CH_2**), 113.0 (s, -O-Ar(3 position) -O-CH_3), 56.0 (s, -O-**CH_3**), 40.0 (s, -Ar-**CH_2**-CH=CH_2).

ATR-FTIR

Prepolymers:

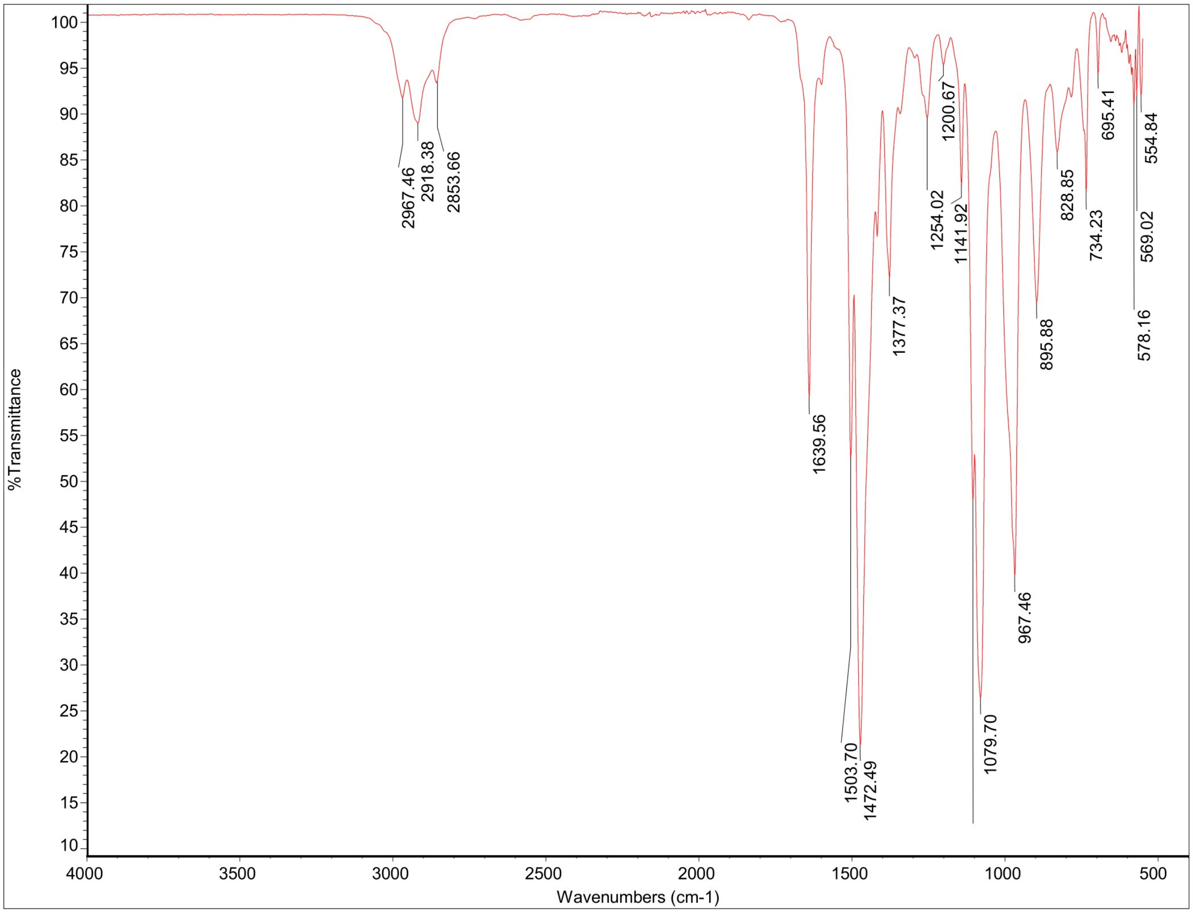

Figure S33: ATR-FTIR of **25**.

(ATR, cm^{-1}): 2968 (m, sp^3 C-H), 2918 (m, sp^3 C-H), 2854 (m, sp^3 C-H), 1640 (m, PFP C=N stretching) , 1504, 1472 (s, PFP C=N stretching), 1377 (m, CH_3 bend), 1254, 1201, 1142, 1080 (s, PFP C-F stretch), 967 (s, C-O stretch), 896 (s, PFP aromatic out-of-plane bend), 829, 734 (alkene C=C out-of-plane), 695.

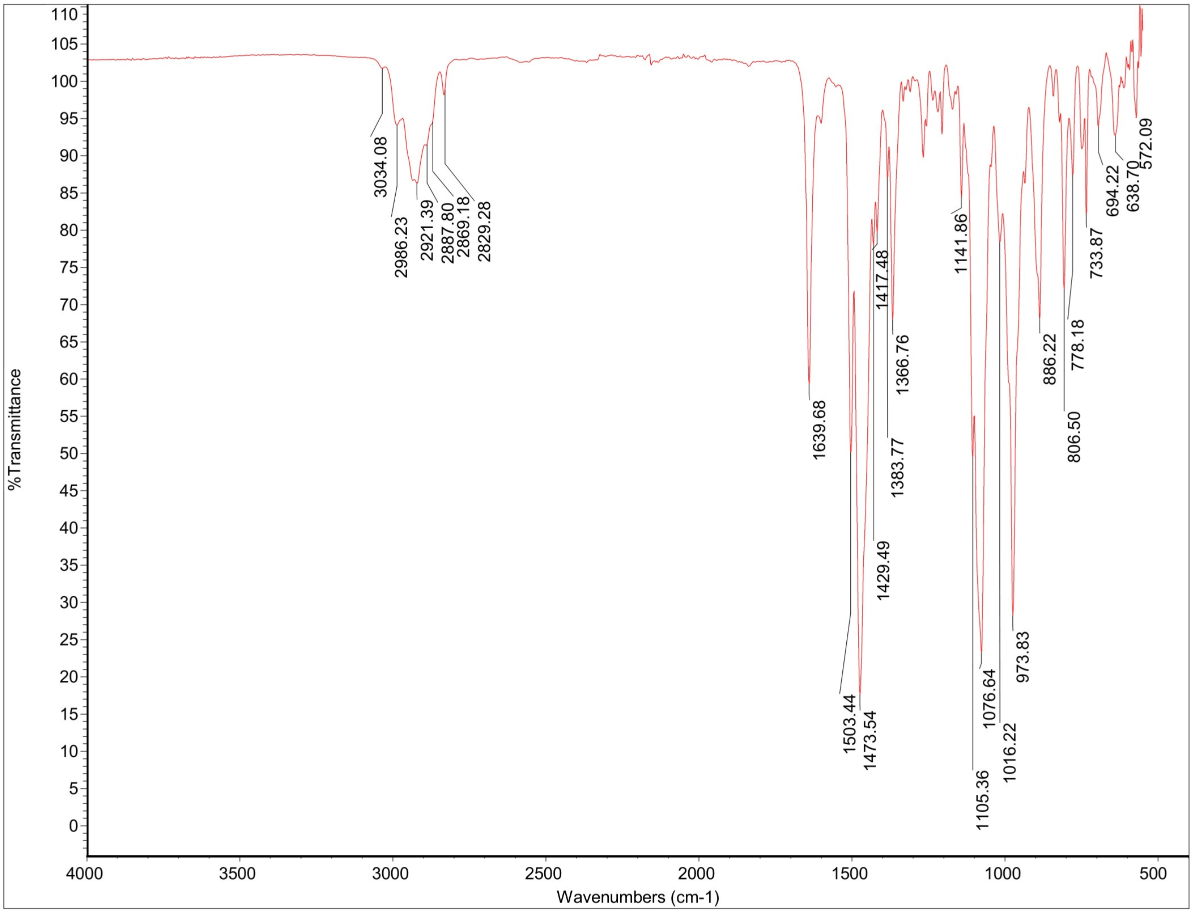

Figure S34: ATR-FTIR of **26**.

(ATR, cm^{-1}): 3034 (w, sp^2 C-H stretch), 2986 (m, sp^3 C-H stretch), 2921 (m, sp^3 C-H stretch), 2888 (m, sp^3 C-H stretch), 2869 (m, sp^3 C-H stretch), 2829 (w, sp^3 C-H stretch), 1640 (m, PFP C=N stretch), 1503, 1474 (m, PFP C=N stretch), 1430, 1418, 1367 (m, CH_3 bend), 1142, 1105, 1077 (s, C-F stretch), 1016, 974 (s, C-O stretch), 886 (m, PFP aromatic out-of-plane bend), 806 (m, C=C out-of-plane bend), 778, 734, 694, 639, 572.

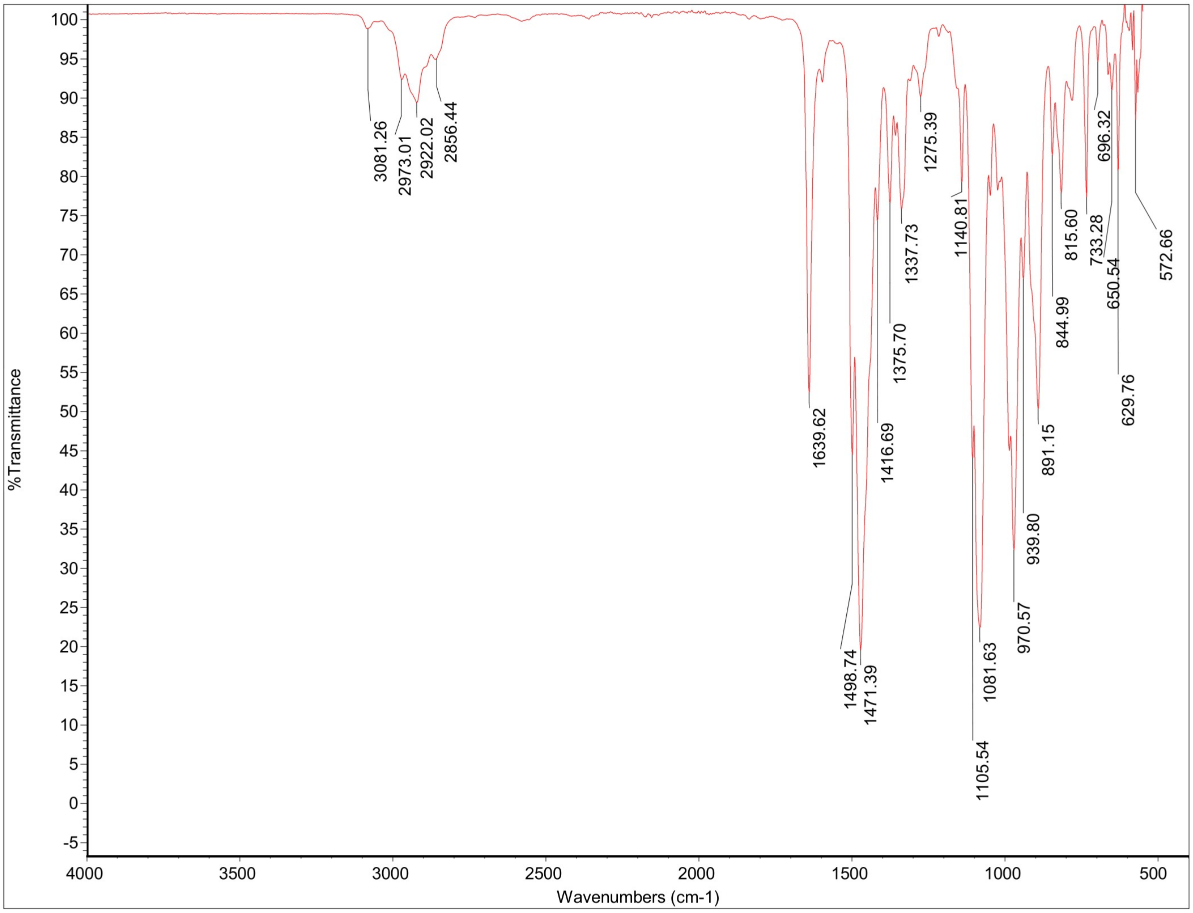

Figure S35: ATR-FTIR of **27**.

(ATR, cm^{-1}): 3081 (w, sp^2 C-H stretch), 2973 (m, sp^3 C-H stretch), 2922 (m, sp^3 C-H stretch), 2856 (w, sp^3 C-H stretch), 1640 (m, PFP C=N stretch), 1499, 1471 (s, PFP C=N stretch), 1417, 1375 (m, CH_3 bend) , 1338, 1275, 1141, 1106, 1082 (s, PFP C-F stretch), 972 (s, C-O stretch), 940, 891 (m, PFP aromatic out-of-plane bend), 845, 816 (m, alkene out-of-plane bend), 733, 699, 650, 630, 573.

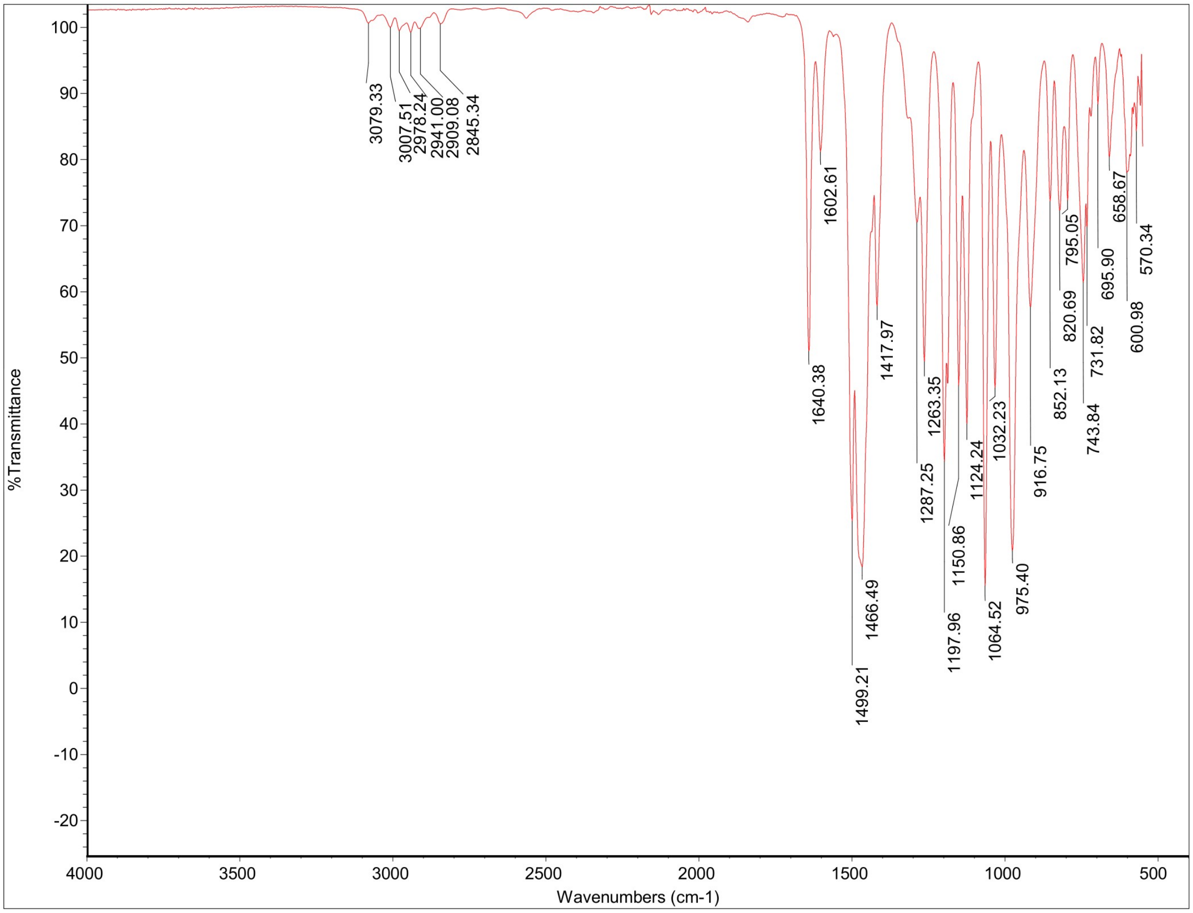

Figure S36: ATR-FTIR of **28**.

(ATR, cm^{-1}): 3079 (w, Aromatic sp^2 C-H stretch), 3008 (w, sp^2 C-H stretch), 2978 (w, sp^3 C-H stretch), 2941 (w, sp^3 C-H stretch), 2909 (w, sp^3 C-H stretch), 2845 (w, sp^3 C-H stretch), 1640 (m, PFP C=N stretch), 1603 (m, Eugenol C=C stretch), 1499 (s, Eugenol Ar C=C stretch), 1466 (s, PFP C=N stretch), 1417, 1287, 1263, 1198, 1151, 1124, 1064 (s, PFP C-F stretch), 1032 (s, Eugenol vinylic C=C bend), 975 (s, C-O stretch), 917 (m, Eugenol vinylic C=C bend), 852 (m, Eugenol out-of-plane bend), 821, 795, 744 (m, Eugenol out-of-plane bend), 732, 596, 659, 601, 570.

Polymers:

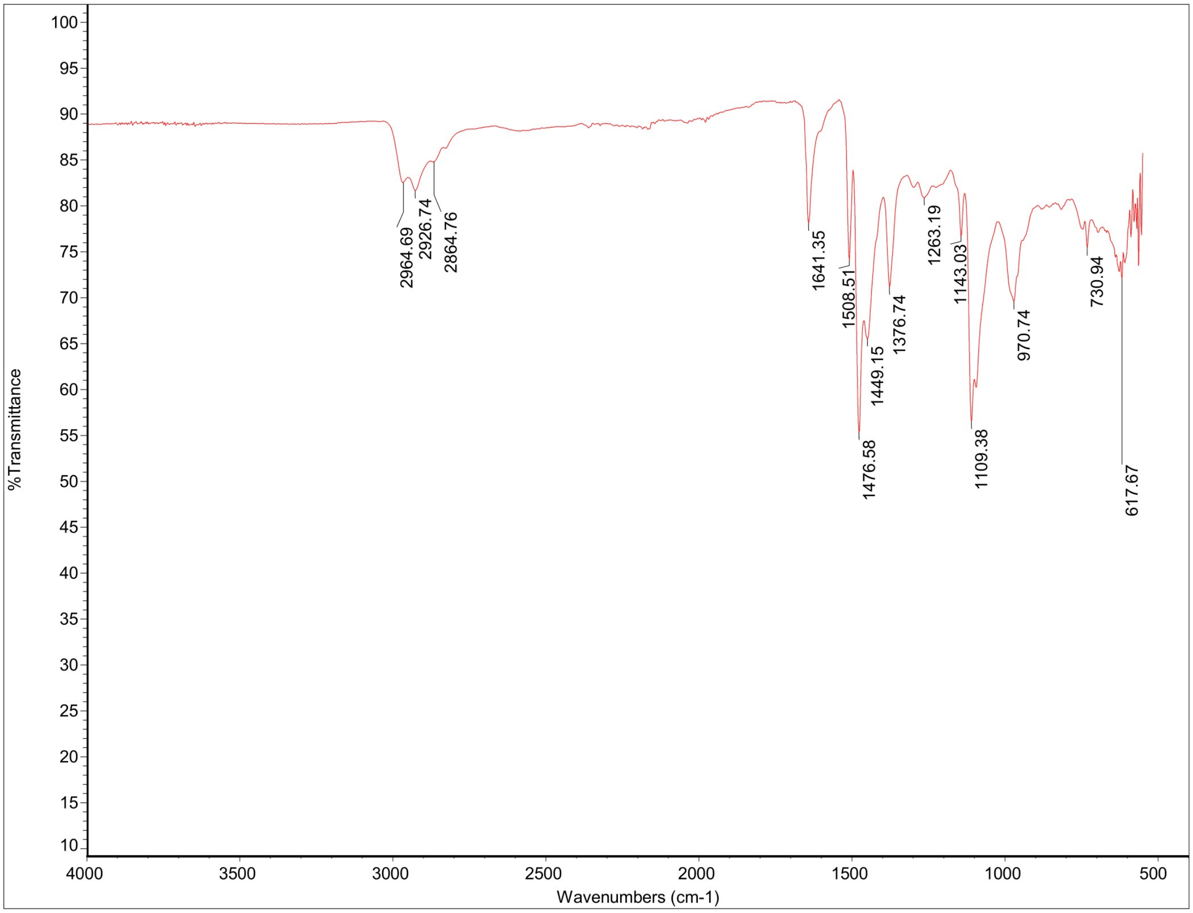

Figure S37: ATR-FTIR of **25-S_2Cl_2**.

(ATR, cm^{-1}): 2964 (sp^3 C-H stretch), 2927 (m, sp^3 C-H stretch), 2865 (m, sp^3 C-H stretch), 1641 (m, PFP C=N stretch), 1508, 1477 (s, PFP C=N stretch), 1449, 1377 (m, CH_3 bend), 1263, 1143, 1109 (s, PFP C-F stretch), 971 (m, C-O stretch), 731, 618 (m, C-Cl stretch).

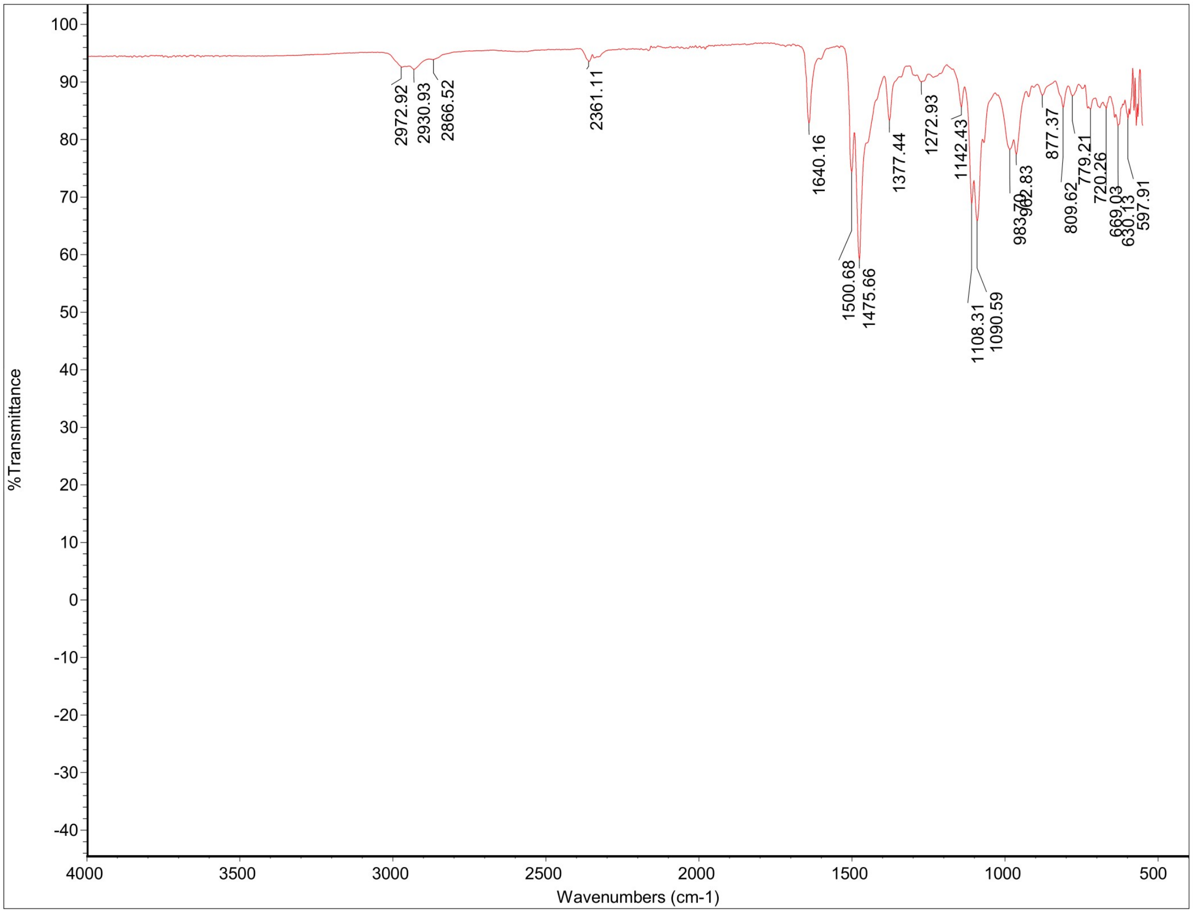

Figure S38: ATR-FTIR of **27-S_2Cl_2**.

(ATR, cm^{-1}): 2973 (w, sp^3 C-H stretch), 2931 (w, sp^3 C-H stretch), 2866 (w, sp^3 C-H stretch), 1640 (m, PFP C=N stretch), 1501, 1476 (m, PFP C=N stretch), 1377 (m, CH_3 bend), 1273, 1142, 1108 (m, PFP C-F stretch), 1090 (m, C-O stretch), 984 (m, PFP ring out-of-plane bend), 963, 877, 908, 779, 720, 669 (w, C-Cl stretch), 630, 598.

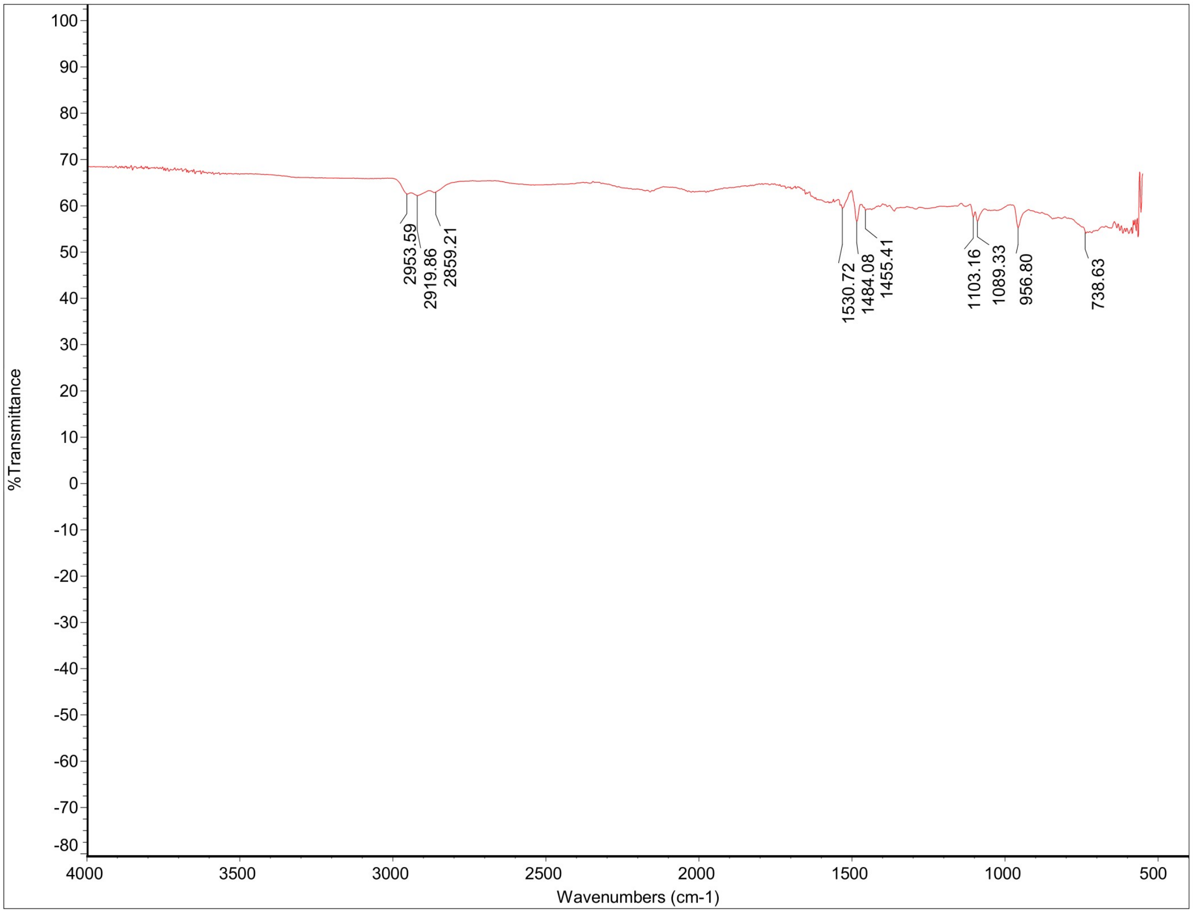

Figure S39: ATR-FTIR of **25-S_8**.

(ATR, cm^{-1}): 2954 (w, sp^3 C-H stretch), 2920 (w, sp^3 C-H stretch), 2859 (w, sp^3 C-H stretch), 1531, 1484, 1455 (w, CH_2 bend), 1103, 1089 (w, PFP C-F stretch), 957 (w, C-O stretch), 739.

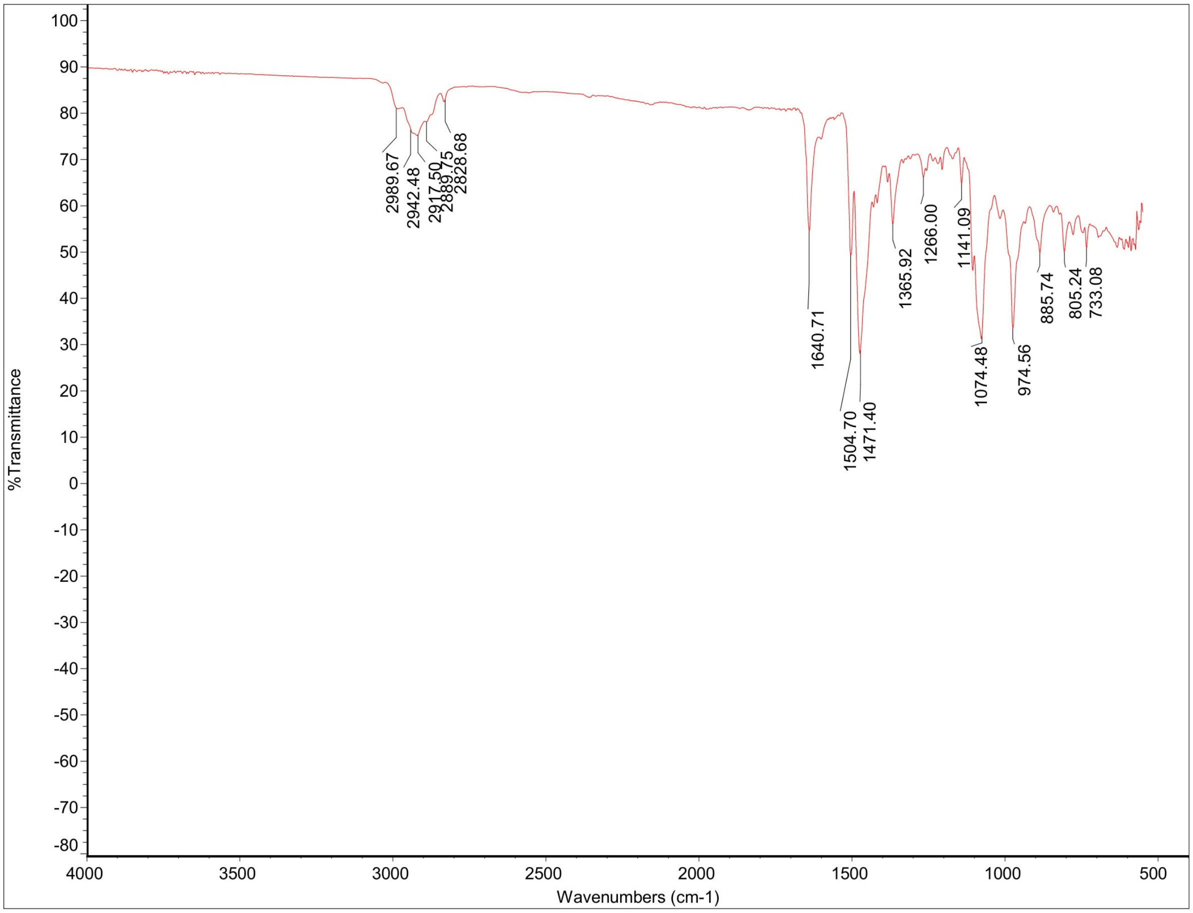

Figure S40: ATR-FTIR of **26-S$_8$**.

(ATR, cm^{-1}): 2990 (w, sp^3 C-H stretch), 2942 (m, sp^3 C-H stretch), 2918 (m, sp^3 C-H stretch), 2890 (m, sp^3 C-H stretch), 2829 (w, sp^3 C-H stretch), 1641 (m, PFP C=N stretch), 1505, 1471 (s, PFP C=N stretch), 1366 (m, CH_3 bend), 1266, 1141, 1074 (s, PFP C-F stretch), 975 (s, C-O stretch), 886 (w, PFP ring out-of-plane bend), 805, 733.

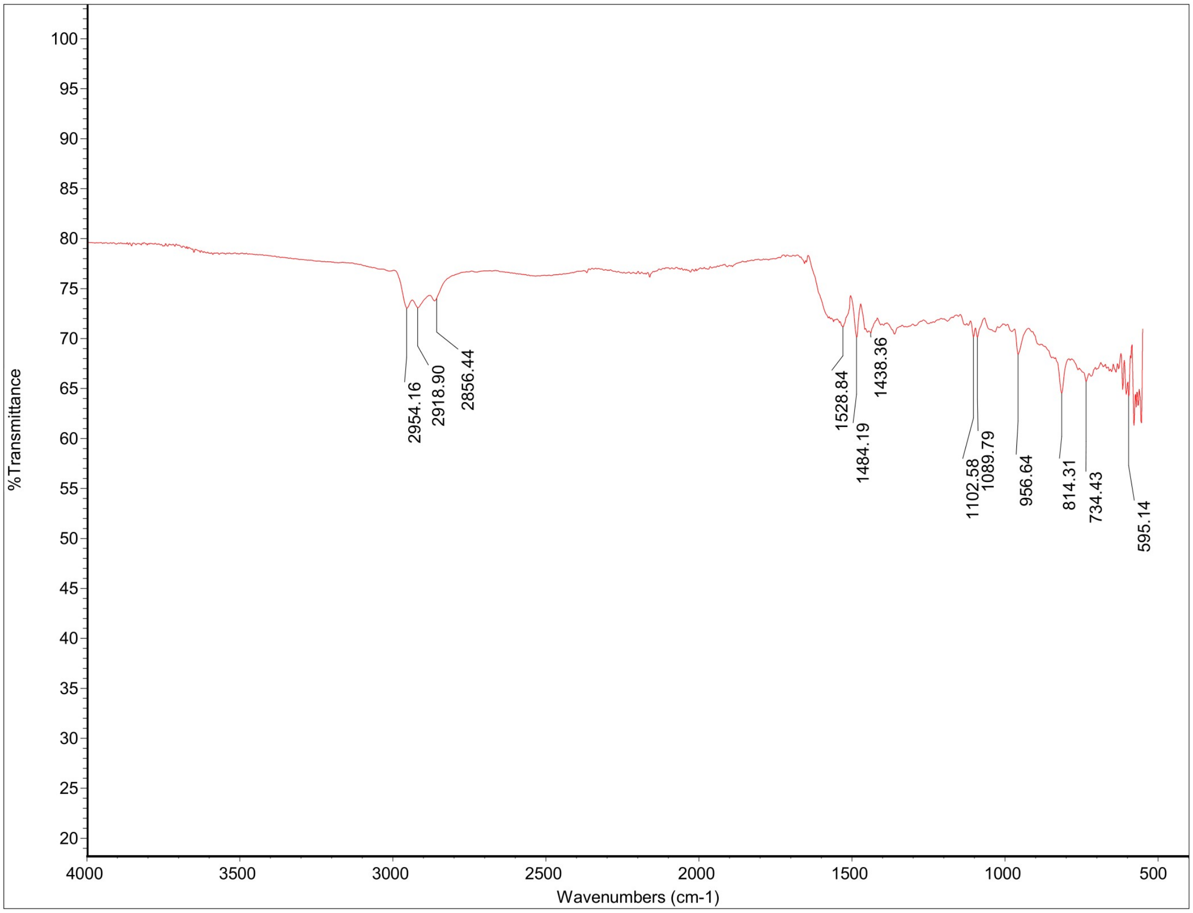

Figure S41: ATR-FTIR of **27-S_8**.

(ATR, cm^{-1}): 2954 (w, sp^3 C-H stretch), 2919 (w, sp^3 C-H stretch), 2856 (w, sp^3 C-H stretch), 1529, 1484, 1438, 1102, 1090 (w, PFP C-F stretch), 957 (w, C-O stretch), 814 (w, PFP ring out-of-plane bend), 734, 595.

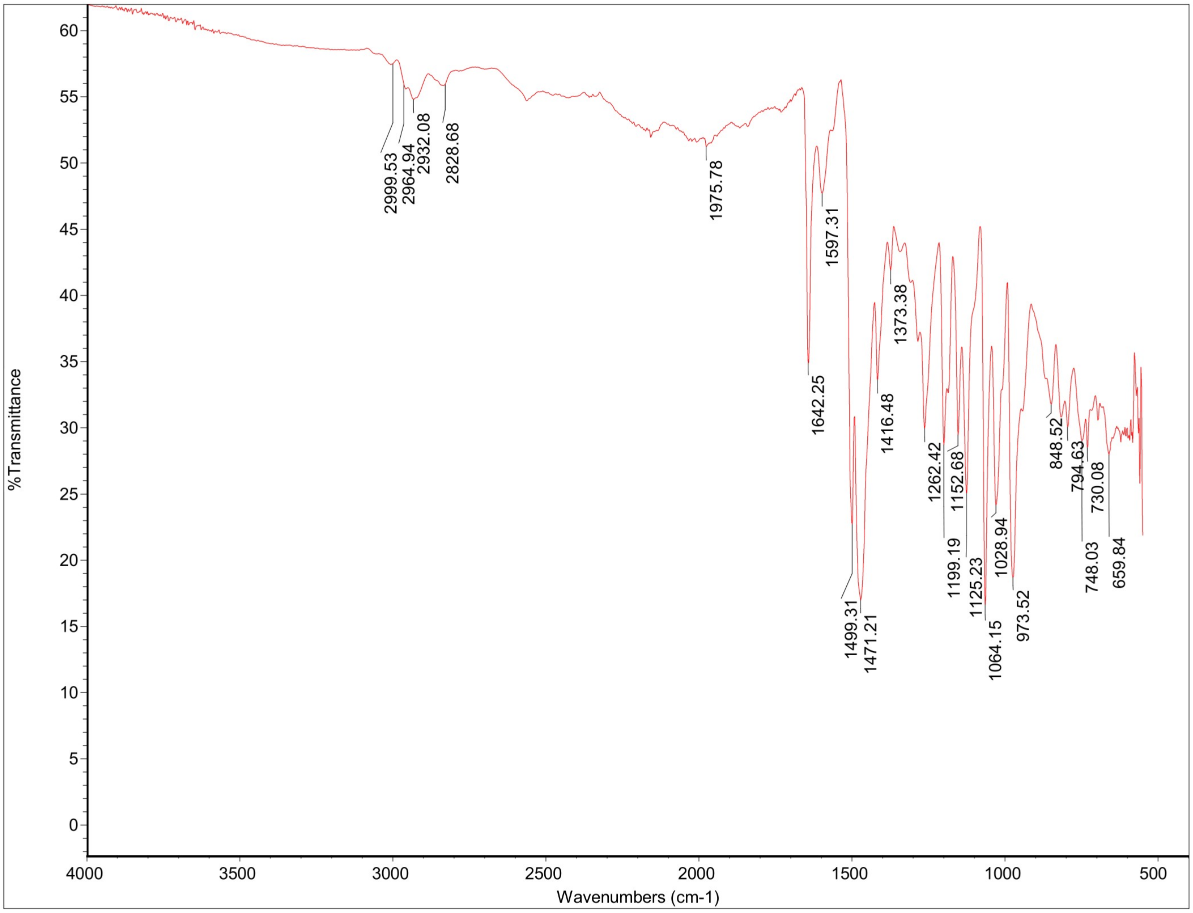

Figure S42: ATR-FTIR of **28-S₈**.

(ATR, cm^{-1}): 3000 (w, Ar sp^2 C-H stretch), 2965 (w, sp^3 C-H stretch), 2932 (w, sp^3 C-H stretch), 2828 (w, sp^3 C-H stretch), 1642 (m, PFP C=N stretch), 1597 (w, Eugenol C=C stretch), 1499 (s, Eugenol C=C stretch), 1471 (s, PFP C=N stretch), 1416, 1373, 1262, 1199, 1125, 1064 (s, PFP C-F stretch), 1029, 974 (s, C-O stretch), 848 (w, Eugenol out-of-plane bend), 794 (w, PFP out-of-plane bend), 748 (w, Eugenol out-of-plane bend), 730, 660.

Mass Spectroscopy:

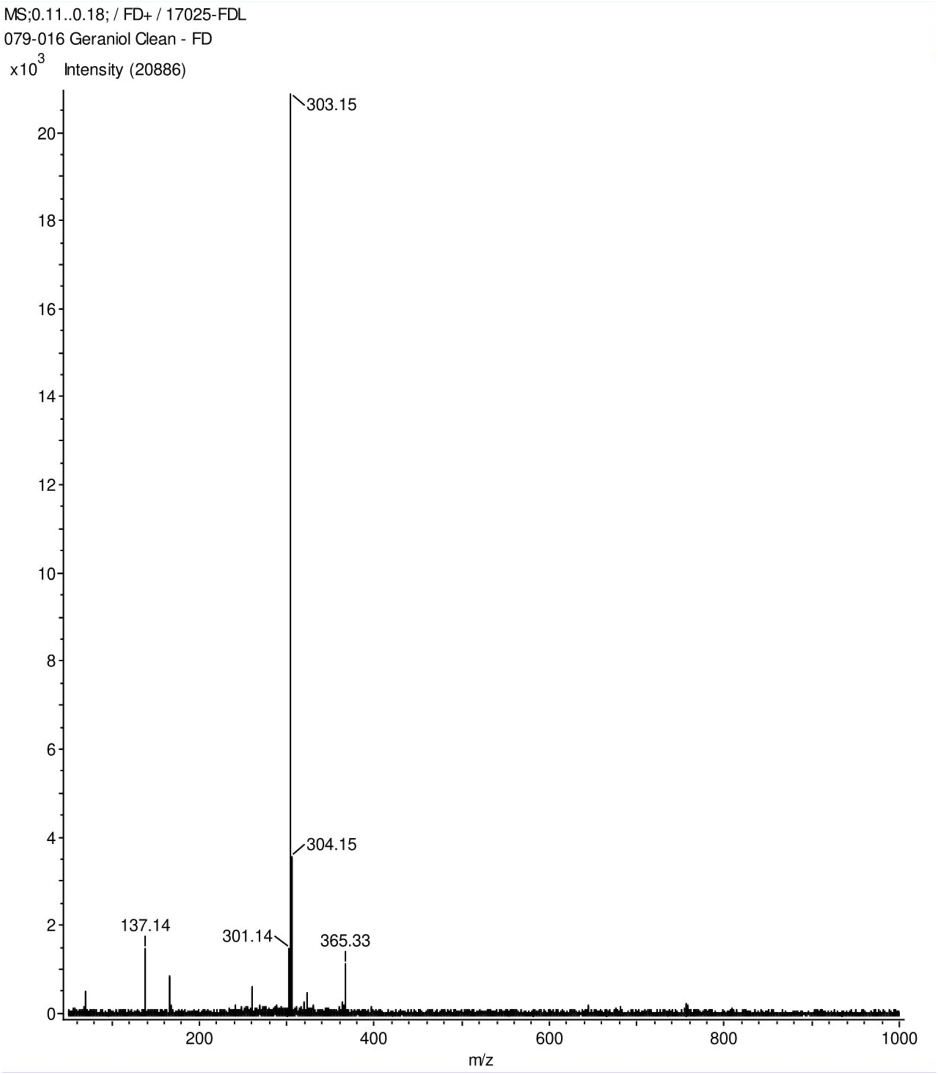

Figure S43: FD-MS spectrum of **25**.

FD-MS m/z: 303.15 (100.00%), 304.15 (17.66%).

Calc. m/z: 303.12 (100.0%), 304.13 (16.5%), 305.13 (1.5%).

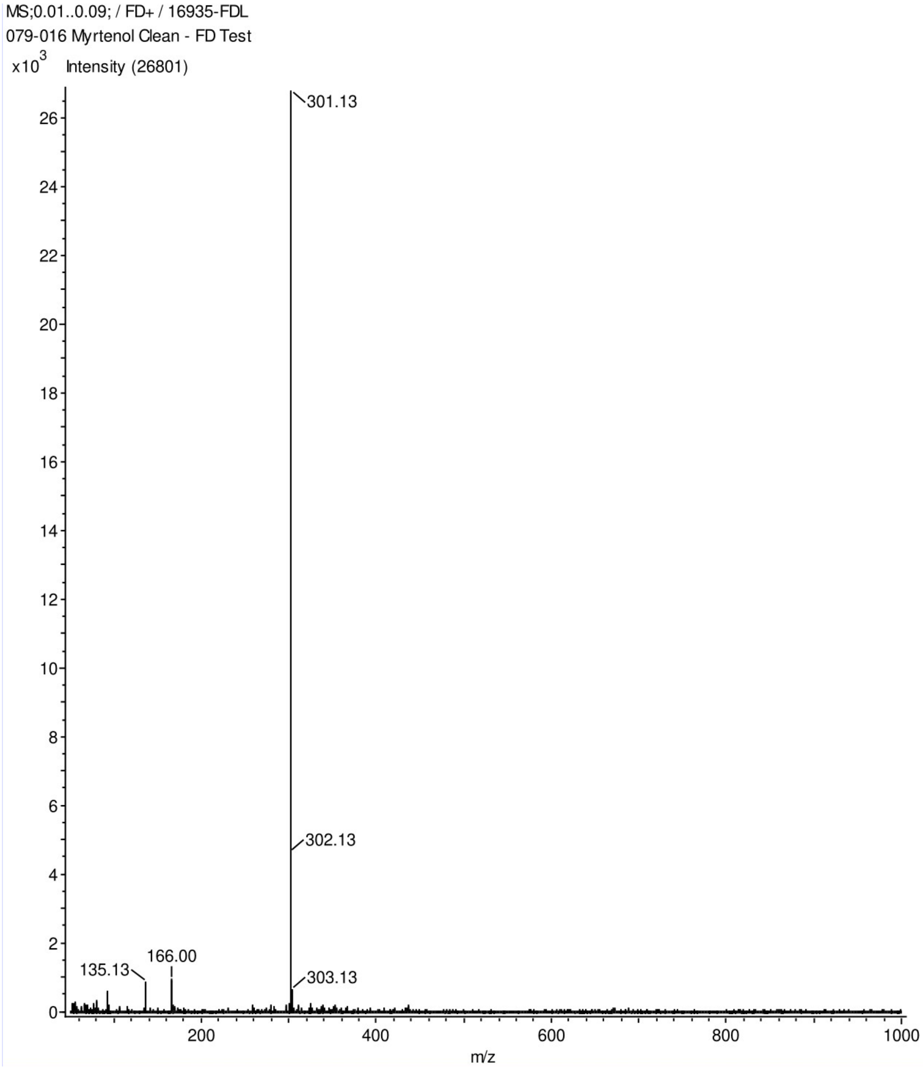

Figure S44: FD-MS spectrum of **26**.

FD-MS m/z: 301.13, 302.13, 303.13

Calc. m/z: 301.11, 302.11, 303.12

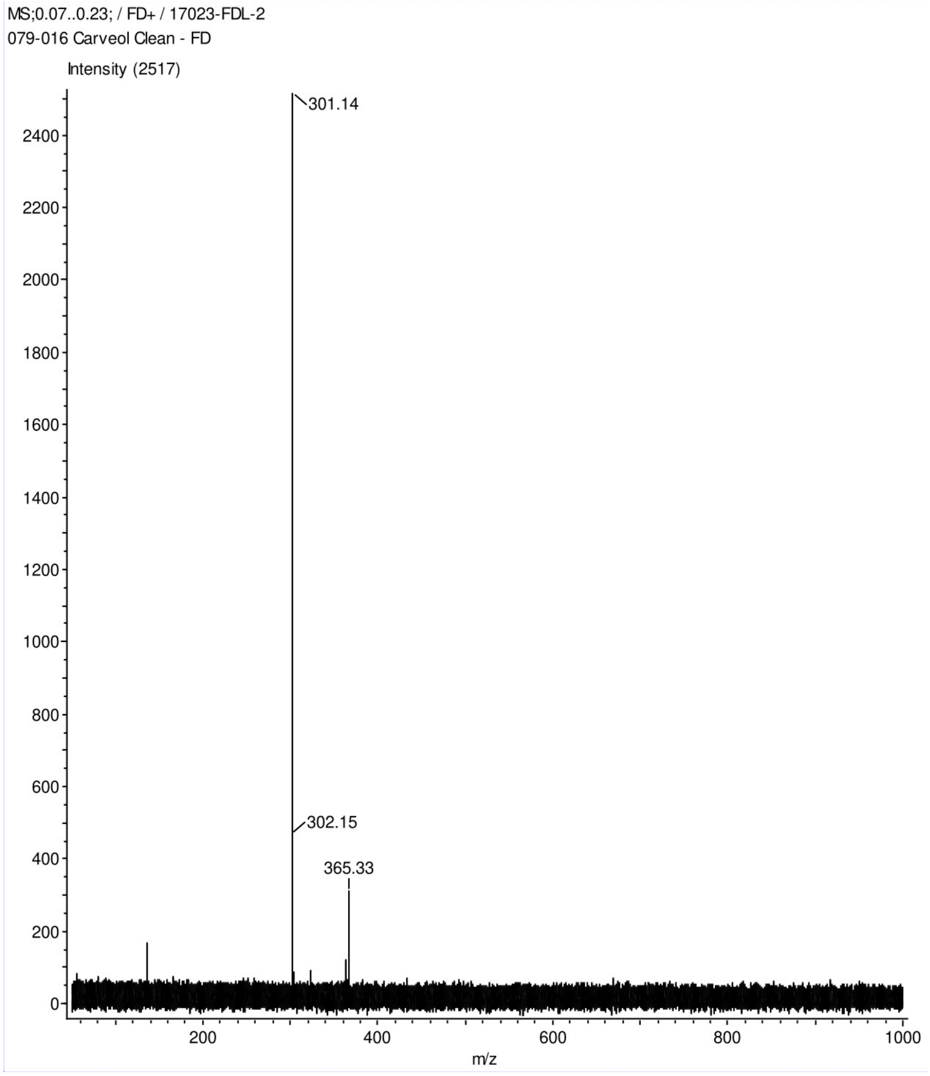

Figure S45: FD-MS spectrum of **27**.
FD-MS m/z: 301.14, 302.15

Calc. m/z 301.11, 302.11, 303.12

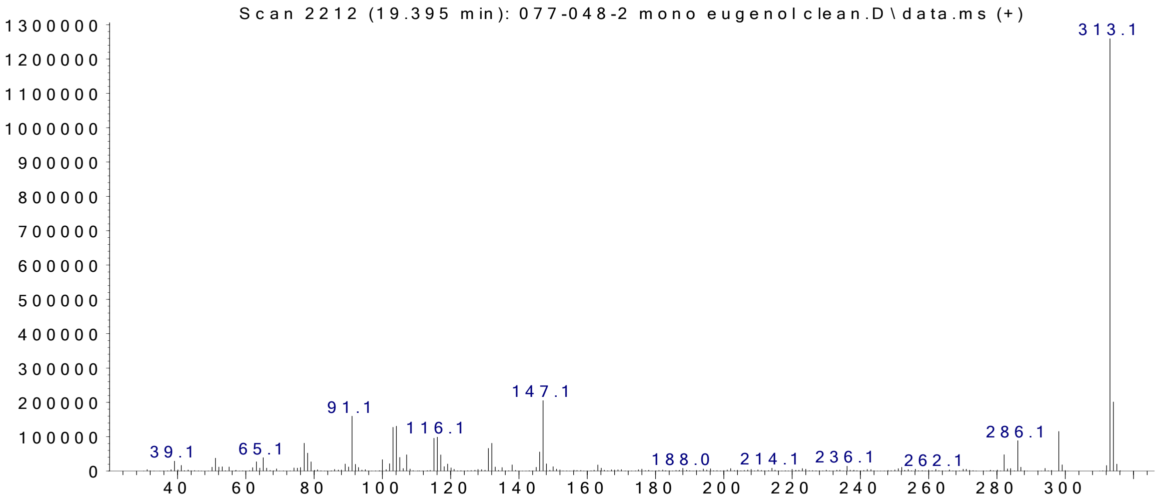

Figure S46: GC EI-MS spectrum of **28**.
GC-EI/MS m/z: 313.1 (100%), 314.1 (15.4%), 315.1 (1.6%)

Calc. m/z: 313.07 (100%), 314.08 (16.4%), 315.08 (1.7%)

Thermal Gravimetric Analysis (TGA):

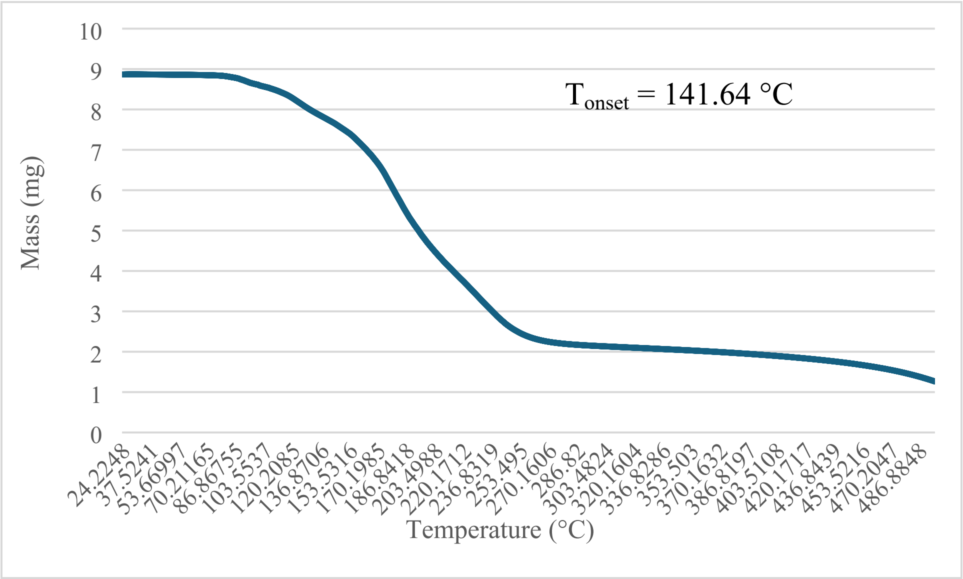

Figure S47: TGA under air of **25** inverse vulcanized with S_2Cl_2 at room temperature.

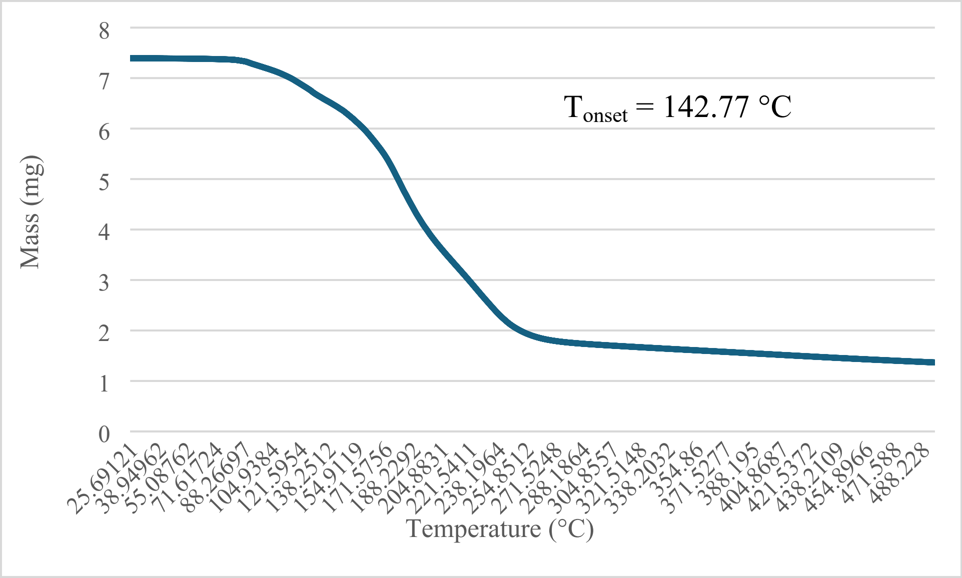

Figure S48: TGA under nitrogen of **25** inverse vulcanized with S_2Cl_2 at room temperature.

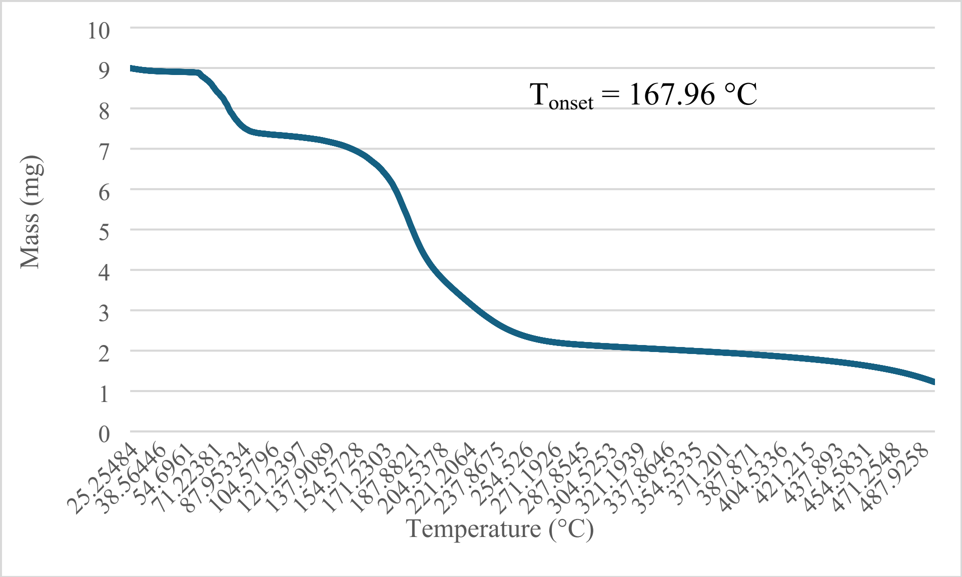

Figure S49: TGA under air of **25** inverse vulcanized with S_2Cl_2 at 65 °C.

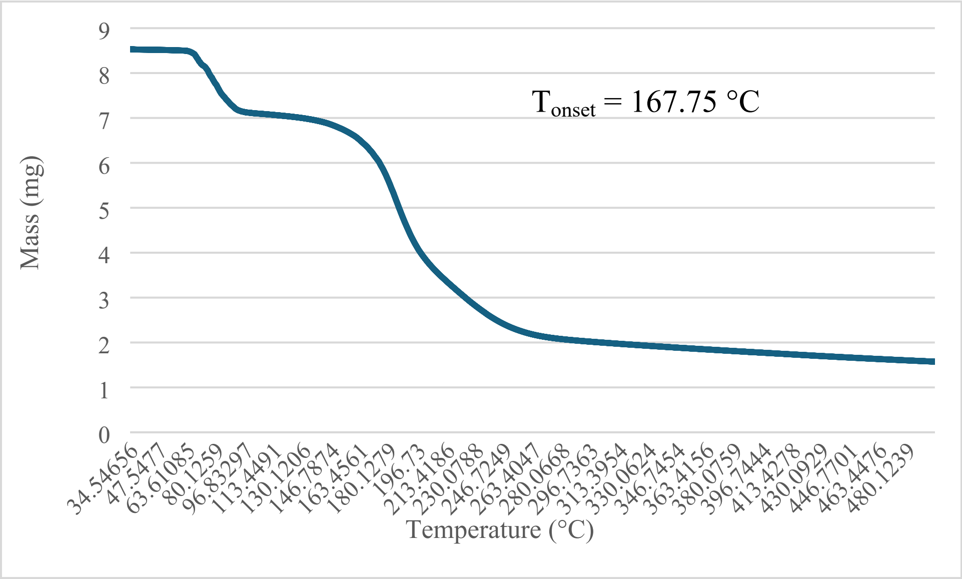

Figure S50: TGA under nitrogen of **25** inverse vulcanized with S_2Cl_2 at 65 °C.

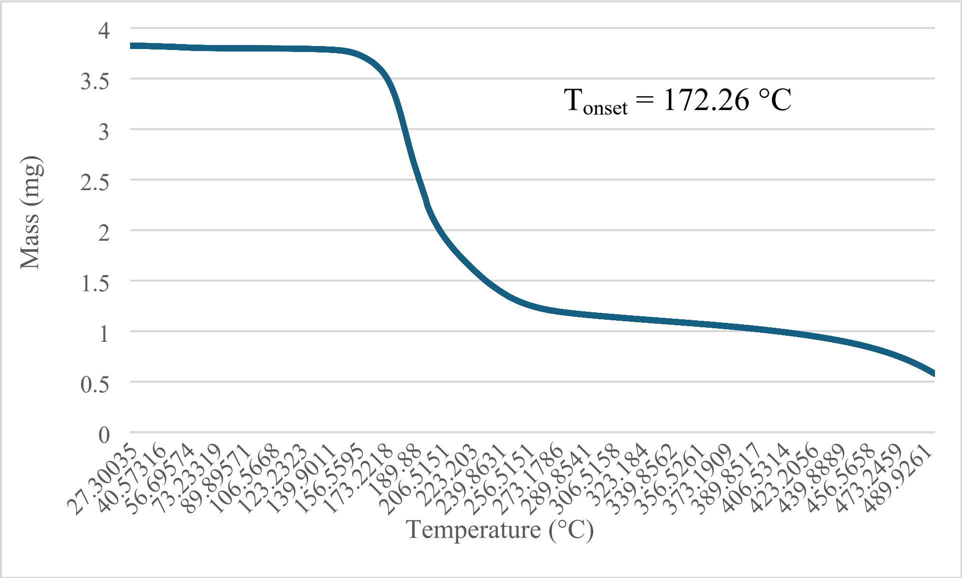

Figure S51: TGA under air of **25** inverse vulcanized with S_2Cl_2 at 70 °C in nitrogen atmosphere.

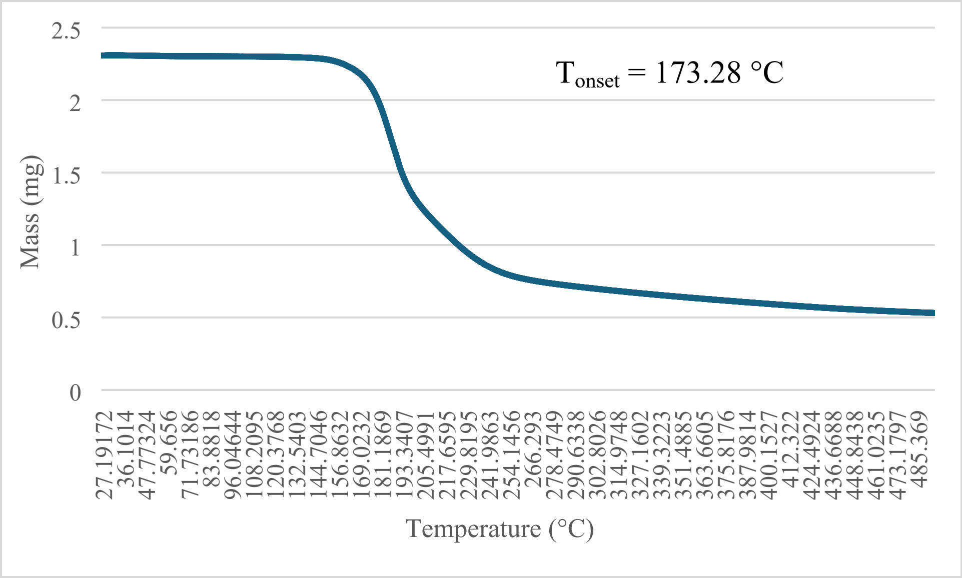

Figure S52: TGA under nitrogen of **25** inverse vulcanized with S_2Cl_2 at 70 °C in nitrogen atmosphere.

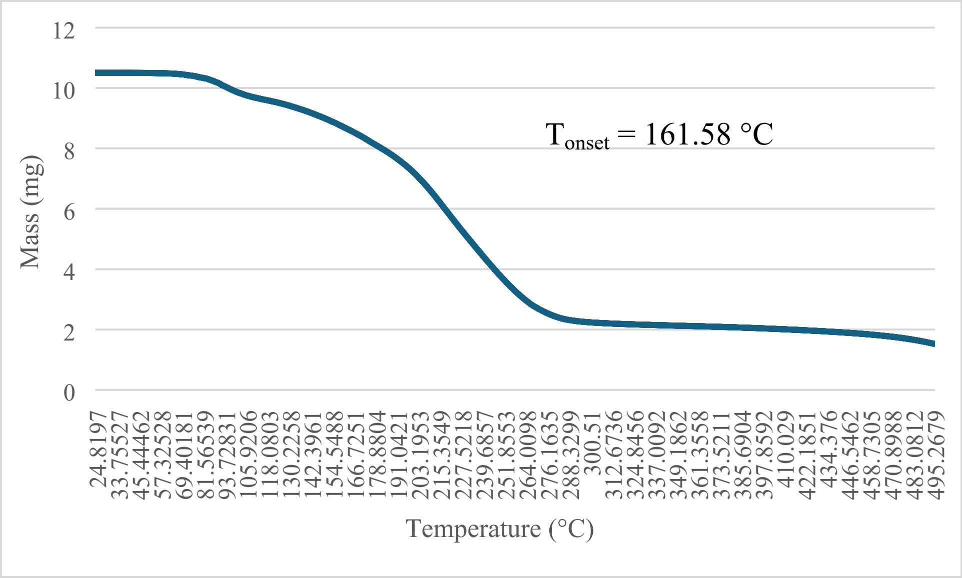

Figure S53: TGA under air of **27** inverse vulcanized with S_2Cl_2 at 70 °C.

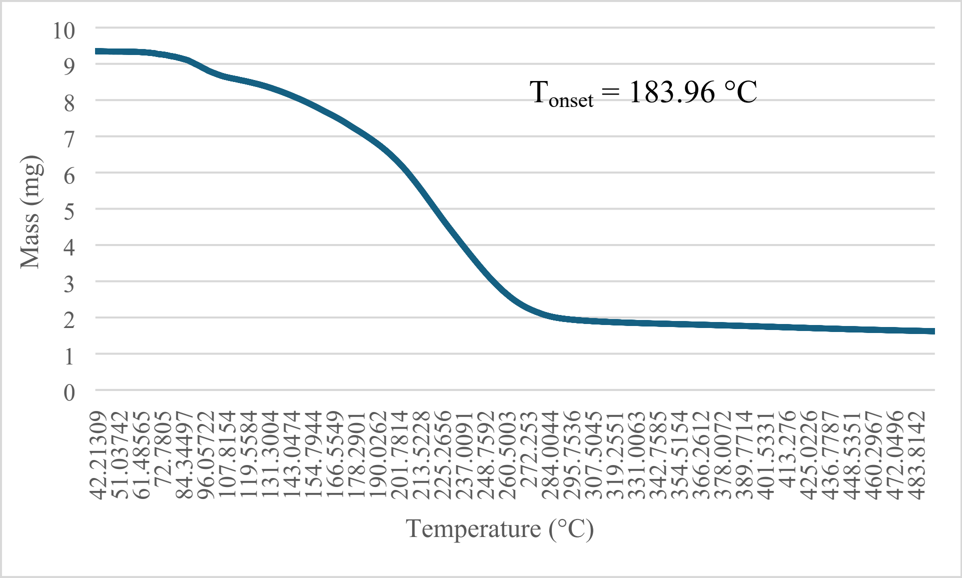

Figure S54: TGA under nitrogen of **27** inverse vulcanized with S_2Cl_2 at 70 °C.

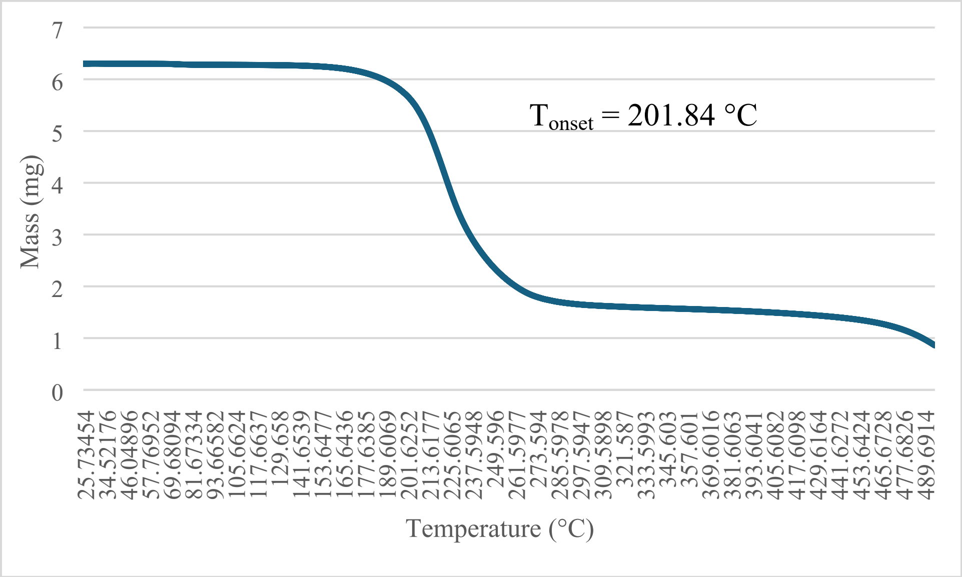

Figure S55: TGA under air of **27** inverse vulcanized with S_2Cl_2 at 70 °C under nitrogen.

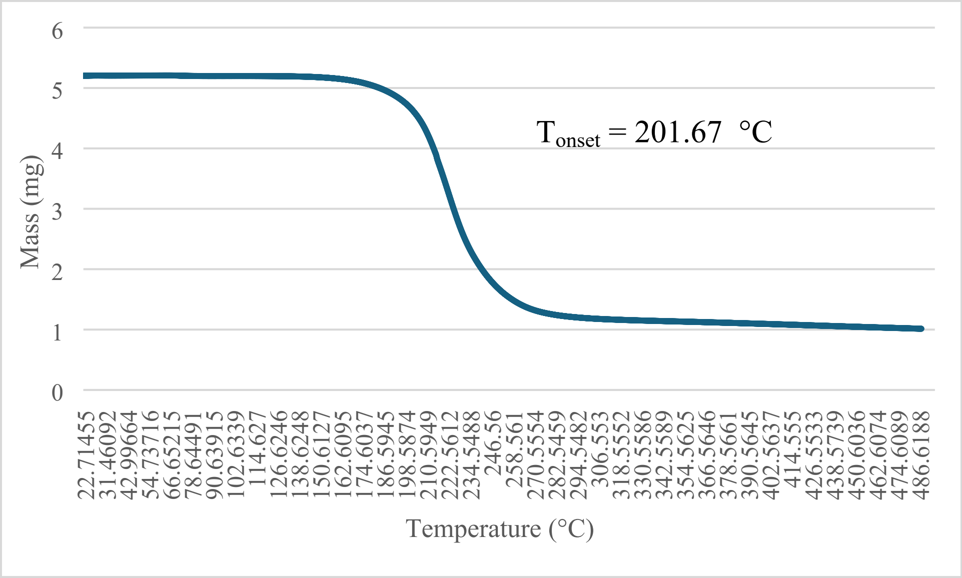

Figure S56: TGA under nitrogen of **27** inverse vulcanized with S_2Cl_2 at 70 °C under nitrogen.

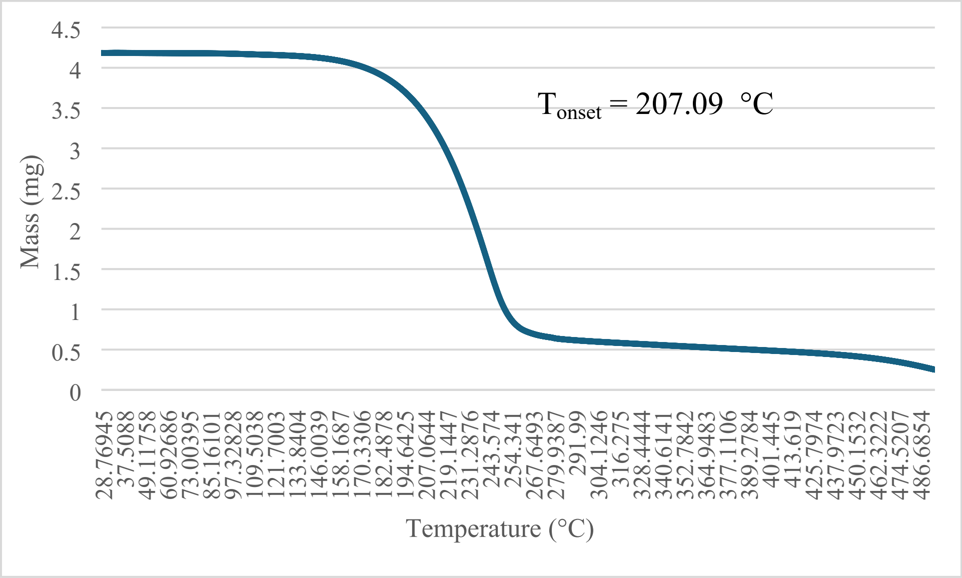

Figure S57: TGA under air of **25** inverse vulcanized with S_8.

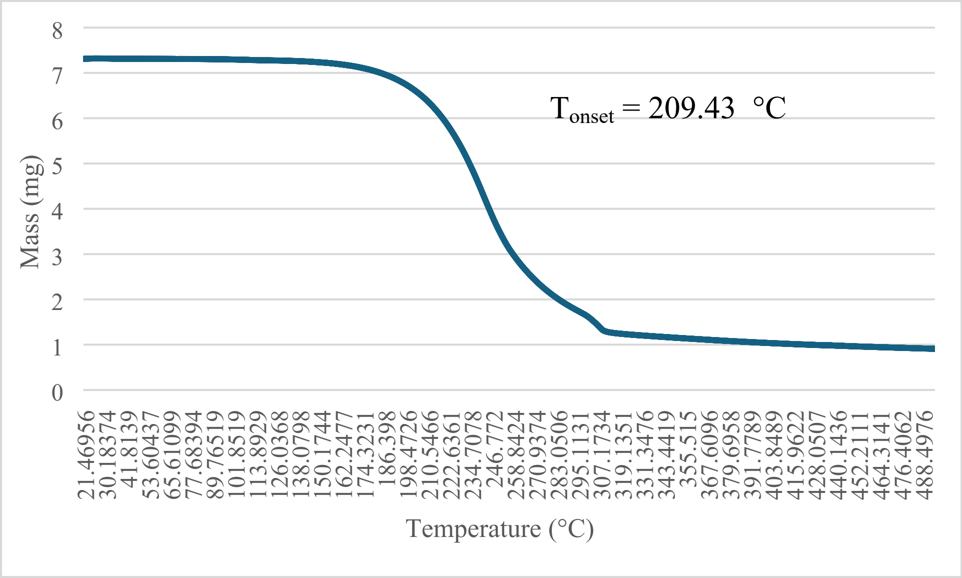

Figure S58: TGA under nitrogen of **25** inverse vulcanized with S_8.

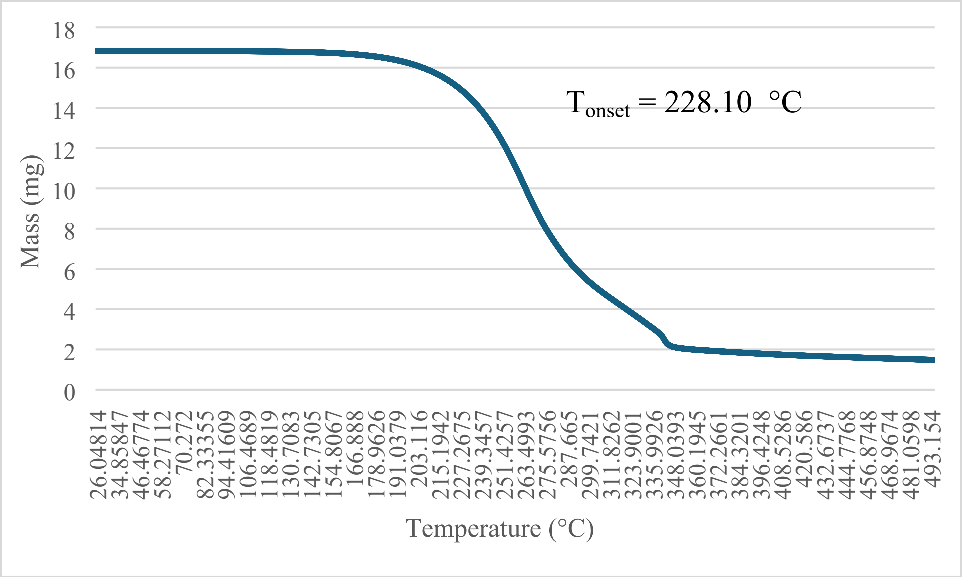

Figure S59: TGA under air of **26** inverse vulcanized with S_8.

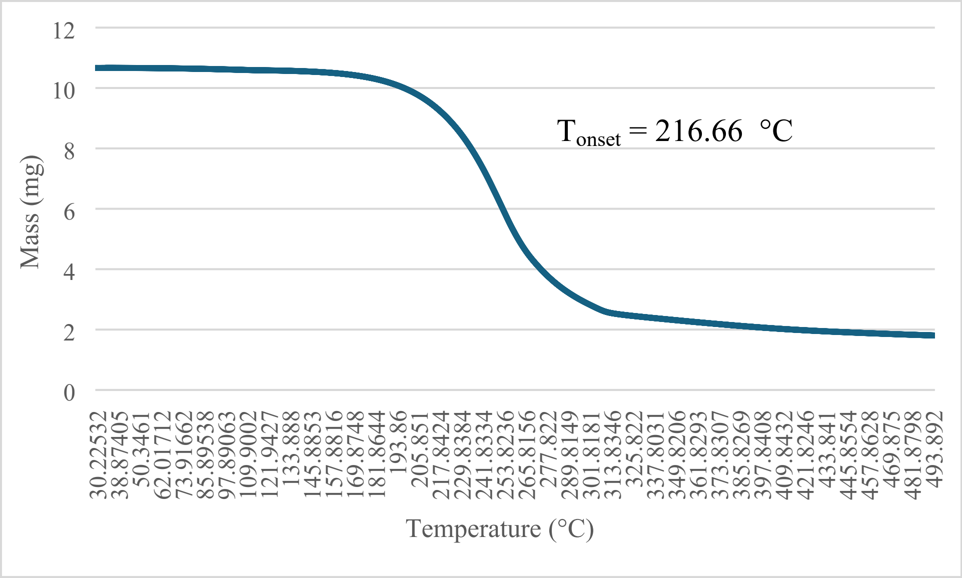

Figure S60: TGA under nitrogen of **26** inverse vulcanized with S_8.

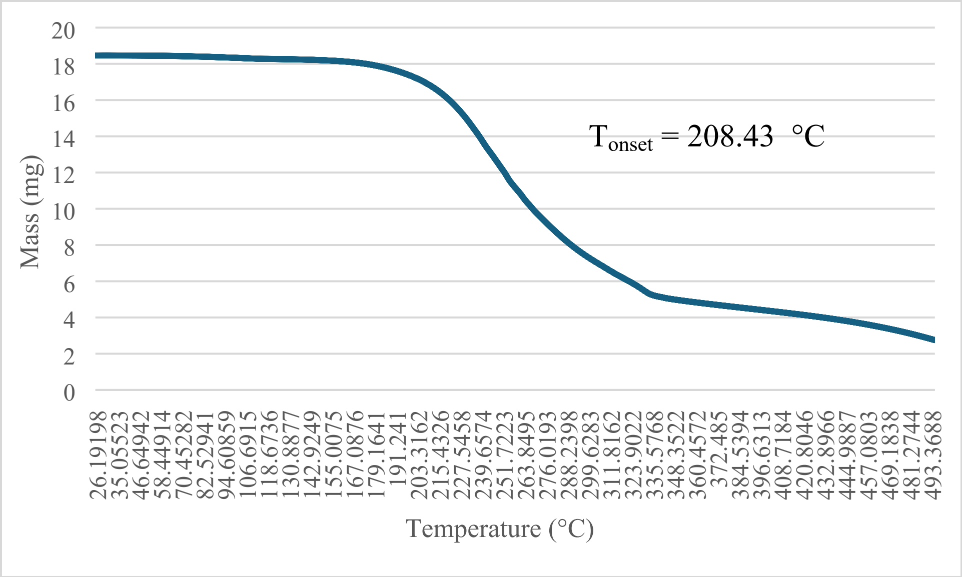

Figure S61: TGA under air of **27** inverse vulcanized with S_8.

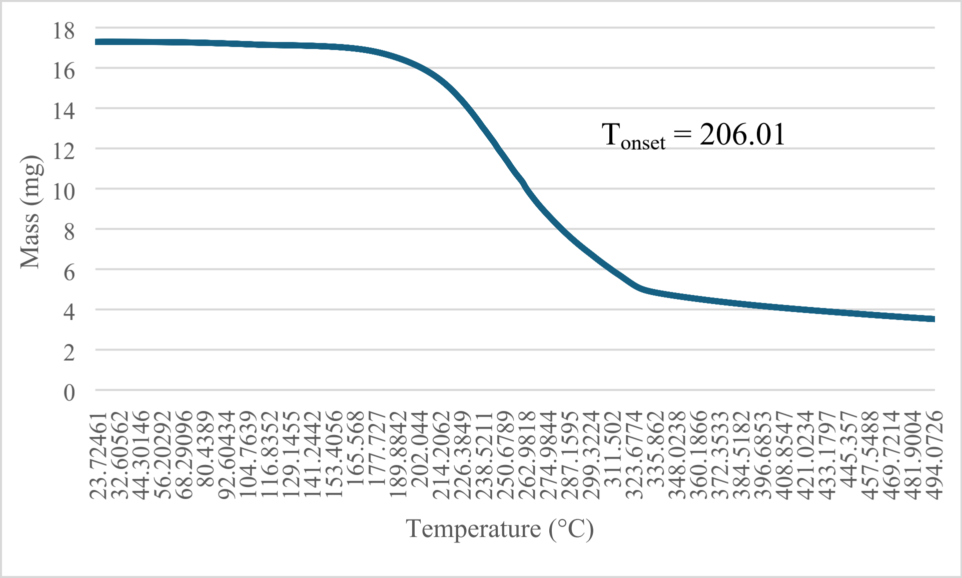

Figure S62: TGA under nitrogen of **27** inverse vulcanized with S_8.

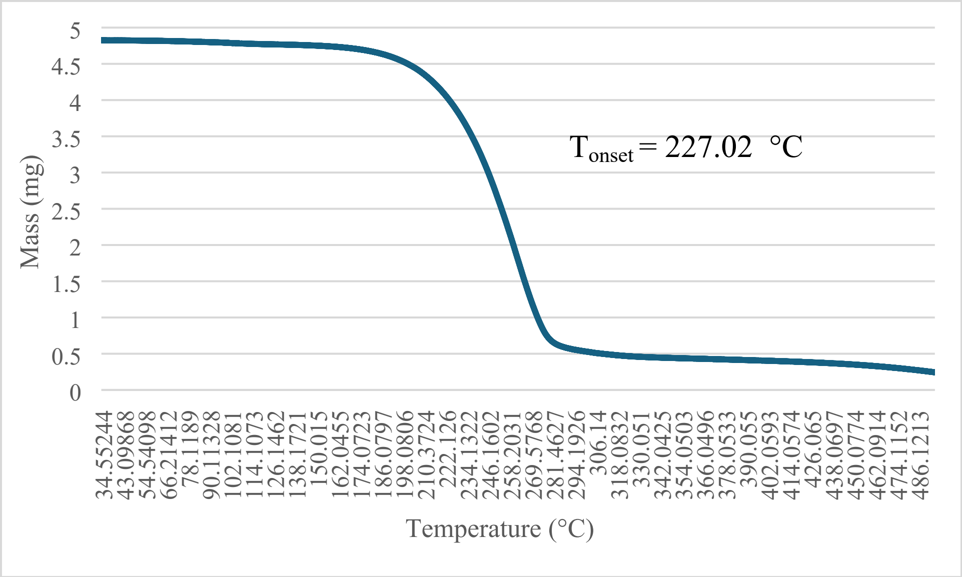

Figure S63: TGA under air of **28** inverse vulcanized with S_8.

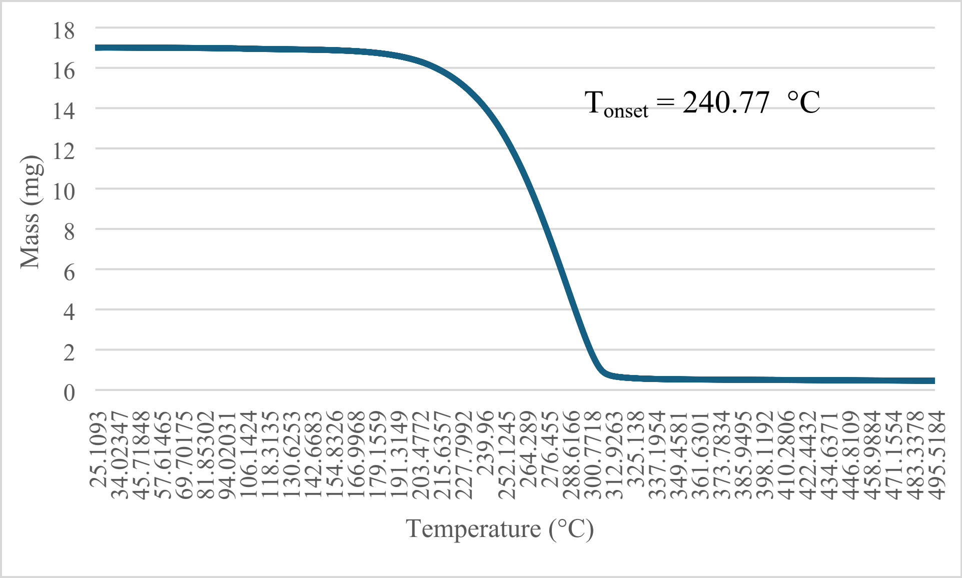

Figure S64: TGA under nitrogen of **28** inverse vulcanized with S_8.

Differential Scanning Calorimetry (DSC):

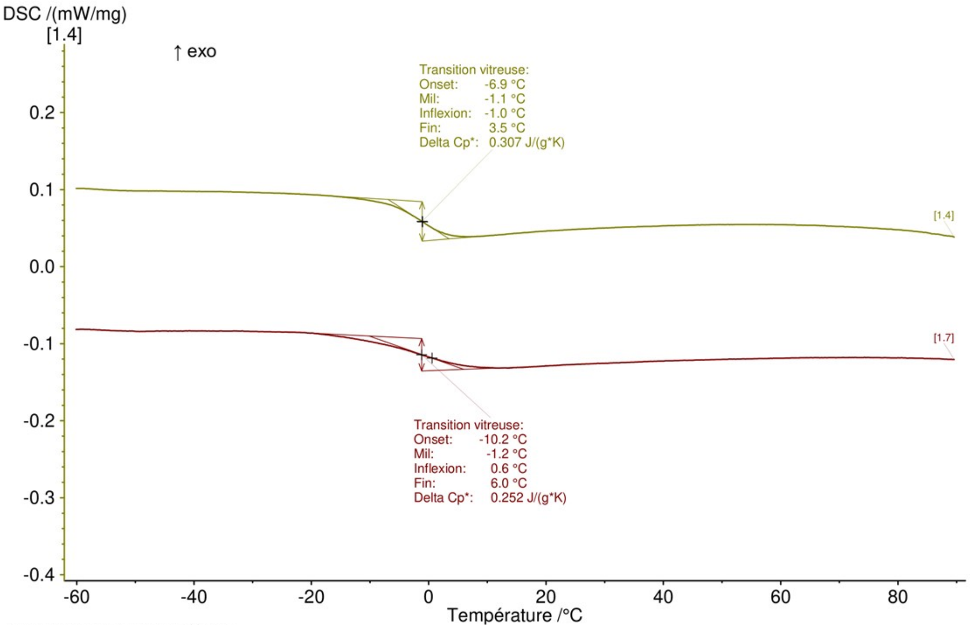

Figure S65: DSC of 25 inverse vulcanized with S_2Cl_2 at 20 °C.

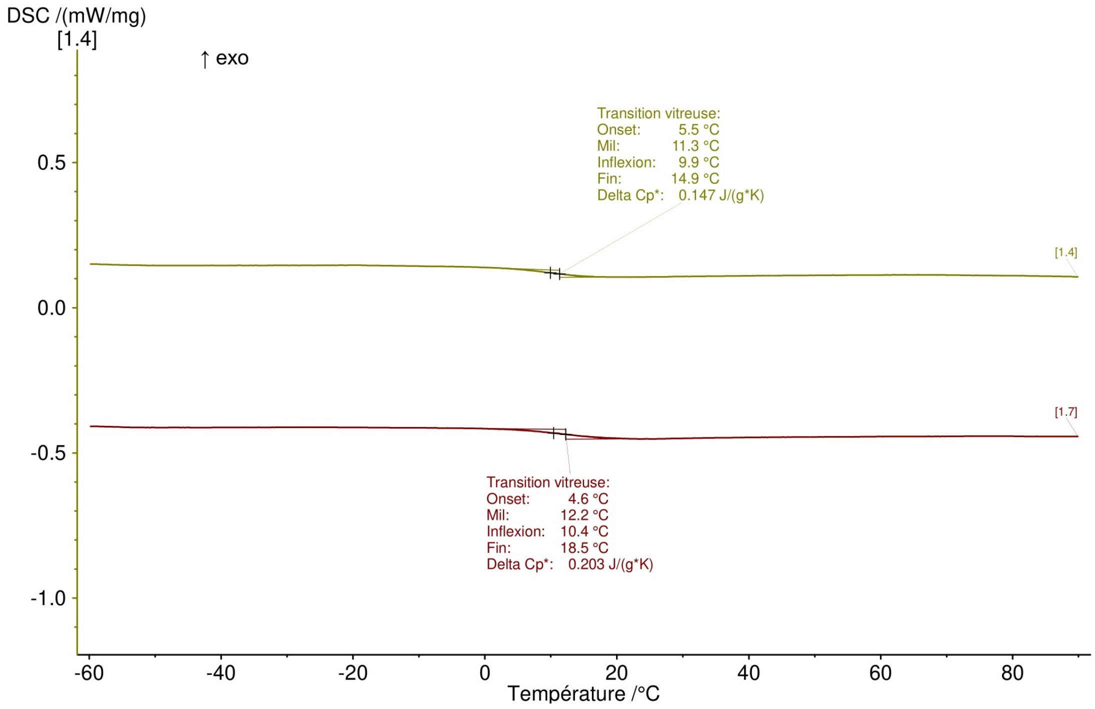

Figure S66: DSC of **25** inverse vulcanized with S_2Cl_2 at 65 °C.

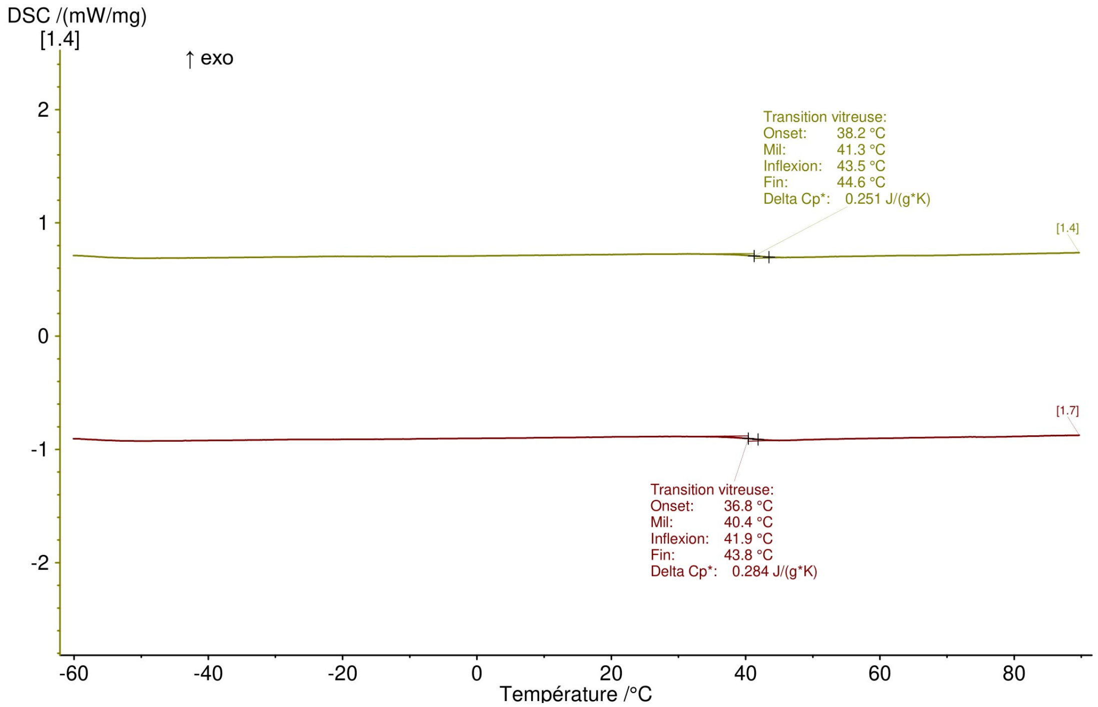

Figure S67: DSC of **25** inverse vulcanized with S_2Cl_2 at 70 °C under nitrogen.

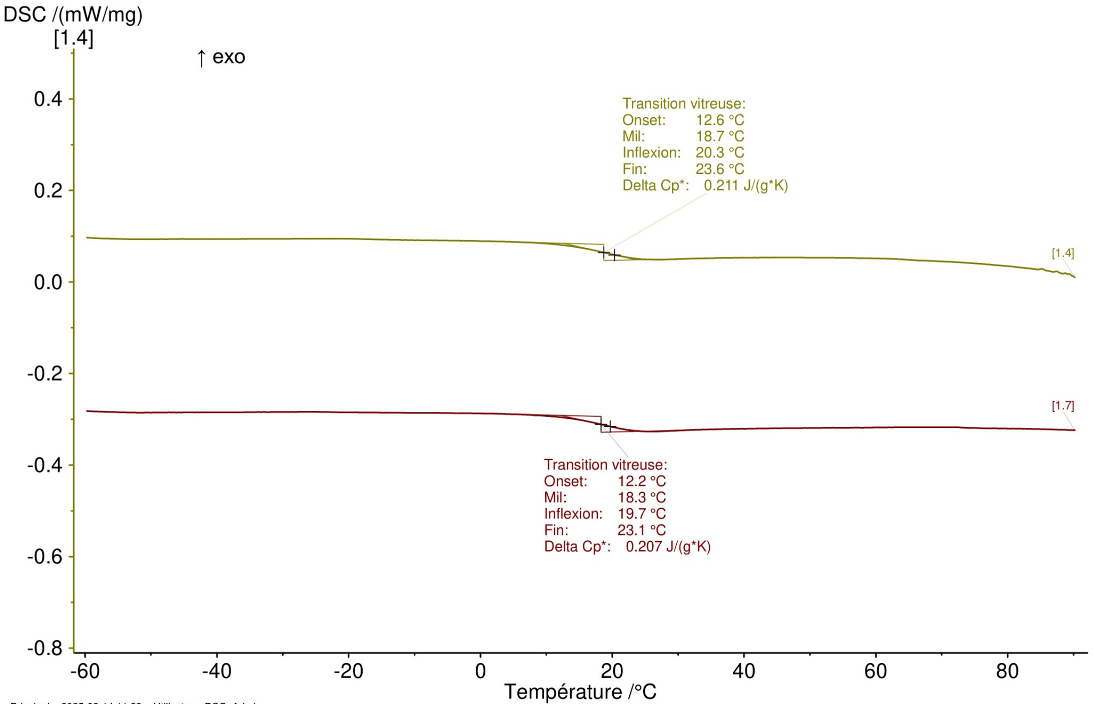

Figure S68: DSC of **27** inverse vulcanized with S_2Cl_2 at 70 °C.

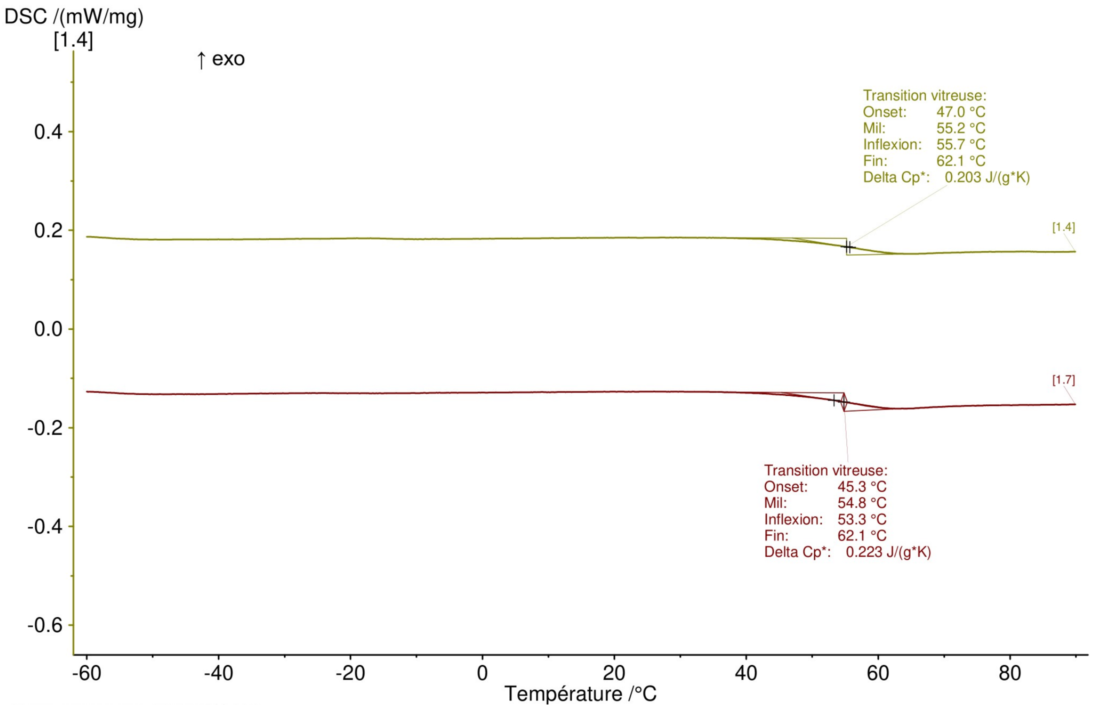

Figure S69: DSC of **27** inverse vulcanized with S_2Cl_2 at 70 °C under nitrogen.

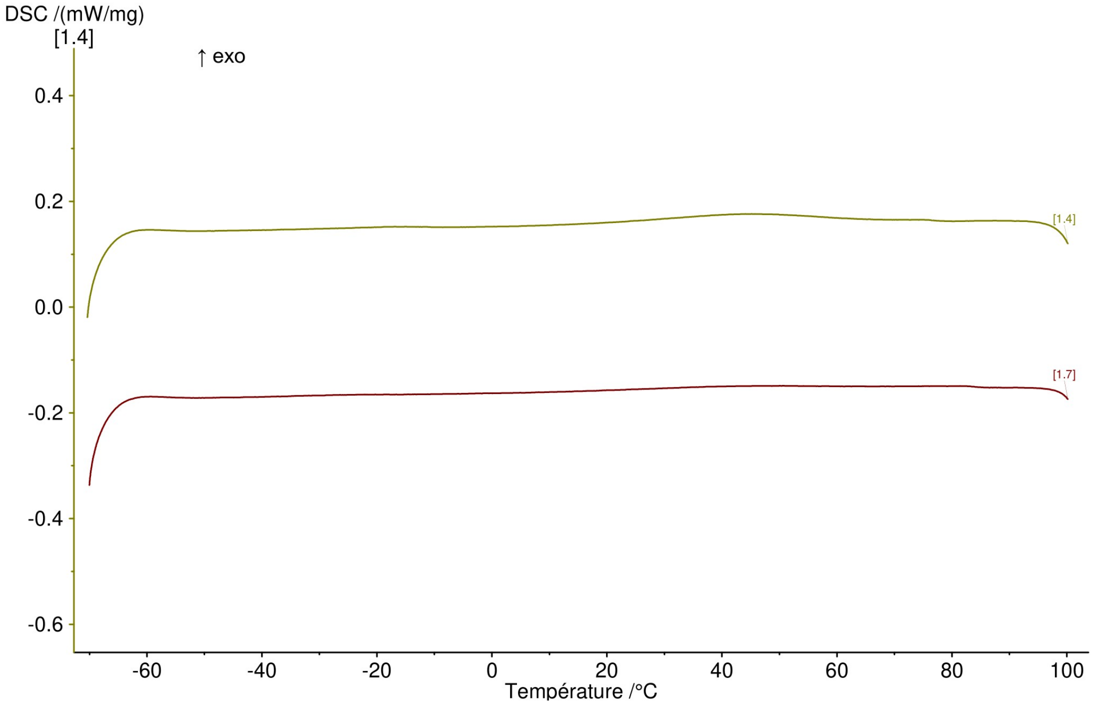

Figure S70: DSC of **25** inverse vulcanized with S_8.

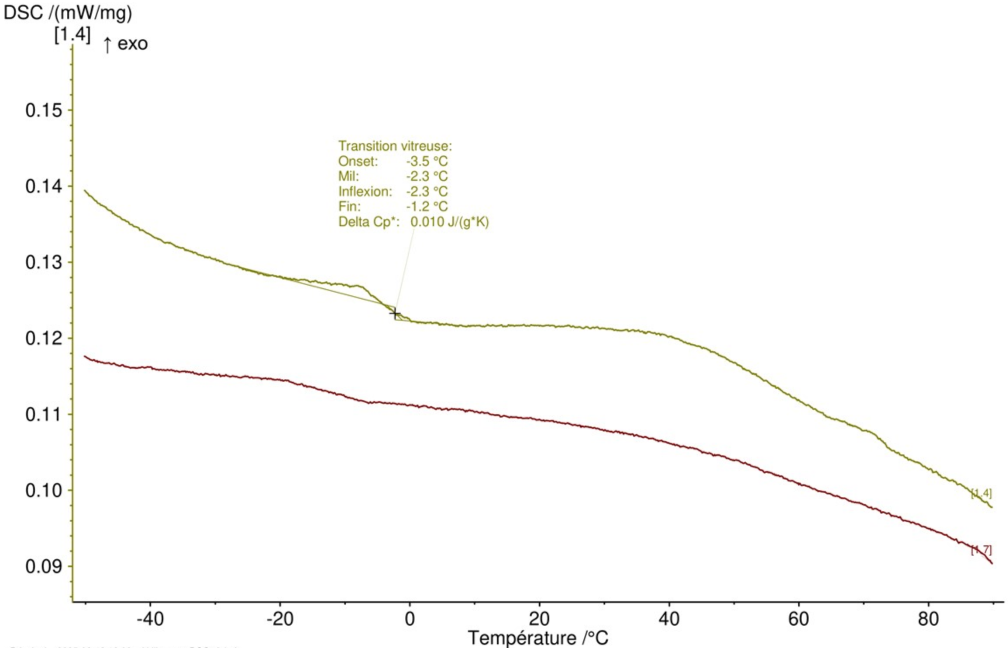

Figure S71: DSC of **26** inverse vulcanized with S_8.

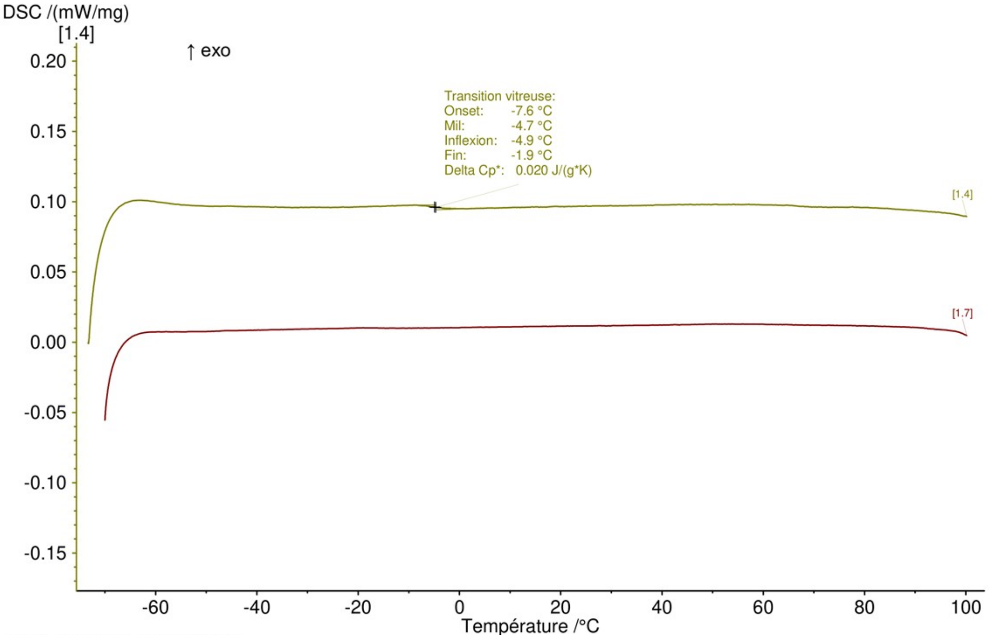

Figure S72: DSC of **27** inverse vulcanized with S_8.

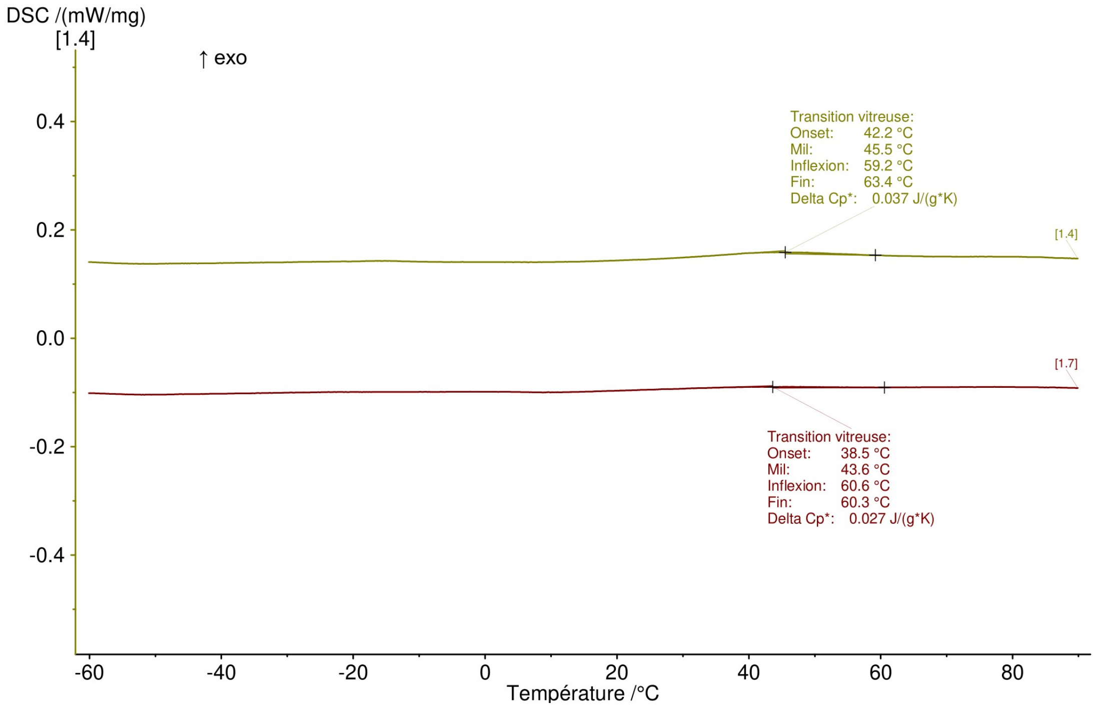

Figure S73: DSC of **28** inverse vulcanized with S_8.

Gel Permeation Chromatography:

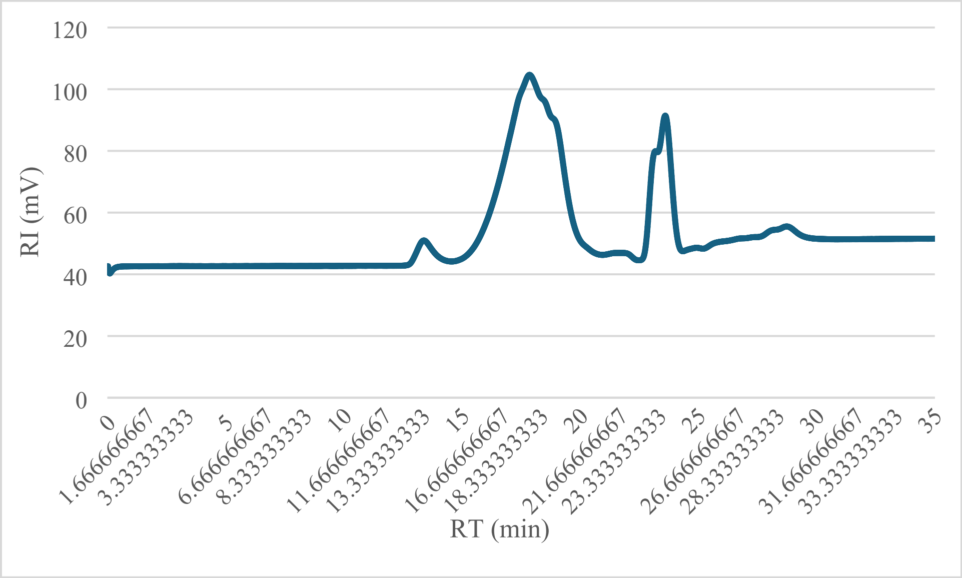

Figure S74: GPC trace of **25** inverse vulcanized with S_2Cl_2 at room temperature.

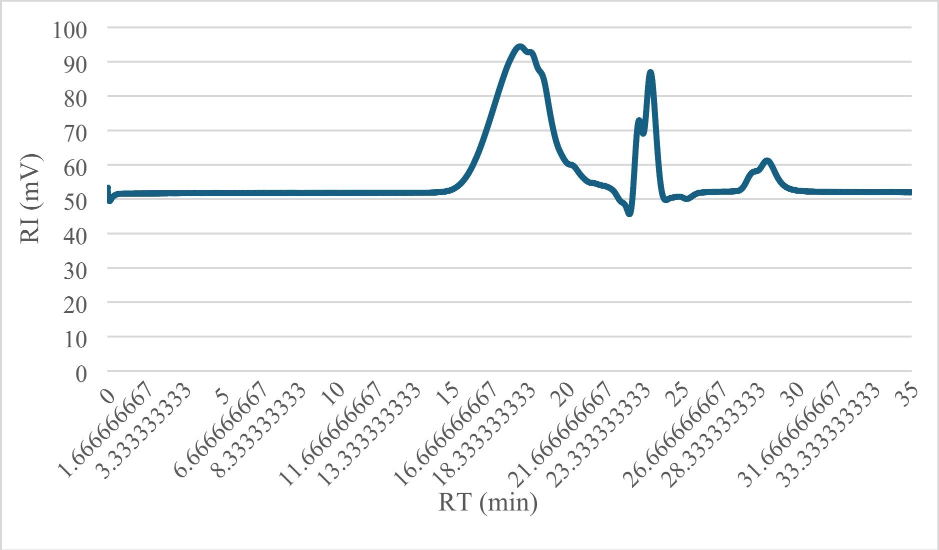

Figure S75: GPC trace of **25** inverse vulcanized with S_2Cl_2 at 65 °C.

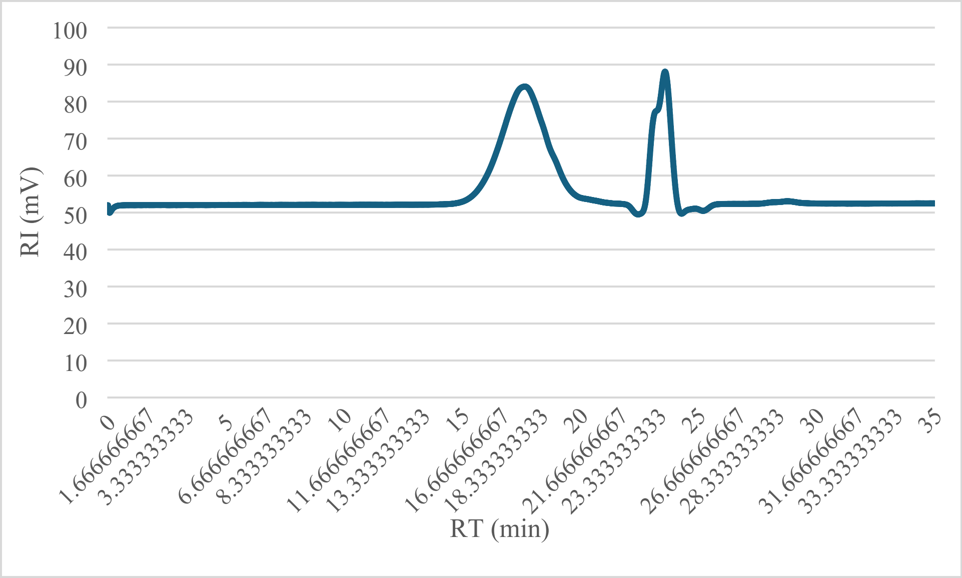

Figure S76: GPC trace of **25** inverse vulcanized with S_2Cl_2 at 70 °C under nitrogen.

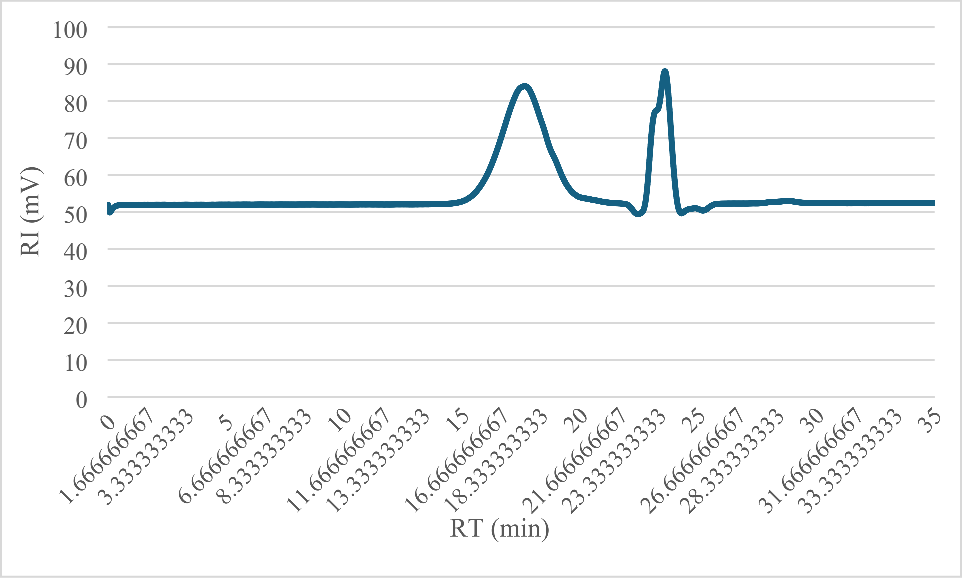

Figure S77: GPC trace of **27** inverse vulcanized with S_2Cl_2 at 70 °C.

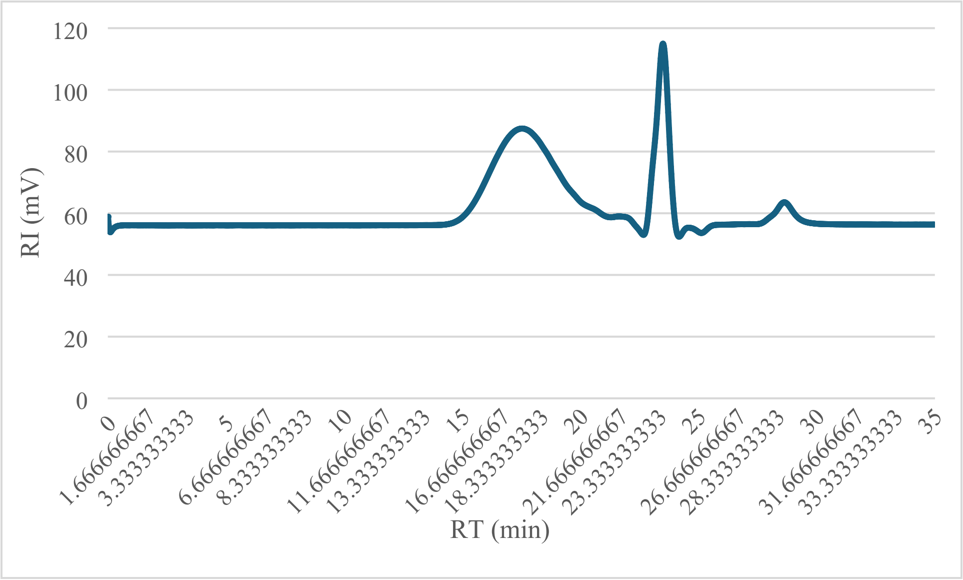

Figure S78: GPC trace of **27** inverse vulcanized with S_2Cl_2 at 70 °C under nitrogen.
